# Metagenome-assembled genomes from High Arctic glaciers highlight the vulnerability of glacier-associated microbiota and their activities to habitat loss

**DOI:** 10.1099/mgen.0.001131

**Published:** 2023-11-08

**Authors:** Melanie C. Hay, Andrew C. Mitchell, Andre R. Soares, Aliyah R. Debbonaire, Diana C. Mogrovejo, Nora Els, Arwyn Edwards

**Affiliations:** ^1^​ Department of Life Sciences (DLS), Aberystwyth University, Wales, UK; ^2^​ Interdisciplinary Centre for Environmental Microbiology (iCEM), Aberystwyth University, Wales, UK; ^3^​ Department of Geography and Earth Sciences (DGES), Aberystwyth University, Wales, UK; ^4^​ Dr. Brill + Partner GmbH Institut für Hygiene und Mikrobiologie, Hamburg, Germany; ^5^​ Department of Lake and Glacier Research, Institute of Ecology, University of Innsbruck, Innsbruck, Austria; ^6^​ Department of Arctic Biology, University Centre in Svalbard (UNIS), Longyearbyen, Svalbard and Jan Mayen; ^†^​ Present address: Department of Pathobiology and Population Sciences, The Royal Veterinary College, North Mymms, Hertfordshire, UK; ^‡^​ Present address: Environmental Metagenomics, Research Center One Health Ruhr of the University Alliance Ruhr, Faculty of Chemistry, University of Duisburg-Essen, Essen, Germany

**Keywords:** Metagenome-assembled genomes, Svalbard, glacier, cryoconite, co-factor metabolism

## Abstract

The rapid warming of the Arctic is threatening the demise of its glaciers and their associated ecosystems. Therefore, there is an urgent need to explore and understand the diversity of genomes resident within glacial ecosystems endangered by human-induced climate change. In this study we use genome-resolved metagenomics to explore the taxonomic and functional diversity of different habitats within glacier-occupied catchments. Comparing different habitats within such catchments offers a natural experiment for understanding the effects of changing habitat extent or even loss upon Arctic microbiota. Through binning and annotation of metagenome-assembled genomes (MAGs) we describe the spatial differences in taxon distribution and their implications for glacier-associated biogeochemical cycling. Multiple taxa associated with carbon cycling included organisms with the potential for carbon monoxide oxidation. Meanwhile, nitrogen fixation was mediated by a single taxon, although diverse taxa contribute to other nitrogen conversions. Genes for sulphur oxidation were prevalent within MAGs implying the potential capacity for sulphur cycling. Finally, we focused on cyanobacterial MAGs, and those within cryoconite, a biodiverse microbe-mineral granular aggregate responsible for darkening glacier surfaces. Although the metagenome-assembled genome of *Phormidesmis priestleyi*, the cyanobacterium responsible for forming Arctic cryoconite was represented with high coverage, evidence for the biosynthesis of multiple vitamins and co-factors was absent from its MAG. Our results indicate the potential for cross-feeding to sustain *P. priestleyi* within granular cryoconite. Taken together, genome-resolved metagenomics reveals the vulnerability of glacier-associated microbiota to the deletion of glacial habitats through the rapid warming of the Arctic.

## Data Summary

Sequencing data in the form of fastq.gz files used to generate the MAGs in this study can be accessed from the European Nucleotide Archive (ENA) project accession PRJEB59067. All software is in the public domain and linked to citations. Geochemical data are included as Table S14, available in the online version of this article within the supplementary information available online with this paper.

**Impact Statement**

Collectively, glaciers and ice sheets represent the largest freshwater ecosystems on Earth. Nevertheless, their austere conditions and microbially dominated communities mean that glacial ecosystems are often neglected, even within assessments of the impacts of glacier loss on associated ecosystems. Consequently, there is a paucity of genomic insights into the diversity, adaptations, evolution, and functionality of glacial ecosystems. Within this study we add to the modest number of studies applying genome-resolved metagenomics to explore glacial genomic diversity by comparing different habitats within catchments hosting glaciers on Svalbard. This High Arctic archipelago is the fastest warming region on Earth, warming at seven times the global average since 1979. It is also heavily (60 %) covered by glaciers. Its glacial ecosystems are therefore at high risk from climate change. Our results highlight the genomic diversity of microbiota associated with Svalbard glacial habitats and its capacity for mediating multiple processes within carbon and nutrient cycles. We also show taxonomic composition differences between habitats which suggests niche specialisation and highlights the vulnerability of these taxa to habitat change and loss. Our data suggest *Phormidesmis priestleyi,* the cyanobacterial keystone species acting as both primary producer and ecosystem engineer within granular cryoconite may be limited in its capacity for vitamin biosynthesis. We consider the possibility that other microbes bound within cryoconite serve as proximal sources of these essential micronutrients, thereby sustaining this cyanobacterial lineage on glacier surfaces. Considering the scarcity of *P. priestleyi* in other adjacent habitats, our results imply this supraglacial keystone species is particularly vulnerable to the loss of glacier surfaces.

## Introduction

The Arctic cryosphere is fading fast [1]. Consequently, ecosystems associated with High Arctic environments such as glaciers are changing rapidly [2]. Over recent decades, studies of the microbial ecology of High Arctic glaciers have underpinned our view of glaciers as ecosystems [3] which house substantial microbial biomass [4], diversity [5–7] and biogeochemical cycles [8–10]. Microbially-driven biogeochemical cycling has crucial implications for global change. For example, microbial consortia on ice and snow respond to and amplify glacier melting [11–14]. Moreover, glacier-associated microbial activities supply organic carbon and nutrients within catchments where these are acutely limited [15, 16]. Furthermore, microbes colonize forelands liberated by glacial recession, sustaining the development of new terrestrial ecosystems [17]. Understanding these intricate interactions between ice and life within a landscape of loss and change within the twenty-first century Arctic is therefore a priority [2, 18]. Nonetheless, the recognition that glacier loss has impacts on biodiversity is somewhat focused on downstream habitats [19], ignoring the fact that glaciers themselves host biodiverse ecosystems which will be obliterated by the loss of the glacier itself [20]. Consequently, improving our understanding of the genomic diversity of glacial ecosystems merits attention [2].

Metagenome-assembled genomes (MAGs) are genomes that are reconstructed from metagenomic DNA. Whereas MAGs have found utility in resolving the genomic diversity of host-associated, terrestrial, and marine habitats, their application to Arctic glacier habitats is still developing [21–23]. However, since essential functions within glacier-associated communities are often delivered by core and keystone taxa [24–26], the metabolic and genomic diversity revealed within catalogues of MAGs retrieved from the High Arctic in recent years promise key insights into microbially-mediated biogeochemical cycles and ecosystems.

Filamentous cyanobacteria are notable among the core and keystone taxa of glacier surface ecosystems, where they are frequently termed ‘ecosystem engineers’ [24]. Cyanobacterial photosynthesis offers the functional basis for primary production. Meanwhile their morphology and exudation of extracellular polymeric substances contributes to forming micro-environments for heterotrophic microbes [27, 28]. For these reasons, this phylum is the main bacterial component of cryoconite, a granular low-albedo microbe-mineral aggregate which is a major habitat on the glacial surface [29]. Cyanobacteria are also among the first to colonise recently deglaciated forefields where they aid in the development of soils [30]. Indeed, *Phormidesmis priestleyi* is the dominant species in cryoconite from Midtre Lovénbreen, Austre Brøggerbreen [31] and Foxfonna in Svalbard [24], Greenland [26, 32–34] and elsewhere in the Arctic [35]. Although the single cultured isolate affiliated to *Phormidesmis priestleyi* from cryoconite was isolated in Greenland [32], this lineage was notably missing from a later study of Greenland cryoconite metagenomes, where *Nostoc* has been recovered as the only cyanobacterial bin [21].

Recently, *Phormidesmis* MAGs recovered from Arctic cryoconite were described that lacked genes coding for phycoerythrin, a light-harvesting protein-pigment complex present in cyanobacteria from glaciers in lower-latitude regions [23]. Differences in genome coverage, and persistent difficulties assembling the genomes despite great sequencing depth suggest that there may be significant strain heterogeneity within Cyanobacteria. If so, it is possible that different genes may be deleted from the genomes of geographically distant strains. An improvement in the understanding of the geographical ranges and genomic diversity of cryoconite-associated cyanobacteria is therefore required.

Within this study we sought to explore the genomic diversity present within key habitats in High Arctic glacier catchments, focusing on cryoconite and forefield soils of glaciers on the shores of Kongsfjorden in the High Arctic archipelago of Svalbard. The region is well studied in terms of its microbial ecology [5, 17, 36–40] and is regarded as a model site [41]. The future of glaciers in this region is bleak [42], as Svalbard represents the fastest-warming region of the Arctic [43]. Beyond simply developing an inventory of MAGs, we aimed to understand the bacterial and archaeal genomic diversity associated with different habitats, and to examine the linkages between the genomes of key players in glacier ecosystems and their contribution to key biogeochemical cycles. Furthermore, we wished to gain an insight into the genomic basis of microbial interactions associated with cryoconite granules for they represent a major locus of biodiversity and activity on glacier surfaces.

Our study reveals distinctive microbial genomes associated with discrete habitat types in the glacierized catchments mediating a broad range of carbon and macronutrient cycling processes. This raises the prospect that the destruction of specific glacier-associated habitats as the catchments lose their glaciers may contribute to the derangement of terrestrial biogeochemical cycling [20] in this rapidly warming region of the Arctic [43]. Moreover, the multiple auxotrophies in essential cofactor metabolism inferred from the absence of biosynthetic capacity for these essential cofactors within the genome of the cryoconite ecosystem engineer *Phormidesmis priestleyi* implies that commensal interactions sustain this taxon within the confines of cryoconite granules. The dissipation of granular cryoconite ecosystems due to glacier loss could therefore modulate the fitness of *Phormidesmis priestleyi,* thereby endangering the lineage’s future within the warming landscape of the High Arctic.

## Methods

### Study site

**Fig. 1.**
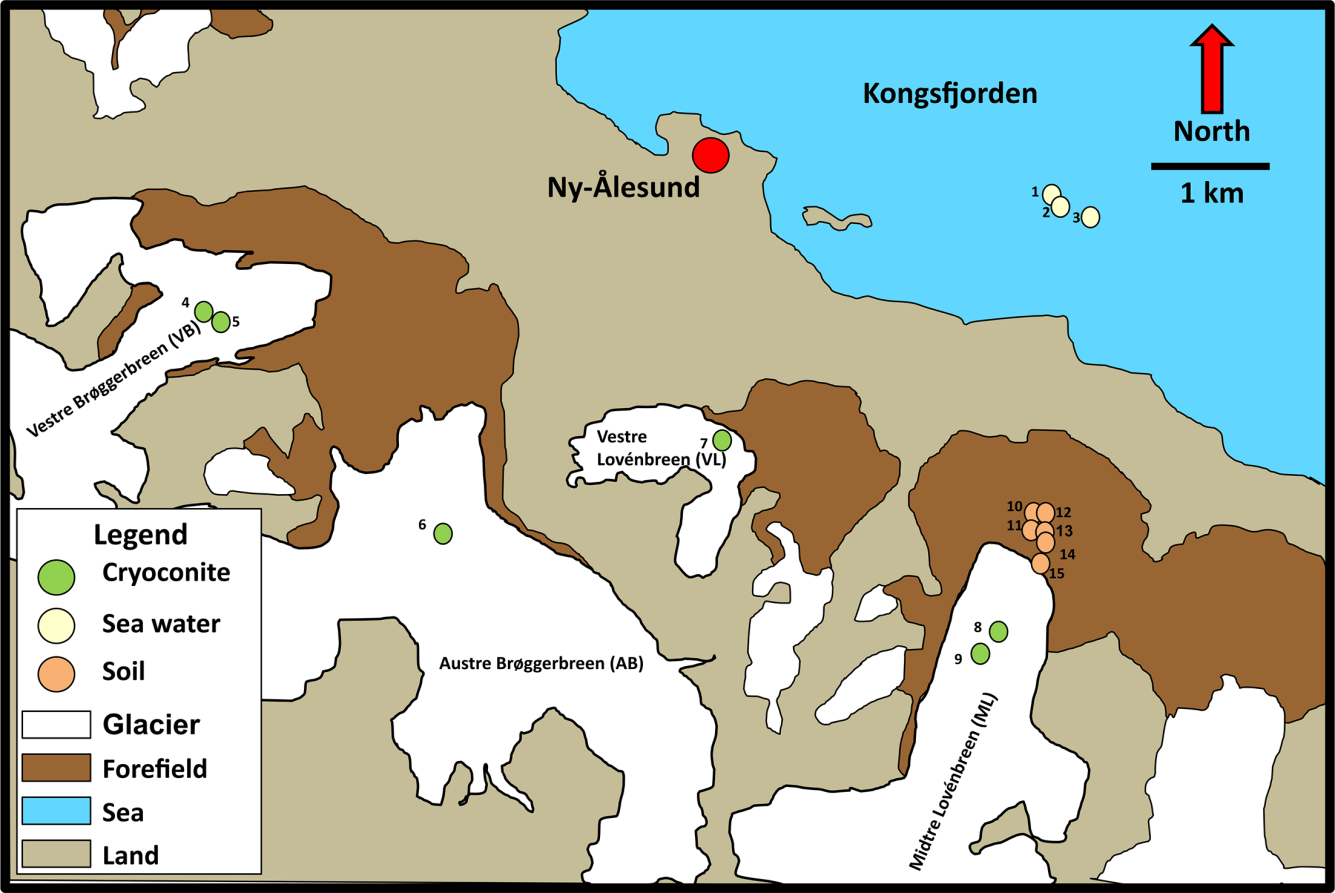
Map of sampling sites for shotgun libraries included in this study. Glaciers are abbreviated: AB: Austre Brøggerbreen, ML: Midtre Lovénbreen, VB: Vestre Brøggerbreen and **VL:** Vestre Lovénbreen. Sample site key: 1: SS1, 2: SS2, 3: SS3, 4: VB_18, 5: VB_17, 6: AB_18, 7: VL_18, 8: ML_18, 9: ML_17, 10: F1T4, 11: F1T3, 12: F2T4, 13: F3T3, 14: F2T2, 15: F3T1.

Samples of seawater from Kongsfjorden Fjord, cryoconite from Austre Brøggerbreen (AB), Midtre Lovénbreen (ML), Vestre Brøggerbreen (VB) and Vestre Lovénbreen (VL) glaciers and proglacial soils from the moraines in front of ML were collected from Ny-Ålesund. The cryoconite (*n*=6) was collected from the surfaces of ML and VB glaciers in the summer of 2017 (ML-17, VB-17) and 2018 (ML-18, VB-18) respectively. Cryoconite from VL (VL-18) and AB (AB-18) were also collected in 2018. From 15 forefield soil sites (made up from three transects of five timepoints) samples from a subset of six sites were sequenced. The samples are named using the FxTy, where x refers to the transect and y refers to the time point. The three seawater samples (SS1, SS2 and SS3) were collected from the neighbouring fjord. The location and types of samples included in this study are shown in Fig. 1 and Table S1. Samples were stored cold and dark during transfer on foot (within 2–3 h) to the NERC Station in Ny-Ålesund. Thereafter the soil and cryoconite samples were stored at −20 °C prior to extraction while the seawater was immediately filtered aseptically using Sterivex 0.22 µm-filters which were then filled with RNALater and stored frozen until further processing.

### DNA extraction and sequencing

DNA from collected seawater biomass was extracted from Sterivex 0.22 µm-filters using the Qiagen DNEasy Sterivex PowerWater kit (Qiagen, Inc.). DNA from cryoconite was extracted using the Qiagen DNEasy PowerSoil kit (Qiagen, Inc.), and DNA from soil samples was extracted using the MP Biomedicals FastDNA Spin Kit for Soil (MP Biomedicals, Inc). Obtaining sufficient DNA yields from certain proglacial soil samples with a high ratio of mineral surfaces to biomass proved challenging using these standard methods. For one soil sample (F3T3), we compared the FastDNA (F3T3_FD) extraction to two other methods which used a higher starting biomass. The MO BIO PowerMax Soil DNA Isolation Kit (MO BIO, Inc) used 10 g starting mass (F3T3_PM). We also tried an indirect DNA extraction in which density gradient centrifugation using Ludox HS-40 (Sigma-Aldrich, Inc) was used to separate biomass from mineral surfaces (60 g soil) followed by lysis using the Epicentre MasterPure Complete DNA and RNA extraction kit (F3T3_Lud). DNA extraction methods and yields are provided in Supplementary Information 1, and Table S2. Sequencing was performed using the Illumina Nextera Kit according to manufacturer’s instructions (Illumina, Inc). The pooled libraries were then sequenced at Wales Gene Park (Cardiff University) using an Illumina NextSeq in High Output mode, generating 2×150 bp paired end reads.

### Metagenome data processing

Paired-end reads were uploaded to KBase (https://www.kbase.us/ [44], assessed using FastQC [45] and trimmed using Trimmomatic [46] to remove adapters and low-quality reads. The quality-controlled libraries for each environment type were combined and co-assembled on KBase using MEGAHIT v1.1.1 [47]. Reads-based taxonomic assignment was performed on KBase using Kaiju (v.15.0) in Greedy mode, against the NCBI blast nr +EUK database [48].

### Recovering genomes from metagenomes

Most analyses were conducted on virtual machine instances hosted on servers at CLIMB (https://www.climb.ac.uk/ [49]). MAGs were constructed using anvi’o (v 6.1 –Esther) [50]. A contigs database was created from the assembled contigs by running *anvi-gen-contigs-database* which and identifies open reading frames (ORFs) using Prodigal [51]. To estimate the number of bacterial genomes in the metagenome, anvi’o runs HMMER against a single-copy gene databases that included Bacteria, Candidate Phyla Radiation (CPR) Bacteria, Archaea, Protista. The contigs were functionally annotated with NCBI COG functions [52], KEGG pathway information [53], and eggNOG information [54]. Predicted genes and contigs were taxonomically classified using Kaiju [48]. A contigs database of 162 105 contigs (divided into 163 249 splits) was binned using *anvi-cluster-contigs* as part of the anvi’o workflow using CONCOCT v1.1.0 [55], MaxBin2 v2.2.7 [56] and MetaBAT2 v2.12.1 [57]. DAS Tool v1.1.2 [58] was then run on all three bin collections, using DIAMOND as a search engine, and the default score-threshold [0.5]. The dereplicated bins from the DAS Tool collection were then manually refined using *anvi-refine* from the anvi’o toolkit. To aid in refinement, the resulting bins from each tool were exported as collections, merged to create a data-frame of bin-association per contig and exported as a data layer into anvi’o (details in Supplementary Information 2). *Anvi-interactive* was run with the collection DAS Tool, ordered by mean coverage, and viewed using ‘detection’ which shows proportion of the contig (or bin) which has at least 1×coverage. To screen bins for Candidate Phyla Radiation (CPR), which often contain reduced genomes [59], *anvi-script-gen-CPR-classifier* was run on the Campbell taxonomy [60]. The refined MAGs were exported and the contigs were uploaded to KBase for quality checking using CheckM (https://github.com/Ecogenomics/CheckM [61]). The MAGs were also submitted to GTDB-Tk (GTDB-Tk v1.7.0; GTDB v R06-RS202) on KBase for high-resolution taxonomic assignment [62]. GTDB-Tk uses the same criteria of relative evolutionary divergence (RED) and average nucleotide identity (ANI) for establishing taxonomic ranks as the recently proposed rank-normalized taxonomy in GTDB [63]. MAGs with >0.95 ANI were classified to the species level, the remainder were named according to the lowest taxonomic rank.

### Phylogenomics

The phylogenomic relationship of the MAGs was compared by following the tutorial: http://merenlab.org/tutorials/infant-gut/#chapter-iii-phylogenomics. Briefly, the HMM hits from a curated collection of single-copy core genes (Bacteria_71, Table S3) for the GToTree workflow [64] were compared used to compare the MAGs in a phylogenomic analysis. To do this, the selected genes from each MAG in the collection were concatenated, translated and aligned by running *anvi-get-sequences-for-hmm-hits* with default settings plus the additional parameters: (--concatenate-genes --return-best-hit --get-aa-sequences). The *anvi-get-sequences* command uses muscle to align the genes [65]. The phylogenomic tree was calculated using *anvi-gen-phylogenomic-tree*, which infers approximately-maximum-likelihood phylogenetic trees from FASTA files using FastTree [66].

### Spatial comparisons and biogeochemical cycling

The spatial distribution of MAGs was visualised using heatmaps of max-normalised ratio (number of reads recruited to a contig divided by the maximum number of reads recruited to that contig in any sample) and abundance (mean coverage of each MAG divided by that sample’s overall mean coverage across all the MAGs) [50]. Heatmaps were created using the R package ComplexHeatmap (http://www.bioconductor.org/packages/devel/bioc/html/ComplexHeatmap.html) [67]. The FASTA files of MAGs were analysed for major metabolic pathways using MetabolisHMM (https://github.com/elizabethmcd/metabolisHMM) [68]. In this analysis, the FASTA files of the genomes were formatted, annotated using Prodigal and then a HMMER search was run against a curated list of genes involved in important biogeochemical pathways (Table S4) [69].

### Cyanobacterial comparative genomics

A pangenome of *Phormidesmis* genomes was constructed using nine genomes downloaded from NCBI Genbank and RefSeq (Table S5) by searching for complete *Phormidesmis* genomes on the GTDB database (https://gtdb.ecogenomic.org/). These were compared to the MAGs resolved in this dataset (Table S6) using a previously described workflow (http://merenlab.org/2016/11/08/pangenomics-v2/) and (http://merenlab.org/tutorials/infant-gut/#chapter-iv-pangenomics). Reference genomes were downloaded from NCBI, and the FASTA files were converted to a contigs database in the anvi’o workflow using *anvi-script-FASTA-to-contigs-db*. Scripts *anvi-run-hmms* and *anvi-run-ncbi-cogs* were then run to make the external genomes comparable to the MAGS. The internal MAGs and external genomes downloaded from GTDB were made into a genomes storage database that stores information about genomes using *anvi-gen-genomes-storage*. The pangenome was generated by running *anvi-pan-genome*, using BLASTp and MCL [70]. The average nucleotide identity (ANI) of the MAGs and external genomes was calculated within anvi’o using PyANI [71].

## Results

Shotgun metagenome libraries were created using paired-end Illumina sequencing (sequencing statistics in Table S7). The resulting MEGAHIT co-assembly was 720 998 358 bp in size and consisted of 162 105 contigs over 2000 bp (Table S8).

### Reads-based taxonomy of glacier-associated metagenomes

**Fig. 2.**
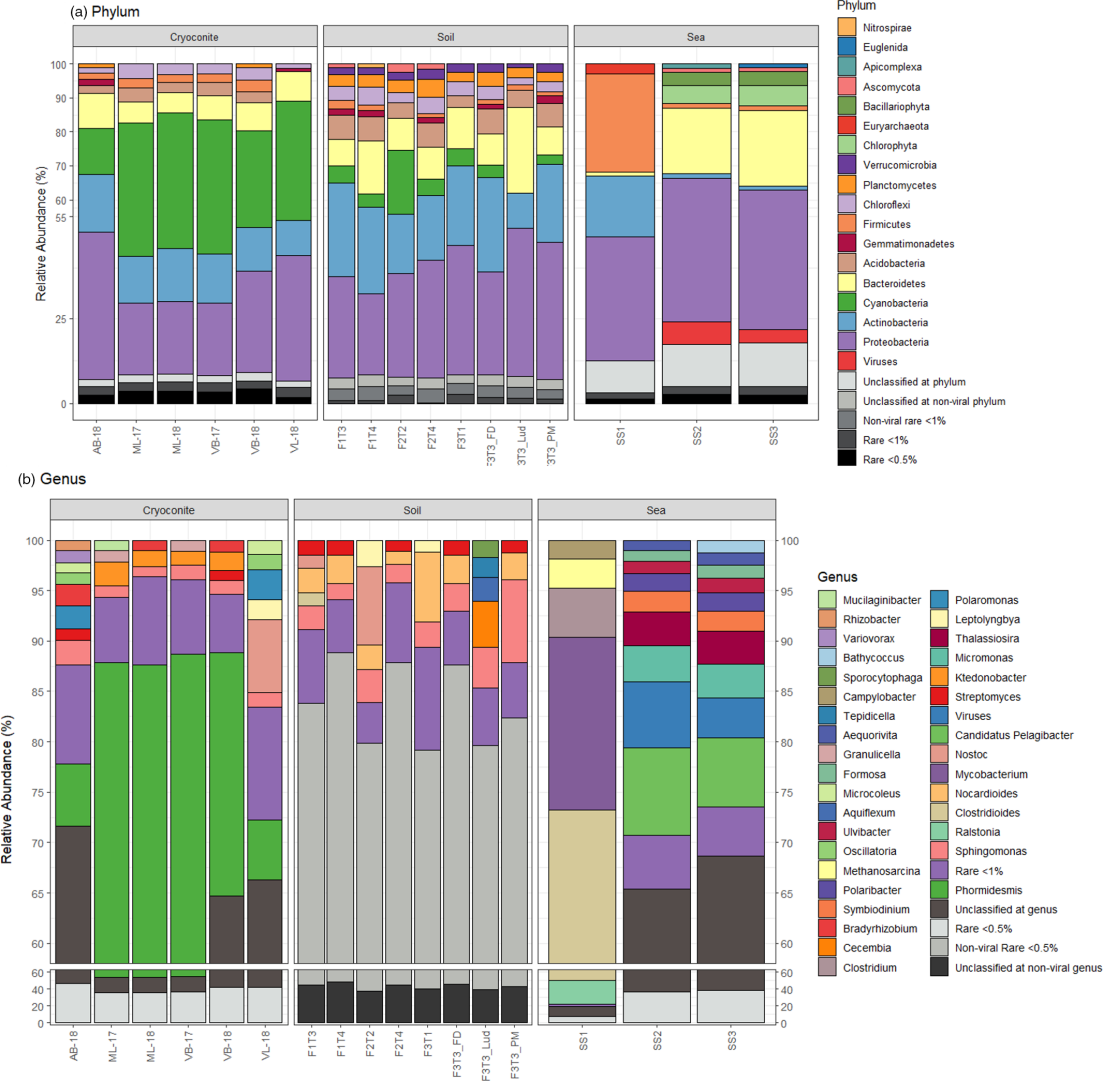
Reads-based taxonomic assignment of metagenomics libraries at the (a) Phylum and (b) Genus level using Kaiju.

To first provide an unbiased overview of the taxonomic composition of the metagenomes the taxonomic composition of unassembled reads is presented (Fig. 2, Table S9). Approximately 60 % of reads from cryoconite and soil samples could be successfully classified whereas the percentage of reads classified from seawater samples was both lower and more variable (11–45 %).

Within cryoconite, most classified reads were aligned to Cyanobacteria, followed by Proteobacteria, Actinobacteria and Bacteroidetes as the major bacterial phyla, with Ascomycota as the major eukaryotic phylum (Fig. 2a). At genus level, the VB library and ML library from 2017 and 2018 resemble each other closely (Fig. 2b). *Phormidesmis* is the most abundant genus in the ML-17 (33.92 %), ML-18 (33.81 %), VB-17 (33.69 %) and VB-18 (24.12 %) samples. Although members of *Phormidesmis* are present in AB (6.23 %) and VL (6.00 %), they represent a smaller fraction of the community. Other genera present in significant numbers include *Ktedonobacter* which were found in ML-17, ML-18, VB-17, and VB-18. VL presented a high proportion of *Nostoc* (7.28 %) which was present at higher abundance than *Phormidesmis* (6.00%). Across the soil transects, Proteobacteria, Actinobacteria, and Bacteroidetes were dominant, with contributions from eight other phyla including Cyanobacteria, whereas Ascoymycota were less prominent within the metagenomes. The most abundant genera – also common to all sample sites – were *Sphingomonas, Nocardioides, Streptomyces* and *Bradyrhizobium *.

Seawater samples (SS1, SS2 and SS3) were sequenced to provide representation from a contrasting downstream environment. Viruses were abundant in the SS2 (6.53 %) and SS3 (3.96 %) samples, and less abundant in SS1 (0.38 %). The most abundant bacterial phyla in all samples were the Proteobacteria, (range 36.56–42.28 %), with a high abundance of Bacteroidetes in SS2 (19.25 %) and SS3 (22.36 %) and a lower proportion in SS1 (1.13 %). Chlorophyta, Bacillariophyta, Archaea (Euryarchaeota and Crenarchaeota), Fungi (Ascomycota, Basidiomycota), Apicomplexa, and Euglenida were notably represented. At the species level, SS1 differs from SS2 and SS3. Interestingly, SS1 contained a high abundance of species commonly associated with faecal-influenced environments. *Methanosarcina mazei* (1.9 %), an anaerobic archaeon is found in sewage tanks and anoxygenic, moist soil. *Campylobacter jejuni* (1.6 %) is commonly found in animal faeces and is a leading cause of food-borne illness in humans.

### Recovery of metagenome assembled genomes from co-assembly

The soil, cryoconite and seawater shotgun libraries were assembled in a co-assembly of all environments, as that provided extra information about the taxonomy of genomes that are shared between environments, with minimal loss of information about genomes that are unique to one environment (or even one sample) in particular (Table S8). Assembled contigs were clustered into 151 bins using CONCOCT (v 1.1.0), 180 bins using MaxBin2 (version 2.2.7), and 225 bins using metaBAT2 (v 2.12.1). The bins in each collection were compared using DAS Tool (v 1.1.2) and a dereplicated collection of 95 bins with completion higher than 50 % was created. Following manual curation, the final collection consisted of 74 MAGs. (Table S10). The depth of coverage of the MAGs ranged from 11 X to 655 X (Table S11) and the GTDB-Tk classification of MAGs is available in Table S12.

**Fig. 3.**
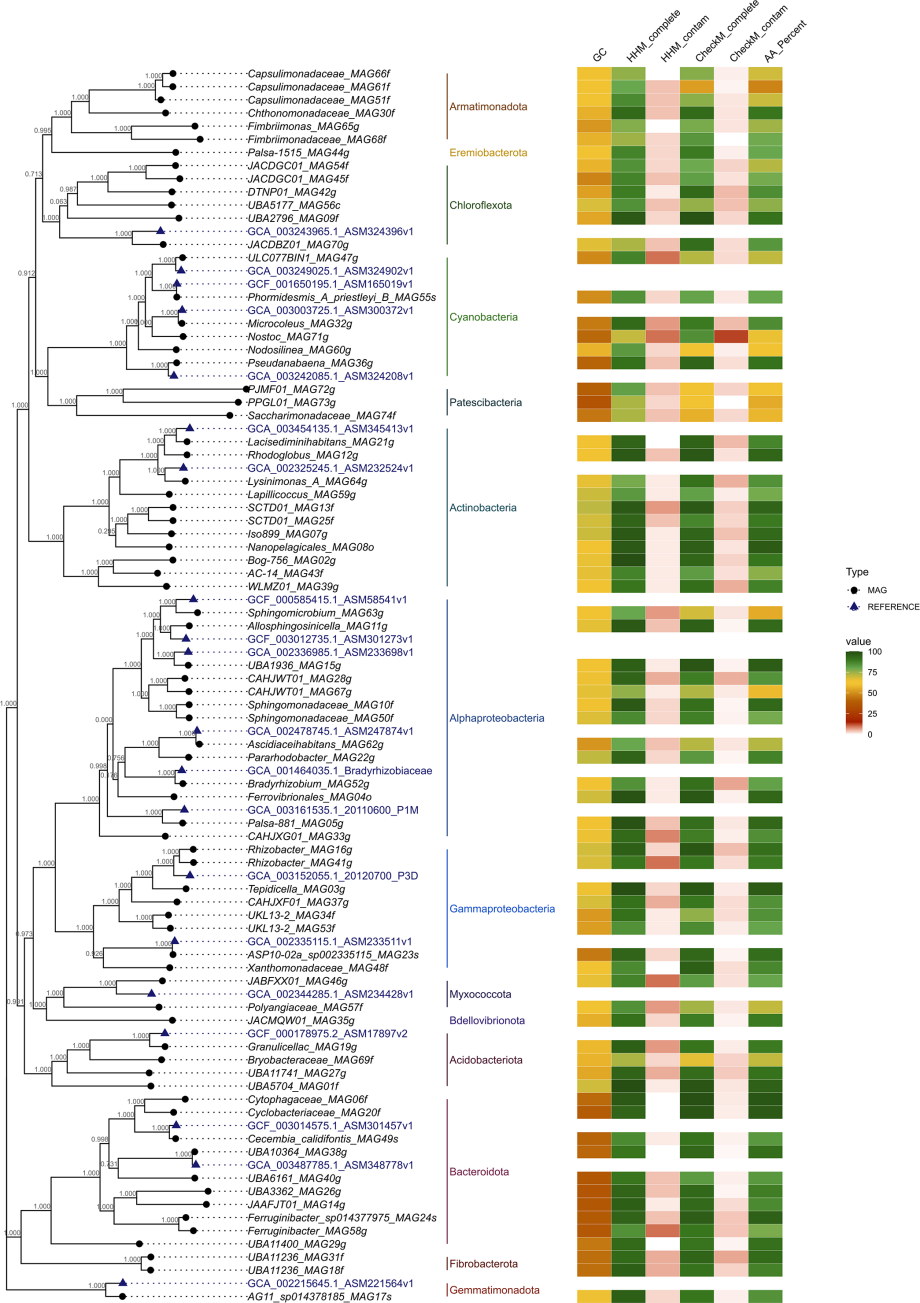
Phylogeny of MAGs created by Fast Tree of Muscle alignment of 71 single copy core genes.

Fig. 3 presents the taxonomic distribution of MAGs present within the collection. In order of abundance, these were the Proteobacteria (Alpha- = 13, Gamma- =8), Actinobacteriota (11), Bacteroidota (10) and the Cyanobacteria (6). The Chloroflexota (6), Armatimonadota (6), Acidobacteriota (4) and Patescibacteria (3) also had several members. Finally, we resolved two MAGs each from the Myxococcota and Fibrobacterota, and a single MAG member from Bdellovibrionota, Eremiobacterota and Gemmatimonadota. Five MAGs were assigned to species-level using FastANI within the GTDB-TK [62] with ANI >95 % and an alignment fraction >65 %. *Phormidesmis_A_priestleyi_B_MAG55s* is very similar (99.29 % ANI) to the *Phormidesmis priestleyi* strain BC1401, isolated from cryoconite on the South-western Greenland Ice Sheet [32]. *ASP10-02a_sp002335115_MAG23s* is very similar (95.44 % ANI) to an Oceanospirillaceae bacterium UBA2001 isolated from seawater marine metagenome from the North Sea [72]. *Ferruginibacter_sp014377975_MAG24s* (99.04 % ANI) is similar to *Ferruginibacter* sp014377975 (GCA014377975.1), and *AG11_sp014378185_MAG17s* is very similar (96.39 % ANI) to AG11 sp014378185 (GCA014378185.1) found in the Little Firn glacier in Greenland [73]. The remainder of the MAGs were assigned to novel species within their genus, family or order (Table S13).

### Read mapping co-assembled MAGs reveals a common cryoconite community but spatial differentiation in forefield soil communities

**Fig. 4.**
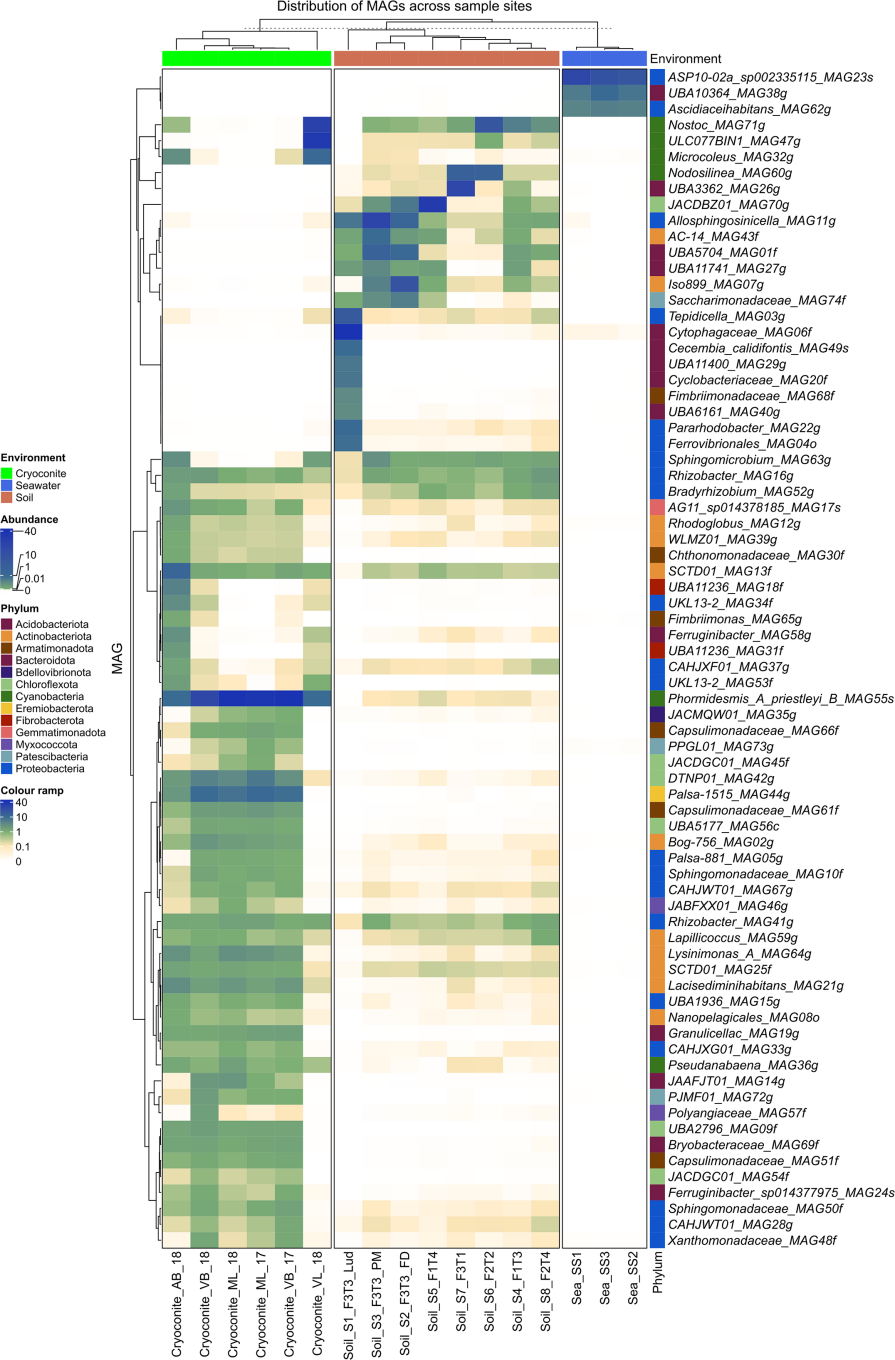
Heatmap showing the distribution of Svalbard MAGS based on abundance. Colour guide: green: MAGs close to sample mean coverage; blue: MAGs are highly abundant (10–40× the sample mean); wheat: rare MAGs (0.1 of the sample mean); white: MAGs recruited zero reads in that sample. The phylum membership of MAGs is displayed using a colour legend and environments were forced to cluster together.

Fig. 4 shows the plotted abundance values of each of the MAGs across the different sites. This visualisation is particularly useful for highlighting the highly abundant (green to blue) MAGs compared to the rare MAGs (wheat and white). By clustering the MAGs by site, groups of commonly co-occurring bacteria were identified (Figs 4 and S1). MAGs with habitat preference for sea, cryoconite and soil were identified.

As seen in Fig. 4, the fewest MAGs were found in seawater and not shared with other environments. Most of the MAGs are from the cryoconite samples, and patterns of co-occurrence are evident. Notably, the ML and VB samples are very similar to each other and have a large heterotrophic community of Actinobacteria and Proteobacteria. AB lacked several species found on VB and ML. The most striking observation was that MAGs in the VL cryoconite sample were very different to those in the cryoconite from ML, VB, and AB. Specifically, the VL community was missing many of the members that make up the cryoconite communities on other glaciers, and instead, contained taxa more commonly found in glacial forefield soil.

There are several MAGs (dark blue) that are highly dominant community members at specific sites. *Phormidesmis_A_priestleyi_B_MAG55s* was the most abundant MAG in the cryoconite samples, especially from ML and VB, however it is also found in high abundance on AB and VL and is present at low abundance in soil samples. VL had three highly abundant cyanobacterial MAGs that are absent or extremely rare in samples. Two of these MAGs, *Nostoc_MAG71g* and *Microcoleus_MAG32g* are also present in AB. In contrast, *ULC077BIN1_MAG47g*, was not found in any of the other cryoconite samples but is found in most soil samples. Another Cyanobacterial MAG (*Nodosilinea_MAG60g*) was highly abundant in two early soil samples (F3T1 and F3T2) and was present, though less abundant, in later soils. Five MAGs belonging to Chloroflexota were found exclusively in cryoconite, while one (*DTNP01_MAG42g*), was a low abundance member of all soil samples.

### Genome-resolved insights to major glacial biogeochemical cycles

**Fig. 5.**
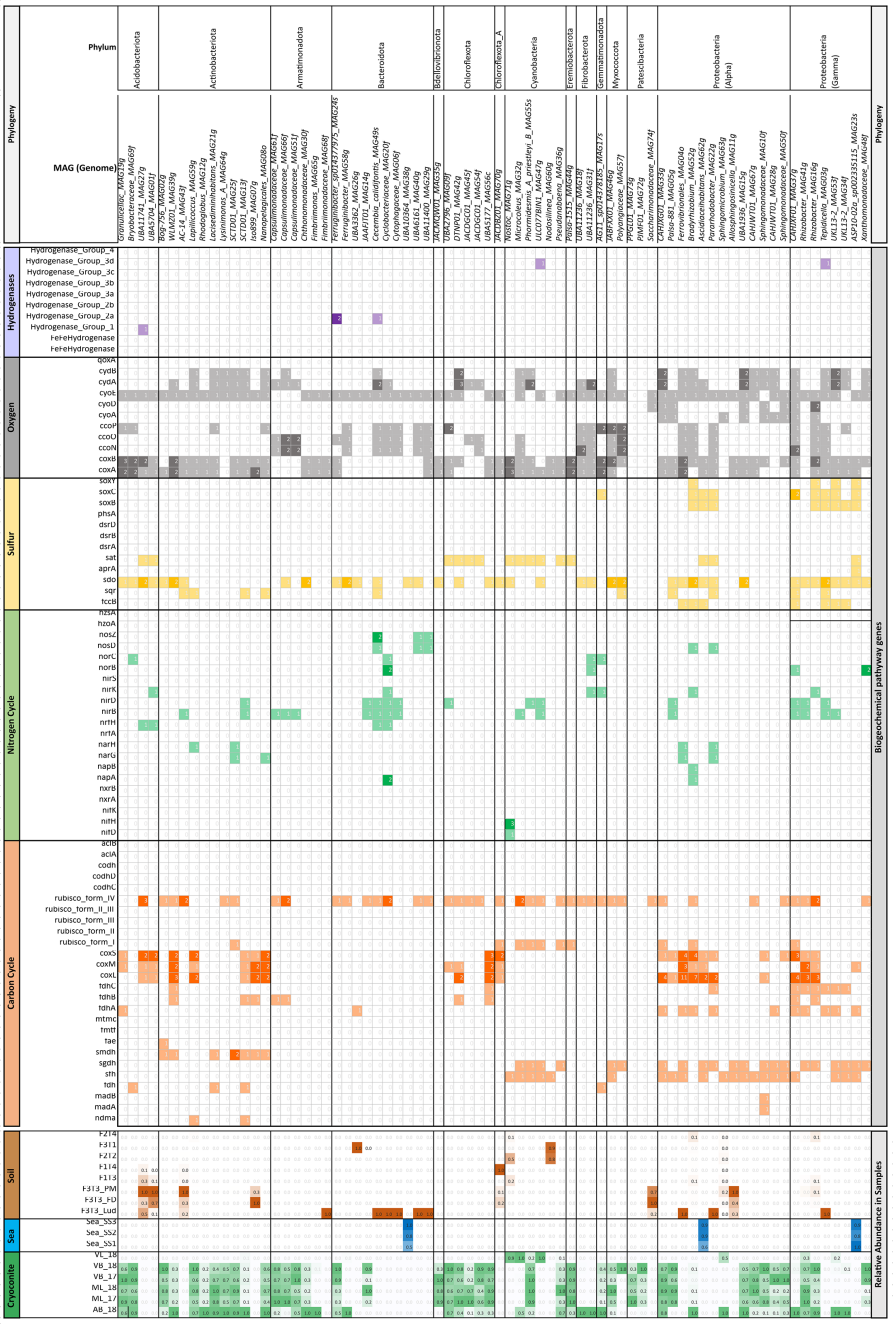
The detection of key genes involved in biogeochemical cycling within the collection of MAGs using MetabolisHMM with the strictest possible thresholds in the form of trusted cutoff values which the value is above all known false positives. Fig. S2 shows genes annotated using the less stringent default settings.

Microbially mediated carbon and nutrient cycling associated with glaciers can impact on local or regional biogeochemistry. Geochemical analysis of elemental concentrations within cryoconite and forefield soil underline this point (Table S14). For example, the highest organic carbon and nitrogen concentrations in samples from ML are found supraglacially, with cryoconite comprising 2.2 % organic C and 0.2 % nitrogen compared to 0.1 % organic C and 0.02 % nitrogen within forefield soil. We therefore sought to explore the genomic potential for key biogeochemical cycles. The tool metabolisHMM was used to examine metabolic genes present within MAGs for potential contributions to glacial biogeochemical cycles of carbon, nitrogen, and sulphur (Fig. 5).

*Carbon:* several MAGs had copies of form I RubisCO, including an Actinobacteria, *SCTD01_MAG25f*, from the family Frankiaceae, two Alphaproteobacterial MAGs, *CAHJXG01_MAG33g* and *Bradyrhizobium_MAG52g* belonging to the family Acetobacteraceae and Xanthobacteraceae respectively as well as a Gammaproteobacterial MAG *CAHJXF01_MAG37g*, family Burkholderiaceae. The MAG *JACDBZ01_MAG70g* from the phylum Chloroflexota and *Palsa-1515_MAG44g* from the phylum Eremiobacterota also contained copies.

There were also Form I RubisCO genes in all the cyanobacterial MAGs, except for *Nostoc_MAG71g* and *Nodosilinea_MAG60g*. Interestingly, all MAGs encoding RubisCO form I genes were most abundant in cryoconite, with the sole exception of *JACDBZ01_MAG70g*, which was more abundant in soil. Several methylotrophs were identified that had NDMA-dependent methanol dehydrogenase (*nmda*) (Fig. S3) and S-(hydroxymethyl)mycothiol dehydrogenase (*smdh*) genes in two and seven Actinobacterial MAGs, respectively. The *smdh* gene was found in seven of the ten Actinobacterial MAGs (Fig. S4). Most of the Actinobacterial MAGs are more prevalent in cryoconite, but *Iso899_MAG07g* was more abundant in soil. There is evidence of carbon monoxide (CO) oxidation via carbon monoxide dehydrogenase (*coxSML*) genes in several Acidobacteria (3/4), Actinobacteria (5/11), Alphaproteobacteria (8/15) and Gammaproteobacteria (4/8) MAGs (phylogenetic tree of coxSML genes in Figs S5–S7).

*Nitrogen:* there was only one MAG capable of nitrogen fixation from the resolved genomes. The *Nostoc_MAG71g* had *nifD* and *nifH* genes (Fig. 5), responsible for nitrogen fixation. Soil had a high number of genomes that has *nosD* and *nosZ* genes in involved in denitrification. An Alphaproteobacterial MAG, *Bradyrhizobium_MAG52g*, from the order Rhizobiales with a copy of the *napA* (nitrate reductase) gene was present in all soil and cryoconite samples. A second MAG, *Cyclobacteriaceae_MAG20f* from the Bacteroidota had a copy of the *napA* gene but was present only in the F3T3_Ludox samples. The *narG* gene (respiratory nitrate reductase) was present in two Actinobacterial MAGs, *SCTD01_MAG25f* and *Nanopelagicales_MAG08o* and two Alphaproteobacterial MAGs, *Ferrovibrionales_MAG04o* and *Pararhodobacter_MAG22g*. Actinobacterial MAGs were common in cryoconite samples, however the Proteobacterial MAGs were from the F3T3_Ludox samples.

*Sulphur:* the most common gene detected for sulphur cycling was sulphur dioxygenase (*sdo*) (phylogenetic tree in Fig. S8). Sdo oxidizes the sulphane sulphur in GSSH to sulphite, and there were 39 MAGs that had at least one of these genes. The second most abundant gene was sulphate adenylyltransferase (*sat*), gene, which forms adenosine 5'-phosphosulphate (*APS*) from ATP and free sulphate, the first step in the formation of the activated sulphate donor 3'-phosphoadenylylsulphate (*PAPS*). The gene *sat* was found in the majority of Cyanobacterial (5/6) and Chloroflexota genomes (4/5) as well as in a few Acidobacteriota (2/5), Alphaproteobacteria (2/13) and Gammaproteobacteria (1/8).

### Comparative genomic analyses of glacial cyanobacteria

**Fig. 6.**
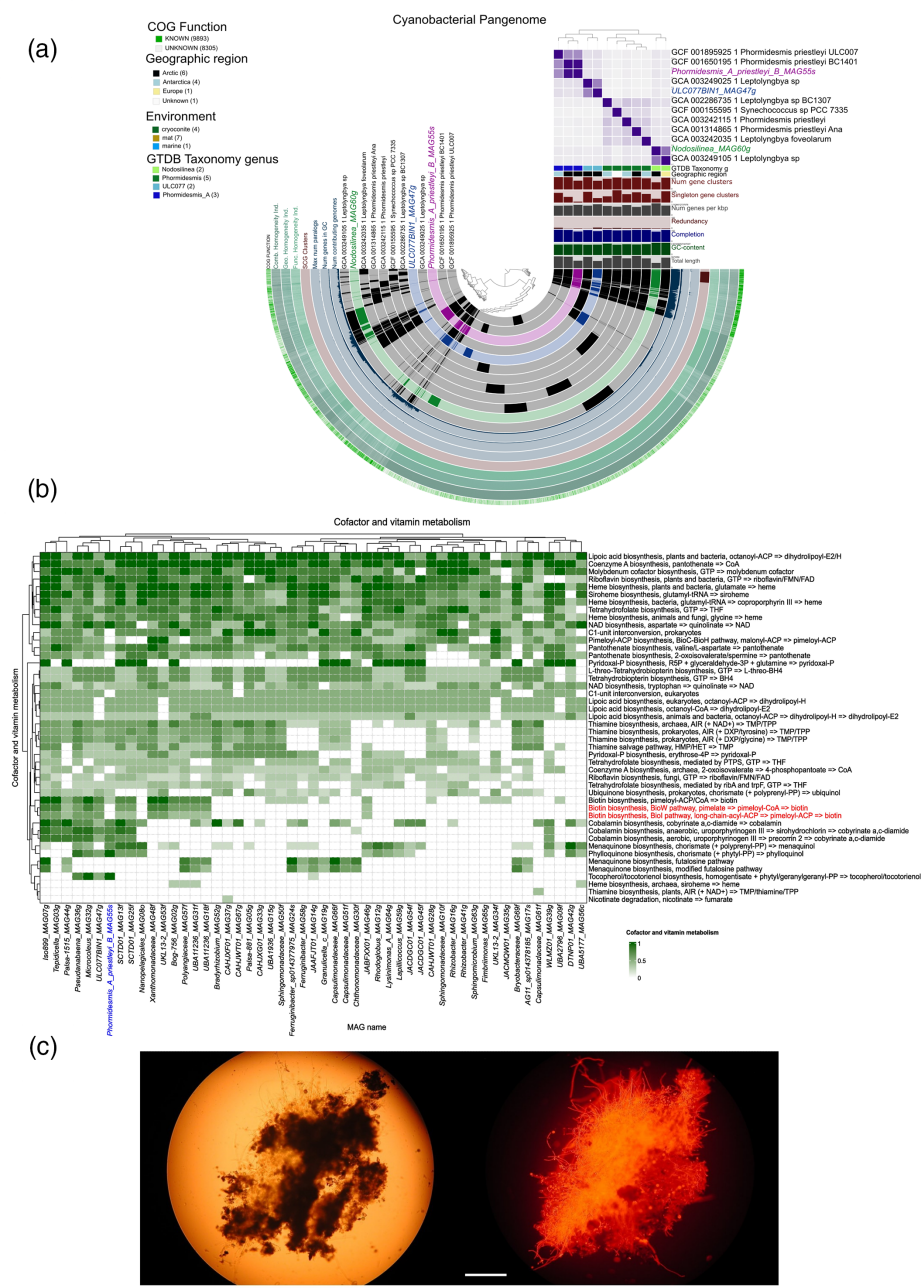
(**a**) The pangenome of *Phormidesmis* and *Leptolyngbya* species and MAGs. Public genomes (black) are compared to MAGs (colour). Regions of overlap represent common gene clusters, and non-overlapping regions represent a unique accessory genome. An ANI results matrix (purple) reflects the relatedness of the MAGs and public genomes to each other. (**b**) Comparison of the vitamin and co-factor metabolism of the cyanobacterial genomes reveals that the *Phormidesmis* Phormidesmis_A_priestleyi_B_MAG55s consistently lacks genes associated with biotin metabolism (biotin and pantothenate biosynthesis in particular). (**c**) Light (left) and chlorophyll autofluorescence (right) micrographs of a cryoconite granule from the study glaciers illustrating the ecosystem engineering through *Phormidesmis* mediated carbon fixation and granule development enabling the creation of a cohesive microhabitat which enables the transfer of soluble secondary metabolites such as biotin between members of the cryoconite granule microbiota to complement auxotrophies such as those present in *Phormidesmis* Phormidesmis_A_priestleyi_B_MAG55s (Bar=100 µm).

Cyanobacteria are notable primary producers on glacier surfaces and in forefield soils. Within cryoconite ecosystems, lineages of cyanobacteria are associated with granule formation [34]. We therefore compared five cyanobacterial MAGs: *Phormidesmis_A_priestleyi_B_MAG55s*, *ULC077BIN1_MAG47g*, *Microcoleus_MAG32g*, *Pseudanabaena_MAG36g* and *Nodosilinea_MAG60g* with genomes from closely related cyanobacteria (Fig. 6a, Tables S5 and S6). Read mapping of *Phormidesmis_A_priestleyi_B_MAG55s* was recovered at the highest coverage within our dataset, totalling 655× coverage of the genome, with 62–184× coverage of the genome in reads per cryoconite sample, and virtual absence of reads mapping to this MAG within glacier forefields (Table S11). Nevertheless, estimates for the completeness of this MAG ranged between 80–84 % (Table S10). A striking difference in metabolic potential (Figs S9–S11) for *Phormidesmis_A_priestleyi_B_MAG55s* appeared within categories associated with secondary metabolism, specifically the consistent absence of pathways required for the formation of biotin via its precursors (Figs 6b and S11). Putative auxotrophies include pimeloyl-ACP biosynthesis and conversion to biotin through three different modules for the formation of Pimeloyl-ACP, namely the BioC-BioH, BioI, and BioW pathways (KEGG: M00572, M00573, M00577). Other missing vitamin and co-factor metabolic processes include pantothenate (vitamin B5) biosynthesis and C1-unit interconversion in folate metabolism. This is at odds with other glacial cyanobacteria (Figs 6b and S11) and several non-cyanobacterial MAGs (Fig. 6b) recovered in this study from cryoconite. Cryoconite granules provide a cohesive microhabitat able to support diverse and highly active microbiota (Fig. 6c) which raises the potential for resident taxa replete in biotin biosynthesis pathways to syntrophically support *Phormidesmis_A_priestleyi_B_MAG55s*. Within the populations of MAGs present in cryoconite, MAGs which may partner with *Phormidesmis_A_priestleyi_B_MAG55s* include representatives of diverse phyla. These include members of the Cyanobacteria *Pseudanabaena_MAG36g* and *Microcoleus_MAG32g*, the Actinobacteriota *SCTD01_MAG13f* and *SCTD01_MAG25f* and *Bog-756_MAG02g* Actinobacteriota, the Betaproteobacteria *UKL13-2_MAG53f*, the candidate phylum Eremiobacterota *Palsa-1515_MAG44g* and the Bacteroidota taxon *Ferruginibacter_sp014377975_MAG24s*. Some taxa do mirror the distribution of *Phormidesmis_A_priestleyi_B_MAG55s* closely, notably *Bog-756_MAG02g* Actinobacteriota, *SCTD01_MAG25f*, *Ferruginibacter_sp014377975_MAG24s*, and *Palsa-1515_MAG44g*, although this MAG also lacks the BioW pathway for biotin biosynthesis. Therefore, the habitat type distributions of these MAGs (Fig. 4) would be consistent with the possibility for multiple supply routes of biotin given the varying abundance and distribution of the MAGs within cryoconite.

## Discussion

Metagenome-assembled genomes (MAGs) offer evidence for inferring the metabolism and genome evolution of microbes resident within complex communities across our rapidly changing planet e.g. [72], including the Arctic [74]. However, the application of genome-resolved metagenomics to elucidate the evolution and ecology of microbes within glacial ecosystems is in its relative infancy [75, 76]. Most recently, Murakami *et al*. [23] provided the first global-scale comparison of MAGs present within glaciers, revealing the range of nitrogen cycling and light harvesting mechanisms associated with cryoconite cyanobacteria.

Here we provide the first comparison of MAGs from cryoconite holes on the glacier surface with proximal but downstream terrestrial and coastal habitats. Comparisons of habitats within catchment scale [77] have particular utility. The High Arctic archipelago of Svalbard is highly glacierized, but its glacier-bearing catchments are experiencing profound change [42] and are found across pronounced gradients in temperature, precipitation and climate warming influence. Svalbard is therefore a useful barometer for changes the global scale where new terrestrial, freshwater, and marine ecosystems are displacing glaciers. Recent estimates for transition between glacier ice cover and new ecosystems (excluding the polar ice sheets of Greenland and Antarctica) for the remainder of this century in the range of 150–340 000 km^2^ [78] concomitant with the loss of 49–83 % of glaciers [79]. This represents the simultaneous loss and emergence of ecosystems at the scale of entire nations [78] but distributed in over 200 000 catchments across the world [80]. Comparing different components of the same catchment therefore offers potential insights to how the region’s microbial ecology and biogeochemistry will change as the extents of glaciers decline in the near future. Cryoconite is considered an ice-cold hotspot of microbial diversity and activity [81], and the cryoconite samples described within this study exhibited orders of magnitude more organic carbon and nitrogen compared with forefield soil from the same catchment (ML). Considering the surface loading of cryoconite on ML amounts to an estimated 4.6–10.2 tonnes per km^2^ [39], this represents a significant organic carbon and total nitrogen pool in the otherwise depauperate landscape of this deglaciating catchment.

Our study reveals distinctive MAGs present within cryoconite, capable of contributing to the biogeochemical cycling of carbon, nitrogen, sulphur and oxygen on the glacier surface. Indeed, cryoconite MAGs (Fig. 5) represented the most diverse range of biogeochemically reactive taxa in the study. Moreover, the spatial variation in MAG distribution apparent between the cryoconite communities of neighbouring glaciers is consistent with previous studies in this region [25, 38] which demonstrate glacier-specific influences on cryoconite microbiome composition. In low nutrient glacial environments, microorganisms cycle nutrients to ensure the sustained availability of a variety of nutrients. Cryoconite communities therefore play a role in geochemical nutrient cycling of major nutrients such as carbon (C) [10, 82, 83], nitrogen (N) [82, 84], and phosphorous (P) [85], as well as sulphur (S) and iron (Fe) [86]. In addition, nutrients, and bacteria from glacier surfaces are continually transported to downstream ecosystems during summer melt seasons [87]. The introduction of these nutrients to newly exposed glacial soils and the development of the microbial communities that can supplement and maintain nutrient cycling in soils is one of the most important factors driving soil development, and the eventual establishment of vegetation [17].

The most important gene in carbon fixation, responsible for the first rate-limiting step of photosynthesis, is RubisCO (Ribulose-1,5-bisphosphate carboxylase/oxygenase), which uses ribulose-1,5-bisphosphate and carbon dioxide as its substrates [88]. There are several forms of RubisCO. The form most common in autotrophic bacteria is RubisCO form I. Of these, there are several types, including the large subunit of form I red-like RubisCO (*cbbLR*), the large subunit of form I green-like RubisCO (*cbbLG*) and the large subunit of RubisCO in eukaryotes (*rbcL*). In a previous study, copies of the *cbbLR* gene from ML cryoconite most closely resembled gene sequences from an Actinobacterial genus *Mycobacterium* and Alphaproteobacteria, order Rhizobiales [82]. In this study, several MAGs had copies of form I RubisCO, including an Actinobacterial MAG, *SCTD01_MAG25f*, from the family Frankiaceae, two Alphaproteobacterial MAGs, *CAHJXG01_MAG33g* and *Bradyrhizobium_MAG52g* belonging to the family Acetobacteraceae and Xanthobacteraceae respectively as well as a Gammaproteobacterial MAG *CAHJXF01_MAG37g*, family Burkholderiaceae. *JACDBZ01_MAG70g* from the phylum Chloroflexota and *Palsa-1515_MAG44g* from the phylum Eremiobacterota also contained copies. There were also form I RubisCO genes in all the cyanobacterial MAGs, except for *Nostoc_MAG71g* and *Nodosilinea_MAG60g*. Previously amplicons of form I green-like RubisCO from *Leptolyngbya* and *Nostoc* were identified in ML cryoconite [82]. Interestingly, all MAGs harbouring RubisCO form I genes were most abundant in cryoconite, with the sole exception of *JACDBZ01_MAG70g*, which was more abundant in soil.

Glacial ecosystems in the High Arctic are sensitive to deposition of anthropogenic nitrogen from the atmosphere [89]. There was only one MAG capable of nitrogen fixation from the genomes we could resolve. The *Nostoc_MAG71g* had *nifD* and *nifH* genes (Fig. 5), responsible for nitrogen fixation. Evidence for other forms of nitrogen cycling was much clearer, consistent with marker gene studies [90]. The *napA* and *narG* genes are involved in denitrification and dissimilatory nitrate reduction. The *napA* and *narG* genes encode a periplasmic and membrane nitrate reductase respectively which reduce nitrate (NO_3_^-^) to nitrite (NO_2_^-^). Previously, *napA* and / or *narG* was amplified from all cryoconite communities from eight different glaciers on Svalbard including ML [82]. In this study, an Alphaproteobacterial MAG, *Bradyrhizobium_MAG52g* from the order Rhizobiales with a copy of the *napA* gene was present in all soil and cryoconite samples. A second MAG, *Cyclobacteriaceae_MAG20f* from the Bacteroidota had a copy of the *napA* gene but was present only in the F3T3_Ludox samples, which may not be representative of the microbial community due to extraction bias. The *narG* gene clones previously identified in Foxfonna cryoconite were most like uncultured and unclassified bacteria, as well as Rhodobacterales and Rhizobiales from the Alphaproteobacteria, and Burkholderiales from the Betaproteobacteria [82]. In this study, the *narG* gene was present in two Actinobacterial MAGs, *SCTD01_MAG25f* and *Nanopelagicales_MAG08o* and two Alphaproteobacterial MAGs, *Ferrovibrionales_MAG04o* and *Pararhodobacter_MAG22g*. Both Actinobacterial MAGs were common in cryoconite samples, however the proteobacterial MAGs were from the F3T3_Ludox samples. Sulphur cycling on glacier surfaces has not been studied widely, with the exception of a sulphur-rich spring in the Canadian Arctic which was considered exceptional [22]. Here, the genetic capacity for sulphur cycling was prevalent in the MAG collection. The most common gene for sulphur cycling was sulphur dioxygenase (*sdo*). Sdo oxidizes the sulphane sulphur in GSSH to sulphite, and there were 39 MAGs that had at least one of these genes. The sulphate adenylyltransferase (sat), gene, was detected in the majority of Cyanobacterial (5/6) and Chloroflexota genomes (4/5) as well as in a few Acidobacteria (2/5), Alphabproeobacteria (2/13) and Gammaproteobacteria (1/8). In addition, there was sulphur oxidation visa the Sulphur Oxidising (sox) pathways in several of the Alphaproteobacterial (three) and Gammaproteobacterial MAGs (six).

Focusing on cryoconite-associated cyanobacteria we found *Phormidesmis* to be highly prevalent and nearly ubiquitous within the cryoconite but at very low abundance in other habitats (Fig. 3). *Phormidesmis* was represented by a single MAG, *Phormidesmis_A_priestleyi_B_MAG55s* which could be assigned to *Phormidesmsis priestleyi* and showed high (>99.2 %) levels of ANI with *Phormidesmis priestleyi* BC1401, first isolated from Greenlandic cryoconite [32]. Our observations are consistent with a range of studies which infer the central role of filamentous cyanobacteria and in particular *Phormidesmis priestleyi* in the ecosystem engineering of granular cryoconite [24–29, 34, 35, 91] across diverse Arctic glaciers. In particular, the role of filamentous cells exuding extracellular polysaccharides (EPS) in trapping biomass, mineral dust, and other particulates is thought to play a key role in granule formation [32].

Experimental shading of solar radiation reaching *P. priestleyi*-formed Greenlandic cryoconite rapidly alters the carbon budget of the cryoconite and incurs photoadaptive responses and EPS catabolism [33]. While *P. priestleyi* may subsidize a diverse range of heterotrophic bacteria and eukaryotes [6, 38] through cryoconite formation, whether the symbioses entailed in cryoconite formation are commensal (i.e. other taxa unilaterally benefit from *P. priestleyi*) or mutualistic (i.e. *P. priestleyi* gains a fitness advantage in return) has been unexplored.

Here, the absence of genes required for essential co-factor synthesis, specifically biotin and vitamin B12 in *Phormidesmis_A_priestleyi_B_MAG55s* in spite of the high coverage obtained for this MAG implies multiple auxotrophies (Figs 6 and S10). These may be complemented by the transfer of the co-factors from taxa replete with genes for their biosynthesis co-occurring within cryoconite. A range of potential partners from diverse phyla were identified which implies that co-factors may be a common currency for occupying a niche within cryoconite. These include members of Actinobacteria, previously identified as putative metabolic keystones in Svalbard cryoconite [24].

Our treatment of this result is tentative. A limitation of MAG approaches is the potential for incomplete genomes or novel metabolic pathways to hinder complete characterisation of metabolic potential, and moreover the lack of direct evidence for co-factor exchanges. While 80–84 % completion was estimated for *Phormidesmis_A_priestleyi_B_MAG55s* overall coverage of its genome within the reads of our dataset was very high (655× in total) and virtually exclusive to cryoconite holes. Further studies are required to explore the potential for auxotrophies and strain variation within cryoconite-associated cyanobacteria. These could use labelled compound approaches to determine the provenance and extent of co-factor exchanges [92]. Nevertheless, there is clear precedent for auxotrophies of this kind and apparent cross-feeding to sustain *Phormidesmis* species within unicyanobacterial enrichment cultures from hypersaline meromictic lake microbial mats. Others have found that laboratory-maintained unicyanobacterial consortia subsidized by *Phormidesmis* species were marked by multiple auxotrophies for essential co-factors [93]. The underlying syntrophic mechanisms for metabolic exchanges of these essential co-factors represented a strategy crucial for survival within consortia through the optimization of metabolic exchanges within shared pools of micronutrients. Our observations could extend these [93] findings into the domain of naturally-occurring glacier ecosystems. Since cryoconite granules represent a cohesive microenvironment marked with closely-coupled community structure and function [25] across profound microgradients in oxygen and redox potential [94] it is likely that spatial co-location within the cryoconite granule enables direct exchanges of carbon, nutrients and essential co-factors through a range of mechanisms. These may include exudation and active transport of substances between neighbouring cells [92], but also the lytic release of materials through intense viral predation [95]. Consequently, while *Phormidesmis priestleyi* has likely engineered Arctic cryoconite ecosystems over geological timescales [96] its co-existence with other taxa in the unusual, if not unique biogeochemical milieu of cryoconite granules may have led to metabolic co-dependencies which render it vulnerable to the disruption of cryoconite ecosystems as High Arctic glaciers are lost to rapid climate warming within the region. Segawa *et al*. [35] documented the increasing fragmentation of polar glacial cyanobacterial populations since the last glacial maximum as the extents of their parent glaciers dwindled, suggesting a specialist existence for these cyanobacteria on glacier surfaces. As the Arctic continues to be warmed rapidly it is likely this trend will accelerate in the near future, potentially endangering the biodiversity of glacier specialist microbes such as *Phormidesmis priestleyi* [20].

## Supplementary Data

Supplementary material 1
