# Supplementary material for: Metagenome-assembled genomes from High Arctic glaciers highlight the vulnerability of glacier-associated microbiota and their activities to habitat loss
Source: Microb Genom. 2023 Nov 8;9(11):001131. doi: 10.1099/mgen.0.001131 (PMC10711321; doi:10.1099/mgen.0.001131)
Supplement: Supplementary material 1 [file mgen-9-1131-s001.pdf]

SUPPLEMENTARY INFORMATION

CONTENTS

1 TABLES 3

SUPPLEMENTARY TABLE 1: SAMPLE TYPE, COLLECTION DATE AND GPS COORDINATES 3
SUPPLEMENTARY TABLE 2: DNA EXTRACTION METHODS AND YIELDS 4
SUPPLEMENTARY TABLE 3: CURATED LIST OF 71 BACTERIAL SINGLE COPY GENES FOR PHYLOGENOMIC ANALYSIS: 5
SUPPLEMENTARY TABLE 4: GENE AND TIGRFAM IDS FOR BIOGEOCHEMICAL CYCLING USING METABOLISHMM 6
SUPPLEMENTARY TABLE 5 DETAILS OF REFERENCE GENOMES INCLUDED IN CYANOBACTERIAL PANGENOME. 8
SUPPLEMENTARY TABLE 6 GENOME IDS AND NUMBER OF GENES IN CYANOBACTERIAL PANGENOME 12
SUPPLEMENTARY TABLE 7 CHARACTERISTICS OF THE SVALBARD SOIL, SEAWATER AND CRYOCONITE DATASETS AFTER TRIMMING. 13
SUPPLEMENTARY TABLE 8 ASSEMBLY STATISTICS FOR THE SVALBARD METAGENOMES. 14
SUPPLEMENTARY TABLE 9 READS BASED TAXONOMIC COMPOSITION OF EACH SAMPLE BY PHYLUM AND GENUS 15
SUPPLEMENTARY TABLE 10 CHARACTERISTICS OF MAGS 22
SUPPLEMENTARY TABLE 11 TABLE SHOWING DEPTH OF COVERAGE OF EACH MAG AFTER MAPPING READS. 24
SUPPLEMENTARY TABLE 12 GTDB-TK CLASSIFICATION OF MAGS 25
SUPPLEMENTARY TABLE 13: SPECIES AND CLOSEST RELATED RELATIVE. 27
SUPPLEMENTARY TABLE 14 GEOCHEMISTRY RESULTS 29

2 SUPPLEMENTARY FIGURES 30

SUPPLEMENTARY FIGURE 1: THE SPATIAL DISTRIBUTION OF MAGS VISUALISED USING A HEATMAPS OF MAX-NORMALISED RATIO (NUMBER OF READS RECRUITED TO A CONTIG DIVIDED BY THE MAXIMUM NUMBER OF READS RECRUITED TO THAT CONTIG IN ANY SAMPLE). 31
SUPPLEMENTARY FIGURE 2: THIS FIGURE USES .HMMS FROM (ANANTHARAMAN ET AL., 2016). THIS FIGURE IS GENERATED FROM THE "SEARCH WITH HMMS OF ENVIRONMENTAL BIOELEMENT FAMILIES - V1V1.8.0" ON KBASE USING MORE PERMISSIVE HMMER THRESHOLDS THAN METABOLISHMM (FIGURE 5). THESE RESULTS CAN BE EXPLORED ON THE PUBLIC KBASE NARRATIVE: "SVALBARD-MAGS" AT HTTPS://NARRATIVE.KBASE.US/NARRATIVE/155329. 32
2.1 PHYLOGENETIC TREES 33
SUPPLEMENTARY FIGURE 3 PHYLOGENETIC TREE OF HMM HITS TO NDMA-DEPENDENT METHANOL DEHYDROGENASE (NDMA) GENE (TIGR04266). METABOLISHMM IDENTIFIED TWO ACTINOBACTERIAL MAGS WITH A NDMA GENE (MAG_059_G_LAPILLICOCCUS AND MAG_013_F_SCTD01). REFERENCE SEQUENCES IN IN BLUE BOLD. 33
SUPPLEMENTARY FIGURE 4 PHYLOGENETIC TREE OF HMM HITS TO S-(HYDROXYMETHYL)MYCOTHIOL DEHYDROGENASE (SMDH) GENE (TIGR03451). REFERENCE SEQUENCES IN IN BLUE BOLD. 33
SUPPLEMENTARY FIGURE 5 PHYLOGENETIC TREE OF HMM HITS TO CARBON MONOXIDE DEHYDROGENASE SMALL SUBUNIT GENE (COXS). REFERENCE SEQUENCES IN IN BLUE BOLD. 34
SUPPLEMENTARY FIGURE 6 PHYLOGENETIC TREE OF HMM HITS TO CARBON MONOXIDE DEHYDROGENASE MEDIUM SUBUNIT GENE (COXM). REFERENCE SEQUENCES IN IN BLUE BOLD. 35
SUPPLEMENTARY FIGURE 7 PHYLOGENETIC TREE OF HMM HITS TO CARBON MONOXIDE DEHYDROGENASE SMALL SUBUNIT GENE (COXL). REFERENCE SEQUENCES IN BLUE BOLD. 36
SUPPLEMENTARY FIGURE 8 PHYLOGENETIC TREE OF HMM HITS TO SULFUR DIOXYGENASE (SDO) GENE. (BASED ON PROTEIN SEQUENCE). REFERENCE SEQUENCE IN BLUE BOLD. 37

Supplementary Figure 9: Energy Metabolism in MAGs based on KEGG pathways (only pathways > 0.4% complete are shown) 38
Supplementary Figure 10: Central Metabolism in MAGs based on KEGG pathways (only pathways > 0.4% complete are shown) 39
Supplementary Figure 11 Cofactor and vitamin metabolism. Heatmap of cofactor and vitamin metabolism in all the MAGS. 40

3 SUPPLEMENTARY INFORMATION 41

3.1 Detailed DNA Extraction Methods 41
3.2 Supplementary Information 1: Manual refinement in anvi'o 45
Example A: Bin that is not complete across a single sample and has varying levels of coverage in different samples 46
Example B: Bin has high consensus between binning methods and consistent coverage cross a single site. 47
Example C: Bin with low consensus between binning methods, and variable coverage across contigs and across samples. 48

1 TABLES

Supplementary Table 1: Sample type, collection date and GPS coordinates

Sample site	Environment	Year	Date	Glacier	NORTH Y	EAST X	Map Key
AB_18	Cryoconite	2018	06/07/2018	Austre Brøggerbreen	78d 53' 44"	11d 49' 54"	6
VL_18	Cryoconite	2018	07/07/2018	Vestre Lovénbreen	78d 54' 14	11d 56' 27	7
VB_18	Cryoconite	2018	09/07/2018	Vestre Brøggerbreen	78d 54' 40	11d 43' 60	4
ML_18	Cryoconite	2018	11/07/2018	Midtre Lovénbreen	78d 53' 17	12d 02' 49	7
ML_17	Cryoconite	2017	12/07/2017	Midtre Lovénbreen	78d 53' 32	12d 03' 18	8
VB_17	Cryoconite	2017	10/07/2017	Vestre Brøggerbreen	78d 54' 41	11d 43' 58	5
F1T3	Soil	2017	07/07/2017	ML Forefield	78d 53' 54	12d 03' 58	11
F1T4	Soil	2017	07/07/2017	ML Forefield	78d 53' 57	12d 04' 0	10
F2T2	Soil	2017	03/07/2017	ML Forefield	78d 53' 49	12d 04' 05	14
F2T4	Soil	2017	07/07/2017	ML Forefield	78d 53' 57	12d 04' 14	12
F3T1	Soil	2017	01/07/2017	ML Forefield	78d 53' 44	12d 04' 05	15
F3T3	Soil	2017	07/07/2017	ML Forefield	78d 53' 53	12d 04' 17	13
SS1	Seawater	2017	05/07/2017	Fjord	78d 55' 27	12d 03' 59	1
SS2	Seawater	2017	05/07/2017	Fjord	78d 55' 24	12d 04' 10	2
SS3	Seawater	2017	05/07/2017	Fjord	78d 55' 22	12d 04' 53	3

Supplementary Table 2: DNA extraction methods and yields

Sample site	Sample_ID	DNA Extraction method	Starting mass (g)	DNA concentration (ng/ul)
AB_18	AB-18-04	Qiagen DNEasy PowerSoil kit (Qiagen, Inc.)	0.50	16.4
VL_18	VL-18-01	Qiagen DNEasy PowerSoil kit (Qiagen, Inc.)	0.87	41.8
VB_18	VB-18-02	Qiagen DNEasy PowerSoil kit (Qiagen, Inc.)	0.59	25.6
ML_18	ML-18-02	Qiagen DNEasy PowerSoil kit (Qiagen, Inc.)	0.58	31
ML_17	NML02	Qiagen DNEasy PowerSoil kit (Qiagen, Inc.)	0.50	21.2
VB_17	NVB03	Qiagen DNEasy PowerSoil kit (Qiagen, Inc.)	0.46	26.4
F1T3	F1T3-1	MP Biomedicals FastDNA Spin Kit for Soil (MP Biomedicals, Inc.)	0.48	2.33
F1T4	F1T4-2	MP Biomedicals FastDNA Spin Kit for Soil (MP Biomedicals, Inc.)	0.69	2.52
F2T2	F2T2-1	MP Biomedicals FastDNA Spin Kit for Soil (MP Biomedicals, Inc.)	0.59	2.7
F2T4	F2T4-2	MP Biomedicals FastDNA Spin Kit for Soil (MP Biomedicals, Inc.)	0.52	23.2
F3T1	F3T1-3	MP Biomedicals FastDNA Spin Kit for Soil (MP Biomedicals, Inc.)	0.65	1.35
F3T3_FD	F3T3-2	MP Biomedicals FastDNA Spin Kit for Soil (MP Biomedicals, Inc.)	0.52	5.28
F3T3_Lud	F3T3-1	Ludox-HS 40 + Epicentre MasterPure™ Complete DNA and RNA extraction kit	60.00	2.4
F3T3_PM	F3T3-1	MO BIO PowerMax Soil DNA Isolation Kit (MO BIO, Inc.)	10.00	19.4
SS1	SS1	Qiagen DNEasy Sterivex PowerWater kit (Qiagen, Inc.)	NA	11.2
SS2	SS2	Qiagen DNEasy Sterivex PowerWater kit (Qiagen, Inc.)	NA	10
SS3	SS2	Qiagen DNEasy Sterivex PowerWater kit (Qiagen, Inc.)	NA	10

Supplementary Table 3: Curated list of 71 bacterial single copy genes for phylogenomic analysis:

#	Gene name	#	Gene name
1	Ribosom_S12_S23	40	ADK
2	Ribosomal_L1	41	AICARFT_IMPCHas
3	Ribosomal_L13	42	ATP-synt
4	Ribosomal_L14	43	ATP-synt_A
5	Ribosomal_L16	44	Chorismate_synt
6	Ribosomal_L17	45	EF_TS
7	Ribosomal_L18p	46	Exonuc_VII_L
8	Ribosomal_L19	47	GrpE
9	Ribosomal_L2	48	Ham1p_like
10	Ribosomal_L20	49	IPPT
11	Ribosomal_L21p	50	OSCP
12	Ribosomal_L22	51	PGK
13	Ribosomal_L23	52	Pept_tRNA_hydro
14	ribosomal_L24	53	RBFA
15	Ribosomal_L27	54	RNA_pol_L
16	Ribosomal_L27A	55	RNA_pol_Rpb6
17	Ribosomal_L28	56	RRF
18	Ribosomal_L29	57	RecO_C
19	Ribosomal_L3	58	RsfS
20	Ribosomal_L32p	59	RuvX
21	Ribosomal_L35p	60	SecE
22	Ribosomal_L4	61	SecG
23	Ribosomal_L5	62	SecY
24	Ribosomal_L6	63	SmpB
25	Ribosomal_L9_C	64	TsaE
26	Ribosomal_S10	65	UPF0054
27	Ribosomal_S11	66	YajC
28	Ribosomal_S13	67	eIF-1a
29	Ribosomal_S15	68	tRNA-synt_1d
30	Ribosomal_S16	69	tRNA_m1G_MT
31	Ribosomal_S17	70	Adenylsucc_synt
32	Ribosomal_S19	71	Ribonuclease_P
33	Ribosomal_S2		
34	Ribosomal_S20p		
35	Ribosomal_S3_C		
36	Ribosomal_S6		
37	Ribosomal_S7		
38	Ribosomal_S8		
39	Ribosomal_S9		

Supplementary Table 4: Gene and TIGRFAM IDS for biogeochemical cycling using metabolisHMM

Gene Abbreviation	Protein family / TIGRFAM
	**Carbon Cycle**
nmda	ndma_methanol_dehydrogenase_TIGR04266
madA	madA_TIGR02659
madB	madB_TIGR02658
fdh	fdh_thiol_id_TIGR02819
sfh	sfh_TIGR02821
sgdh	sgdh_TIGR02818
smdh	smdh_TIGR03451
fae	fae_TIGR03126
fmtF	fmtf_TIGR03119
mtmc	mtmc_TIGR03120
fdhA	fdhA_TIGR01591
fdhB	fdhB_TIGR01582
fdhC	fdhC_TIGR01583
coxL	carbon_monoxide_dehydrogenase_coxL_TIGR02416
coxM	carbon_monoxide_dehydrogenase_coxM
coxS	carbon_monoxide_dehydrogenase_coxS
rubisco I	rubisco_form_I
rubisco I	rubisco_form_II
rubisco I	rubisco_form_III
rubisco I	rubisco_form_II_III
rubisco IV	rubisco_form_IV
codhC	codhC_TIGR00316
codhD	codhD_TIGR00381
codh cat	codh_catalytic_TIGR01702
aclA	acetate_citrate_lyase_aclA
aclB	acetate_citrate_lyase_aclB
	Nitrogen Cycle
nifD	nifD_TIGR01282
nifH	nifH_TIGR01287
nifK	nifK_TIGR01286
nxrA	nitrite_oxidoreductase_nxrA
nxrB	nitrite_oxidoreductase_nxrB
napA	napA_TIGR01706
napB	napB_PF03892
narG	narG_TIGR01580
narH	narH_TIGR01660
nrfA	nrfA_TIGR03152
nrfH	nrfH_TIGR03153
nirB	nirB_TIGR02374
nirD	nirD_TIGR02378
nirK	nirK_TIGR02376
nirS	nitrite_reductase_nirS
norB	nitric_oxide_reductase_norB
norC	nitric_oxide_reductase_norC
nosD	nosD_TIGR04247
nosZ	nosZ_TIGR04246
hzoA	hydrazine_oxidase_hzoA
hzsA	hydrazine_synthase_hzsA

Gene Abbreviation	Protein family / TIGRFAM
	Sulfur Cycle
fccB	fccB_PF09242
sqr	sulfide_quinone_oxidoreductase_sqr
sdo	sulfur_dioxygenase_sdo
aprA	aprA_TIGR02061
sat	sat_TIGR00339
dsrA	dsrA_TIGR02064
dsrB	dsrB_TIGR02066
dsrD	dsrD_PF08679
phsA	thiosulfate_reductase_phsA
soxB	soxB_TIGR04486
soxC	soxC_TIGR04555
soxY	soxY_TIGR04488
	Oxygen Cycle
coxA	coxA_TIGR02891
coxB	coxB_TIGR02866
ccoN	ccoN_TIGR00780
ccoO	ccoO_TIGR00781
ccoP	ccoP_TIGR00782
cyoA	cyoA_TIGR01433
cyoD	cyoD_TIGR02847
cyoE	cyoE_TIGR01473
cydA	cydA_PF01654
cydB	cydB_TIGR00203
qoxA	qoxA_TIGR01432
	Hydrogenases
FeFe I	FeFeHydrogenase_TIGR02512
FeFe II	FeFeHydrogenase_TIGR04105
Group I	Hydrogenase_Group_1
Group IIA	Hydrogenase_Group_2a
Group IIB	Hydrogenase_Group_2b
Group IIIA	Hydrogenase_Group_3a
Group IIIB	Hydrogenase_Group_3b
Group IIIC	Hydrogenase_Group_3c
Group IIID	Hydrogenase_Group_3d
Group IV	Hydrogenase_Group_4

HMM models without TIGRFAM accession were created by Elizabeth McDaniel and are available at https://github.com/elizabethmcd/metabolisHMM/blob/master/metabolisHMM_markers_v1.9.tgz. The metabolisHMM tool also uses trusted threshold cut-offs to identify hits.

Supplementary Table 5 Details of reference genomes included in Cyanobacterial pangenome.

ID	NCBI Organism Name	NCBI Taxonomy	GTDB Taxonomy	Location	MAG or isolate	Reference
GCA_001314865.1	Phormidesmis priestleyi Ana	p__Cyanobacteria; c__; o__Synechococcales; f__Leptolyngbyaceae; g__Phormidesmis; s__Phormidesmis priestleyi	p__Cyanobacteria; c__Cyanobacteriia; o__Phormidesmiales; f__Phormidesmiaceae; g__Phormidesmis; s__Phormidesmis priestleyi_B	Hot Lake microbial mat, Washington	MAG: derived from metagenome<br>Assembly method: IDBA_ud v. 1.1<br>Genome coverage: 490.0x<br>Sequencing technology: Illumina	(Nelson et al., 2015)
GCA_002286735.1	Leptolyngbya sp. BC1307	p__Cyanobacteria; c__; o__Synechococcales; f__Leptolyngbyaceae; g__Leptolyngbya; s__	p__Cyanobacteria; c__Cyanobacteriia; o__Phormidesmiales; f__Phormidesmiaceae; g__Phormidesmis; s__Phormidesmis sp002286735	Surface ice layer of moat surrounding Lake Hoare, McMurdo Dry Valleys, Antarctica	Isolate: Strain: BC1307<br>Assembly method: SPAdes v. 3.5<br>Expected final version: yes<br>Genome coverage: 181.0x<br>Sequencing technology: Illumina HiSeq	(Chrismas et al., 2018)
GCA_003242035.1	Leptolyngbya foveolarum	p__Cyanobacteria; c__; o__Synechococcales; f__Leptolyngbyaceae; g__Leptolyngbya; s__Leptolyngbya foveolarum	p__Cyanobacteria; c__Cyanobacteriia; o__Phormidesmiales; f__Phormidesmiaceae; g__Phormidesmis; s__Phormidesmis foveolarum	Antarctica, Transantarctic Mountains	MAG non-axenic culture: ULC129<br>Assembly method: SPAdes v. 3.10.1<br>Expected final version: yes<br>Genome coverage: 18.46x<br>Sequencing technology: Illumina MiSeq	(Cornet et al., 2018)
GCA_003242115.1	Phormidesmis priestleyi	p__Cyanobacteria; c__; o__Synechococcales; f__Leptolyngbyaceae; g__Phormidesmis; s__Phormidesmis priestleyi	p__Cyanobacteria; c__Cyanobacteriia; o__Phormidesmiales; f__Phormidesmiaceae; g__Phormidesmis; s__Phormidesmis priestleyi_A	Antarctica, Larsemann Hills	MAG non-axenic-culture: ULC027bin1<br>Assembly method: SPAdes v. 3.10.1<br>Expected final version: yes<br>Genome coverage: 6.26965x	(Cornet et al., 2018)

ID	NCBI Organism Name	NCBI Taxonomy	GTDB Taxonomy	Location	MAG or isolate	Reference
GCF_000155595.1	Synechococcus sp. PCC 7335	p__Cyanobacteria; c__; o__Synechococcales; f__Synechococcaceae; g__Synechococcus; s__	p__Cyanobacteria; c__Cyanobacteriia; o__Phormidesmiales; f__Phormidesmiaceae; g__Phormidesmis; s__Phormidesmis sp000155595	Shell (intertidal zone)	Craig Venter Institute (www.jcvi.org) sequenced, assembled, and auto-annotated the genomes	(Honda et al., 1999)
GCF_001650195.1	Phormidesmis priestleyi BC1401	p__Cyanobacteria; c__; o__Synechococcales; f__Leptolyngbyaceae; g__Phormidesmis; s__Phormidesmis priestleyi	p__Cyanobacteria; c__Cyanobacteriia; o__Leptolyngbyales; f__Leptolyngbyaceae; g__Phormidesmis_A; s__Phormidesmis_A priestleyi_B	Isolate from a cryoconite hole on the Greenland Ice Sheet	Isolate Strain: BC1401 Assembly method: SPAdes v. 3.5.0 Expected final version: no Genome coverage: 340.55x Sequencing technology: Illumina HiSeq	(Chrismas et al., 2016)
GCF_001895925.1	Phormidesmis priestleyi ULC007	p__Cyanobacteria; c__; o__Synechococcales; f__Leptolyngbyaceae; g__Phormidesmis; s__Phormidesmis priestleyi	p__Cyanobacteria; c__Cyanobacteriia; o__Leptolyngbyales; f__Leptolyngbyaceae; g__Phormidesmis_A; s__Phormidesmis_A priestleyi_A	Antarctica, Larsemann Hills	MAG non-axenic-culture: ULC007bin1 Assembly method: SPAdes v. 3.10.1 Expected final version: yes Genome coverage: 26.62x Sequencing technology: Illumina MiSeq	(Cornet et al., 2018)
GCA_003249025.1	Leptolyngbya sp.	p__Cyanobacteria; c__; o__Synechococcales; f__Leptolyngbyaceae; g__Leptolyngbya; s__	p__Cyanobacteria; c__Cyanobacteriia; o__Leptolyngbyales; f__Leptolyngbyaceae; g__ULC077BIN1; s__ULC077BIN1 sp003249025	Canada microbial mat	derived from metagenome Assembly method: SPAdes v. 3.10.1 Expected final version: yes Genome coverage: 15.08x Sequencing technology: Illumina MiSeq	(Cornet et al., 2018)
GCF_003003725.1	filamentous cyanobacterium Phorm 6	p__Cyanobacteria; c__; o__; f__; g__; s__	p__Cyanobacteria; c__Cyanobacteriia; o__Cyanobacteriales; f__Phormidiaceae; g__Microcoleus; s__Microcoleus sp003003725	Freshwater lake; Canada: Quttinirpaaq Lagoon	Isolate: Assembly method: SPAdes v. 3.11.1 Expected final version: yes Genome coverage: 6.61495x Sequencing technology: Illumina MiSeq	Unpublished: Massachusetts Institute of Technology

ID	NCBI Organism Name	NCBI Taxonomy	GTDB Taxonomy	Location	MAG or isolate	Reference
GCF_000317475.1	Oscillatoria nigro-viridis PCC 7112	p__Cyanobacteria; c__; o__Oscillatoriales; f__Oscillatoriaceae; g__Oscillatoria; s__	p__Cyanobacteria; c__Cyanobacteriia; o__Cyanobacteriales; f__Phormidiaceae; g__Microcoleus; s__Microcoleus nigroviridis	Soil in orchid house	Assembly method: Newbler v. 2.3 (pre-release), VELVET v. 1.0.13, Parallel phrap v. 4.24 SPS Genome coverage: 30x Sequencing technology: 454/Illumina	CyanoGEBA Sequencing Project
GCA_003242085.1	Pseudanabaena frigida	p__Cyanobacteria; c__; o__Synechococcales; f__Pseudanabaenaceae; g__Pseudanabaena; s__Pseudanabaena frigida	p__Cyanobacteria; c__Cyanobacteriia; o__Pseudanabaenales; f__Pseudanabaenaceae; g__Pseudanabaena; s__Pseudanabaena frigida	Canadian Arctic, Bylot Island	MAG non-axenic culture: ULC066bin1 Assembly method: SPAdes v. 3.10.1 Expected final version: yes Genome coverage: 21.86x Sequencing technology: Illumina MiSeq	(Cornet et al., 2018)
GCF_002251945.1	Pseudanabaena sp. SR411	p__Cyanobacteria; c__; o__Synechococcales; f__Pseudanabaenaceae; g__Pseudanabaena; s__	p__Cyanobacteria; c__Cyanobacteriia; o__Pseudanabaenales; f__Pseudanabaenaceae; g__Pseudanabaena; s__Pseudanabaena sp002251945	Susquehanna River, United States	Assembly method: SeqMan NG en v. 2016 Expected final version: no Genome coverage: 16.0x Sequencing technology: Illumina HiSeq	Unpublished
GCA_003249105.1	Leptolyngbya sp.	p__Cyanobacteria; c__; o__Synechococcales; f__Leptolyngbyaceae; g__Leptolyngbya; s__	p__Cyanobacteria; c__Cyanobacteriia; o__Phormidesmiales; f__Phormidesmiaceae; g__Nodosilinea; s__Nodosilinea sp003249105	Belgium, Renipont lake	MAG non-axenic culture: ULC186bin1 Assembly method: SPAdes v. 3.10.1 Expected final version: yes Genome coverage: 21.11x Sequencing technology: Illumina MiSeq	(Cornet et al., 2018)
GCF_002813575.1	Nostoc flagelliforme CCNUN1	p__Cyanobacteria; c__; o__Nostocales; f__Nostocaceae; g__Nostoc; s__Nostoc flagelliforme	p__Cyanobacteria; c__Cyanobacteriia; o__Cyanobacteriales; f__Nostocaceae; g__Nostoc; s__Nostoc flagelliforme	China: Sunitezuoqi, desert soil	Assembly method: HGAP3 v. v2.2.0 Expected final version: yes Genome coverage: 228.0x Sequencing technology: PacBio	Unpublished

Cyanobacteria public genome references

Anantharaman, K., Brown, C.T., Hug, L.A., Sharon, I., Castelle, C.J., Probst, A.J., Thomas, B.C., Singh, A., Wilkins, M.J., Karaoz, U., Brodie, E.L., Williams, K.H., Hubbard, S.S., Banfield, J.F., 2016. Thousands of microbial genomes shed light on interconnected biogeochemical processes in an aquifer system. Nat. Commun. 7, 13219. https://doi.org/10.1038/ncomms13219

Bakken, L.R., Lindahl, V., 1995. Recovery of Bacterial Cells from Soil, in: Trevors, J.T., van Elsas, J.D. (Eds.), Nucleic Acids in the Environment, Springer Lab Manuals. Springer Berlin Heidelberg, Berlin, Heidelberg, pp. 9–27. https://doi.org/10.1007/978-3-642-79050-8_2

Chrismas, N.A.M., Barker, G., Anesio, A.M., Sánchez-Baracaldo, P., 2016. Genomic mechanisms for cold tolerance and production of exopolysaccharides in the Arctic cyanobacterium Phormidesmis priestleyi BC1401. BMC Genomics 17, 533. https://doi.org/10.1186/s12864-016-2846-4

Chrismas, N.A.M., Williamson, C.J., Yallop, M.L., Anesio, A.M., Sánchez-Baracaldo, P., 2018. Photoecology of the Antarctic cyanobacterium Leptolyngbya sp. BC1307 brought to light through community analysis, comparative genomics and in vitro photophysiology. Mol. Ecol. 27, 5279–5293. https://doi.org/10.1111/mec.14953

Cornet, L., Bertrand, A.R., Hanikenne, M., Javaux, E.J., Wilmotte, A., Baurain, D., 2018. Metagenomic assembly of new (sub)polar Cyanobacteria and their associated microbiome from non-axenic cultures. Microb. Genomics 4. https://doi.org/10.1099/mgen.0.000212

Eren, A.M., Esen, Ö.C., Quince, C., Vineis, J.H., Morrison, H.G., Sogin, M.L., Delmont, T.O., 2015. Anvi'o: an advanced analysis and visualization platform for 'omics data. PeerJ 3, e1319. https://doi.org/10.7717/peerj.1319

Honda, D., Yokota, A., Sugiyama, J., 1999. Detection of Seven Major Evolutionary Lineages in Cyanobacteria Based on the 16S rRNA Gene Sequence Analysis with New Sequences of Five Marine Synechococcus Strains. J. Mol. Evol. 48, 723–739. https://doi.org/10.1007/PL00006517

McDaniel, E.A., Anantharaman, K., McMahon, K.D., 2019. metabolisHMM: Phylogenomic analysis for exploration of microbial phylogenies and metabolic pathways. bioRxiv 2019.12.20.884627. https://doi.org/10.1101/2019.12.20.884627

Nelson, W.C., Maezato, Y., Wu, Y.-W., Romine, M.F., Lindemann, S.R., 2015. Identification and Resolution of Microdiversity through Metagenomic Sequencing of Parallel Consortia. Appl. Environ. Microbiol. 82, 255–267. https://doi.org/10.1128/AEM.02274-15

Parks, D.H., Imelfort, M., Skennerton, C.T., Hugenholtz, P., Tyson, G.W., 2015. CheckM: assessing the quality of microbial genomes recovered from isolates, single cells, and metagenomes. Genome Res. 25, 1043–1055. https://doi.org/10.1101/gr.186072.114

Shaiber, A., Eren, A.M., 2019. Composite Metagenome-Assembled Genomes Reduce the Quality of Public Genome Repositories. mBio 10. https://doi.org/10.1128/mBio.00725-19

Supplementary Table 6 Genome IDs and number of genes in Cyanobacterial pangenome

Genomes	Number of genes	Partial genes
GCA_001314865_1_Phormidesmis_priestleyi_Ana	4,854	121
GCA_002286735_1_Leptolyngbya_sp__BC1307	4,459	193
GCA_003242035_1_Leptolyngbya_foveolarum	4,537	178
GCA_003242085_1_Pseudanabaena_frigida	4,862	27
GCA_003242115_1_Phormidesmis_priestleyi	4,721	558
GCA_003249025_1_Leptolyngbya_sp	5,119	289
GCA_003249105_1_Leptolyngbya_sp	4,793	433
GCF_000155595_1_Synechococcus_sp__PCC_7335	5,017	14
GCF_000317475_1_Oscillatoria_nigro_viridis_PCC7112	6,510	1
GCF_001650195_1_Phormidesmis_priestleyi_BC1401	5,253	180
GCF_001895925_1_Phormidesmis_priestleyi_ULC007	5,289	76
GCF_002251945_1_Pseudanabaena_sp_SR411	5,338	254
GCF_002813575_1_Nostoc_flagelliforme_CCNUN1	9,666	7
GCF_003003725_1_Phorm_6	5,799	603
Pseudanabaena_MAG36g	4,012	398
ULC077BIN1_MAG47g	3,718	1,005
Phormidesmis_A_priestleyi_B_MAG55s	3,639	681
Microcoleus_MAG32g	5,316	1,198
Nostoc_MAG71g	6,046	1,683
Nodosilinea_MAG60g	2,950	1,074

Supplementary Table 7 Characteristics of the Svalbard Soil, Seawater and Cryoconite datasets after trimming.

				Read Length		Duplicate reads		Quality		
**Environment**	**Sample**	**Reads**	**No Bases**	**mean**	**std dev**	**Number**	**%**	**mean**	**std dev**	**GC%**
Soil	F3T3_Lud	40,079,596	5,866,959,800	146.38	17.23	595,443	1.49	34.05	4.75	57.43
Soil	F3T3_PM	44,915,524	6,576,965,385	146.43	17.22	43,387	0.10	34.05	4.76	62.47
Soil	F3T3_FD	113,754,008	16,645,206,349	146.33	17.43	594,826	0.52	34.04	4.77	61.04
Soil	F1T3-3	37,684,166	5,477,450,552	145.35	18.97	121,309	0.32	33.89	5.01	61.17
Soil	F1T4-2	38,762,496	5,660,342,037	146.03	17.92	881,337	2.27	34.00	4.85	57.65
Soil	F2T2-1	29,116,044	4,232,776,792	145.38	19.00	310,829	1.07	33.88	5.02	56.75
Soil	F3T1-3	35,482,472	5,166,716,509	145.61	18.70	336,628	0.95	33.92	4.97	58.63
Soil	F2T4-2	35,085,040	5,124,389,299	146.06	17.92	74,333	0.21	34.00	4.83	58.69
Sea	SS1	51,330,724	7,191,372,496	140.10	25.89	1,100,183	2.14	34.20	4.54	50.69
Sea	SS2	55,019,496	7,445,759,578	135.33	32.31	1,412,286	2.57	33.89	5.02	49.25
Sea	SS3	35,985,822	4,971,626,677	138.16	29.48	775,768	2.16	33.96	4.92	49.01
Cryoconite	ML-17	28,795,356	4,194,553,943	145.67	18.57	441,991	1.53	33.89	5.02	55.16
Cryoconite	VB-17	34,504,862	5,049,762,759	146.35	17.40	601,414	1.74	34.05	4.77	54.68
Cryoconite	ML-18	28,471,672	4,149,307,242	145.73	18.46	471,631	1.66	33.94	4.93	54.46
Cryoconite	VB-18	29,787,650	4,347,438,702	145.95	18.08	384,887	1.29	33.94	4.93	55.41
Cryoconite	AB-18	64,909,032	9,494,783,816	146.28	17.54	131,932	0.20	34.02	4.81	56.75
Cryoconite	VL-18	14,939,536	2,184,519,311	146.22	17.69	78,246	0.52	34.04	4.78	54.47

Library statistics are reported after quality trimming using Trimmomatic. The untrimmed library information can be viewed in Appendix Table D-2.

Supplementary Table 8 Assembly Statistics for the Svalbard metagenomes.

Assembly	Longest contig (bp)	Nx (Lx)		Length (bp)	Num Contigs	Sum Length (bp)	Contig Length Histogram (1bp <= len < 10Kbp)	Contig Length Histogram (10Kbp <= len < 100Kbp)	Contig Length Histogram (len >= 100Kbp)
cryoconite-MEGAHIT	315846	N50:	5126	>= 10^6	0	0	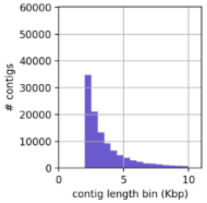	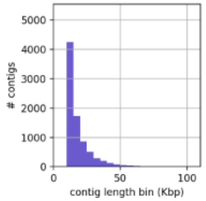	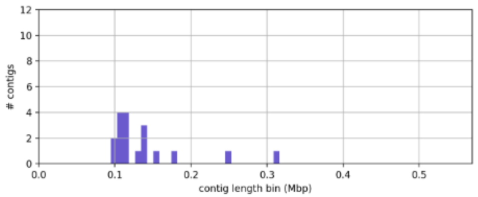
		L50:	(24906)	>= 10^5	18	2524410			
		N75:	2995	>= 10^4	8246	154707901			
		L75:	(60198)	>= 10^3	115949	538521980			
		N90:	2329	>= 500	115949	538521980			
		L90:	(90952)	>= 1	115949	538521980			
soil-MEGAHIT	300154	N50:	4111	>= 10^6	0	0	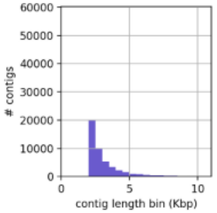	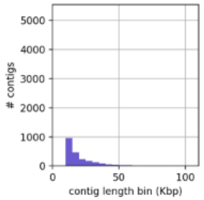	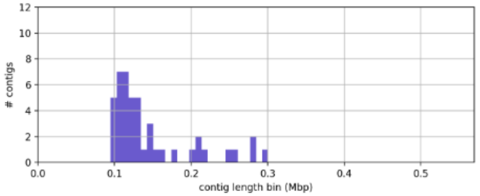
		L50:	(10133)	>= 10^5	45	6597658			
		N75:	2616	>= 10^4	2366	59047038			
		L75:	(26569)	>= 10^3	49641	208560096			
		N90:	2199	>= 500	49641	208560096			
		L90:	(39681)	>= 1	49641	208560096			
sea-MEGAHIT	115659	N50:	4236	>= 10^6	0	0	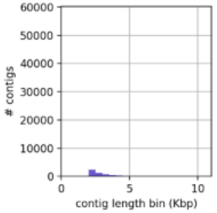	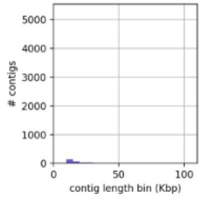	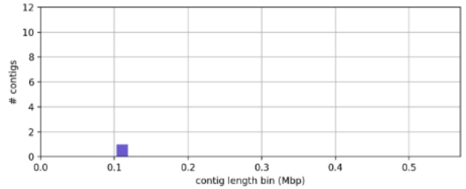
		L50:	(1450)	>= 10^5	2	221424			
		N75:	2686	>= 10^4	321	6766634			
		L75:	(3510)	>= 10^3	6451	26970110			
		N90:	2225	>= 500	6451	26970110			
		L90:	(5171)	>= 1	6451	26970110			
Svalbard MEGAHIT	554015	N50:	4739	>= 10^6	0	0	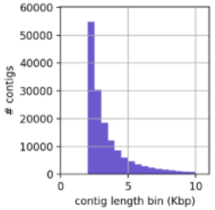	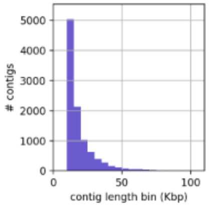	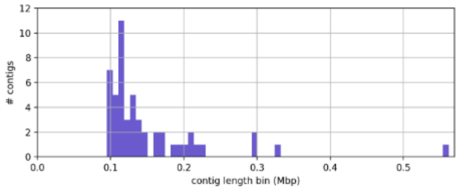
		L50:	(34565)	>= 10^5	51	7820617			
		N75:	2833	>= 10^4	10130	201062657			
		L75:	(85286)	>= 10^3	162105	720998358			
		N90:	2268	>= 500	162105	720998358			
		L90:	(128180)	>= 1	162105	720998358			

Supplementary Table 9 Reads based taxonomic composition of each sample by Phylum and Genus.

Table 9.1 Relative abundance of cryoconite microbiome by Phylum

Cryoconite																	
AB18			ML17			ML18			VB17			VB18			VL18		
Phylum	RA	Reads	Phylum	RA	Reads	Phylum	RA	Reads	Phylum	RA	Reads	Phylum	RA	Reads	Phylum	RA	Reads
Proteobacteria	43.52	792239	Cyanobacteria	39.22	313449	Cyanobacteria	39.99	308794	Cyanobacteria	39.64	382164	Proteobacteria	29.92	229180	Proteobacteria	37.11	175325
Actinobacteria	17.08	310896	Proteobacteria	21.14	168949	Proteobacteria	21.38	165093	Proteobacteria	21.46	206911	Cyanobacteria	28.58	218956	Cyanobacteria	35.21	166351
Cyanobacteria	13.55	246750	Actinobacteria	13.86	110746	Actinobacteria	15.59	120425	Actinobacteria	14.31	137977	Actinobacteria	12.75	97655	Actinobacteria	10.15	47952
Bacteroidetes	10.24	186376	Bacteroidetes	6.31	50454	Bacteroidetes	5.99	46279	Bacteroidetes	7.15	68888	Bacteroidetes	8.24	63098	Bacteroidetes	8.69	41053
Acidobacteria	2.34	42566	Chloroflexi	4.28	34175	Chloroflexi	3.06	23652	Acidobacteria	3.91	37705	Chloroflexi	3.77	28911	Chloroflexi	1.22	5776
Gemmatimonadetes	1.85	33717	Acidobacteria	4.01	32037	Acidobacteria	3.02	23331	Chloroflexi	2.84	27388	Firmicutes	3.31	25385	Gemmatimonadetes	1.08	5090
Firmicutes	1.70	30985	Firmicutes	2.80	22403	Firmicutes	2.34	18109	Firmicutes	2.53	24368	Acidobacteria	3.26	24938	Firmicutes	0.88	4143
Chloroflexi	1.61	29280	Armatimonadetes	0.72	5781	Ascomycota	0.82	6307	Ascomycota	0.84	8098	Planctomycetes	1.03	7881	Verrucomicrobia	0.81	3804
Planctomycetes	1.13	20500	Ascomycota	0.67	5370	Planctomycetes	0.66	5078	Planctomycetes	0.68	6561	Armatimonadetes	0.80	6118	Planctomycetes	0.74	3503
Verrucomicrobia	0.71	12872	Planctomycetes	0.64	5116	Armatimonadetes	0.61	4685	Deinococcus-Thermus	0.60	5790	Gemmatimonadetes	0.70	5386	Acidobacteria	0.62	2937
Chlorophyta	0.58	10486	Deinococcus-Thermus	0.57	4529	Deinococcus-Thermus	0.59	4528	Armatimonadetes	0.57	5454	Ascomycota	0.62	4745	Viruses	0.04	171
Ascomycota	0.55	9922	Viruses	0.06	515	Viruses	0.08	632	Viruses	0.08	743	Viruses	0.10	776	unclassified	1.71	8060
Armatimonadetes	0.53	9565	unclassified	2.22	17771	unclassified	2.23	17196	unclassified	2.09	20112	unclassified	2.58	19748	Rare (<0.5%)	1.75	8280
Viruses	0.05	945	Rare (<0.5%)	3.50	28002	Rare (<0.5%)	3.64	28143	Rare (<0.5%)	3.32	31978	Rare (<0.5%)	4.35	33296			
unclassified	2.09	38051															
Rare (<0.5%)	2.49	45265															

RA: Relative abundance

Rare: X_Phylum with < 0.5% of all reads

Unassigned X_not assigned to a Phylum

Table 9.2 Relative abundance of seawater microbiome by Phylum

Sea								
SS1			SS2			SS3		
Phylum	RA	Reads	Phylum	RA	Reads	Phylum	RA	Reads
Proteobacteria	36.56	436936	Proteobacteria	42.48	128772	Proteobacteria	41.00	82189
Firmicutes	28.80	344180	Bacteroidetes	19.25	58367	Bacteroidetes	22.36	44822
Actinobacteria	18.00	215057	Chlorophyta	5.34	16203	Chlorophyta	5.94	11906
Euryarchaeota	2.95	35235	Bacillariophyta	4.02	12199	Bacillariophyta	4.11	8248
Bacteroidetes	1.13	13546	Actinobacteria	1.37	4158	Firmicutes	1.31	2634
Crenarchaeota	0.86	10231	Firmicutes	1.24	3772	Actinobacteria	1.23	2463
Chlorophyta	0.66	7831	Ascomycota	1.19	3608	Ascomycota	1.20	2397
Viruses	0.38	4513	Apicomplexa	1.16	3514	Euglenida	1.07	2148
unclassified	9.37	111975	Euglenida	0.96	2912	Apicomplexa	0.88	1758
Rare (<0.5%)	1.30	15546	Verrucomicrobia	0.64	1947	Basidiomycota	0.75	1498
			Basidiomycota	0.64	1937	Verrucomicrobia	0.56	1115
			Viruses	6.53	19811	Cyanobacteria	0.51	1031
			unclassified	12.41	37615	Viruses	3.96	7945
			Rare (<0.5%)	2.75	8349	unclassified	12.79	25642
						Rare (<0.5%)	2.33	4671

RA: Relative abundance
Rare: X_Phylum with < 0.5% of all reads
Unassigned X_not assigned to a Phylum

Table 9.3 Relative abundance of soil microbiome by Phylum

Soil											
F1T3			F1T4			F2T2			F2T4		
Phylum	RA	Reads	Phylum	RA	Reads	Phylum	RA	Reads	Phylum	RA	Reads
Proteobacteria	29.98	340400	Actinobacteria	25.52	273961	Proteobacteria	30.59	268533	Proteobacteria	34.72	337528
Actinobacteria	27.50	312222	Proteobacteria	23.96	257121	Cyanobacteria	18.85	165453	Actinobacteria	19.17	186351
Bacteroidetes	7.84	89017	Bacteroidetes	15.44	165736	Actinobacteria	17.50	153641	Bacteroidetes	9.49	92242
Acidobacteria	7.10	80648	Acidobacteria	7.30	78315	Bacteroidetes	9.50	83389	Acidobacteria	7.01	68186
Cyanobacteria	5.08	57635	Chloroflexi	5.28	56711	Acidobacteria	4.45	39089	Planctomycetes	5.19	50494
Chloroflexi	4.07	46221	Cyanobacteria	4.02	43118	Planctomycetes	3.54	31086	Chloroflexi	4.77	46360
Planctomycetes	3.45	39230	Planctomycetes	3.52	37774	Chloroflexi	3.14	27525	Cyanobacteria	4.76	46280
Firmicutes	2.74	31106	Verrucomicrobia	2.10	22533	Ascomycota	2.52	22140	Verrucomicrobia	3.07	29864
Verrucomicrobia	2.03	23056	Gemmatimonadetes	1.86	19994	Verrucomicrobia	2.23	19583	Gemmatimonadetes	1.51	14708
Gemmatimonadetes	1.72	19538	Firmicutes	1.59	17094	Firmicutes	0.96	8384	Ascomycota	1.50	14594
Ascomycota	1.03	11746	Nitrospirae	1.08	11591	Gemmatimonadetes	0.82	7228	Firmicutes	1.31	12702
Nitrospirae	0.61	6876	Ascomycota	0.80	8625	Nitrospirae	0.59	5169	Viruses	0.05	452
Viruses	0.21	2344	Viruses	0.05	510	Viruses	0.03	266	unclassified	3.18	30890
unclassified	3.27	37117	unclassified	3.40	36544	unclassified	2.40	21064	Rare (<0.5%)	4.26	41363
Rare (<0.5%)	3.38	38336	Rare (<0.5%)	4.07	43723	Rare (<0.5%)	2.87	25174			

Soil											
F3T1			F3T3_FD			F3T3_Lud			F3T3_PM		
Phylum	RA	Reads	Phylum	RA	Reads	Phylum	RA	Reads	Phylum	RA	Reads
Proteobacteria	38.10	415457	Proteobacteria	30.40	999159	Proteobacteria	43.74	558259	Proteobacteria	40.40	557022
Actinobacteria	23.51	256394	Actinobacteria	27.82	914299	Bacteroidetes	25.12	320639	Actinobacteria	22.97	316670
Bacteroidetes	12.12	132165	Bacteroidetes	9.14	300358	Actinobacteria	10.38	132516	Bacteroidetes	8.22	113360
Cyanobacteria	5.17	56361	Acidobacteria	7.31	240406	Acidobacteria	5.22	66651	Acidobacteria	6.96	95971
Chloroflexi	3.97	43293	Planctomycetes	4.09	134293	Planctomycetes	2.97	37892	Chloroflexi	2.97	40920
Acidobacteria	3.49	38080	Chloroflexi	4.04	132889	Chloroflexi	2.01	25688	Cyanobacteria	2.83	39011
Planctomycetes	2.83	30847	Cyanobacteria	3.67	120663	Firmicutes	1.56	19945	Planctomycetes	2.82	38812
Verrucomicrobia	2.43	26477	Verrucomicrobia	2.41	79234	Verrucomicrobia	1.09	13966	Gemmatimonadetes	2.36	32476
Firmicutes	0.96	10500	Gemmatimonadetes	1.46	47899	Cyanobacteria	0.77	9791	Verrucomicrobia	2.33	32108
Gemmatimonadetes	0.95	10409	Firmicutes	1.33	43726	Gemmatimonadetes	0.65	8272	Firmicutes	1.13	15554
Ascomycota	0.59	6429	Ascomycota	0.99	32485	Viruses	0.02	257	Ascomycota	0.71	9798
Viruses	0.08	864	Nitrospirae	0.68	22476	unclassified	3.05	38956	Nitrospirae	0.50	6925
unclassified	2.59	28294	Viruses	0.03	1008	Rare (<0.5%)	3.40	43365	Viruses	0.02	298
Rare (<0.5%)	3.21	35007	unclassified	3.20	105227				unclassified	3.01	41543
			Rare (<0.5%)	3.43	112596				Rare (<0.5%)	2.77	38255

RA: Relative abundance
Rare: X_Phylum with < 0.5% of all reads
Unassigned X_not assigned to a Phylum

Table 9.4 Relative abundance of cryoconite microbiome by Genus

Cryoconite																	
AB18			ML17			ML18			VB17			VB18			VL18		
Genus	RA	Reads	Genus	RA	Reads	Genus	RA	Reads	Genus	RA	Reads	Genus	RA	Reads	Genus	RA	Reads
Rare (<0.5%)	46.84	852758	Rare (<0.5%)	35.75	285732	Rare (<0.5%)	35.60	274904	Rare (<0.5%)	36.40	350960	Rare (<0.5%)	42.48	325408	Rare (<0.5%)	41.76	197275
Unassigned	24.75	450600	Phormidesmis	33.92	271134	Phormidesmis	33.81	261136	Phormidesmis	33.69	324833	Phormidesmis	24.12	184759	Unassigned	24.51	115794
Phormidesmis	6.23	113358	Unassigned	18.22	145616	Unassigned	18.21	140594	Unassigned	18.58	179099	Unassigned	22.25	170430	Nostoc	7.28	34373
Sphingomonas	2.45	44584	Ktedonobacter	2.33	18660	Ktedonobacter	1.57	12129	Sphingomonas	1.42	13677	Ktedonobacter	1.82	13963	Phormidesmis	6.00	28365
Polaromonas	2.27	41385	Granulicella	1.16	9289	Bradyrhizobium	1.04	8012	Ktedonobacter	1.37	13240	Sphingomonas	1.32	10139	Polaromonas	2.92	13811
Bradyrhizobium	2.16	39401	Sphingomonas	1.13	9010	Sphingomonas	1.03	7980	Granulicella	1.13	10892	Bradyrhizobium	1.17	8972	Leptolyngbya	1.98	9347
Variovorax	1.19	21712	Mucilaginibacter	1.03	8215	Streptomyces	0.99	7630	Leptolyngbya	0.98	9471	Streptomyces	1.02	7833	Oscillatoria	1.56	7361
Oscillatoria	1.14	20704	Streptomyces	0.97	7771	Pseudanabaena	0.97	7528	Streptomyces	0.96	9248	Mucilaginibacter	0.84	6459	Sphingomonas	1.43	6766
Streptomyces	1.12	20298	Bradyrhizobium	0.91	7236	Leptolyngbya	0.87	6756	Hymenobacter	0.93	8960	Labilithrix	0.80	6165	Microcoleus	1.41	6644
Rhizobacter	1.04	18972	Leptolyngbya	0.88	6995	Granulicella	0.82	6335	Mucilaginibacter	0.92	8822	Leptolyngbya	0.68	5207	Tolypothrix	0.80	3762
Microcoleus	0.99	18023	Hymenobacter	0.80	6393	Leifsonia	0.73	5674	Bradyrhizobium	0.70	6738	Polaromonas	0.60	4588	Flavobacterium	0.79	3754
Gemmatimonas	0.94	17192	Leifsonia	0.61	4841	Mucilaginibacter	0.72	5530	Novosphingobiun	0.67	6417	Granulicella	0.58	4436	Methylibium	0.79	3749
Cryobacterium	0.82	14889	Novosphingobium	0.60	4832	Microbacterium	0.70	5385	Leifsonia	0.58	5637	Variovorax	0.57	4355	Variovorax	0.78	3679
Methylibium	0.76	13799	Microbacterium	0.59	4743	Cryobacterium	0.68	5273	Microbacterium	0.55	5338	Hymenobacter	0.56	4308	Nocardioides	0.78	3678
Ktedonobacter	0.69	12649	Cryobacterium	0.53	4201	Hymenobacter	0.62	4771	Cryobacterium	0.52	5036	Novosphingobium	0.56	4303	Rhizobacter	0.76	3614
Leifsonia	0.68	12415	Thermogemmatispor	0.51	4114	Novosphingobium	0.54	4146	Deinococcus	0.52	5026	Mycobacterium	0.52	3972	Calothrix	0.75	3555
Flavobacterium	0.68	12358	Viruses	0.06	515	Mycobacterium	0.51	3919	Viruses	0.08	743	Viruses	0.10	776	Chamaesiphon	0.70	3293
Microbacterium	0.63	11540				Deinococcus	0.51	3918							Gemmatimonas	0.68	3208
Rhodoferax	0.63	11442				Viruses	0.08	632							Ilumatobacter	0.67	3176
Hymenobacter	0.61	11142													Streptomyces	0.65	3058
Novosphingobium	0.58	10648													Rhodoferax	0.62	2920
Mycobacterium	0.58	10489													Hydrogenophaga	0.60	2848
Pseudanabaena	0.56	10133													Hymenobacter	0.59	2800
Pedobacter	0.55	10021													Anabaena	0.59	2768
Pseudomonas	0.53	9674													Scytonema	0.57	2676
Nostoc	0.51	9284													Viruses	0.04	171
Viruses	0.05	945															

RA: Relative abundance
Rare: X_genus with < 0.5% of all reads
Unassigned X_not assigned to a genus

Table 9.5 Relative abundance of seawater microbiome by Genus

Sea								
SS1			SS2			SS3		
Genus	RA	Reads	Genus	RA	Reads	Genus	RA	Reads
Ralstonia	28.24	337443	Rare (<0.5%)	36.20	109754	Rare (<0.5%)	38.35	76889
Clostridioides	23.17	276836	Unassigned	29.20	88537	Unassigned	30.30	60738
Mycobacterium	17.10	204408	Candidatus Pelagibacter	8.68	26313	Candidatus Pelagibacter	6.84	13709
Unassigned	12.26	146492	Viruses	6.53	19811	Viruses	3.96	7945
Rare (<0.5%)	7.01	83830	Micromonas	3.62	10973	Micromonas	3.36	6738
Clostridium	4.89	58401	Thalassiosira	3.35	10154	Thalassiosira	3.23	6477
Methanosarcina	2.93	35065	Symbiodinium	2.06	6253	Symbiodinium	1.97	3950
Campylobacter	1.84	21977	Polaribacter	1.76	5348	Polaribacter	1.84	3694
Sulfolobus	0.86	10221	Ulvibacter	1.19	3611	Ulvibacter	1.46	2918
Pseudomonas	0.71	8502	Formosa	1.06	3211	Formosa	1.29	2592
Streptomyces	0.62	7362	Aequorivita	1.03	3134	Aequorivita	1.25	2504
Viruses	0.38	4513	Flavobacterium	0.94	2852	Bathycoccus	1.22	2453
			Sulfitobacter	0.78	2351	Flavobacterium	0.93	1862
			Eutreptiella	0.75	2284	Eutreptiella	0.85	1707
			Pseudomonas	0.64	1937	Sulfitobacter	0.82	1644
			Bathycoccus	0.60	1831	Pseudomonas	0.67	1346
			Plasmodium	0.55	1663	Altibacter	0.60	1210
			Methylophaga	0.52	1582	Aquimarina	0.54	1078
			Candidatus Thioglobus	0.52	1565	Candidatus Thioglobus	0.51	1013

RA: Relative abundance

Rare: X_genus with < 0.5% of all reads

Unassigned X_not assigned to a genus

Table 9.6 Relative abundance of soil microbiome by Genus

Soil											
F1T3			F1T4			F2T2			F2T4		
Genus	RA	Reads	Genus	RA	Reads	Genus	RA	Reads	Genus	RA	Reads
Unassigned	44.45	504744	Unassigned	48.53	520844	Rare (<0.5%)	42.12	369717	Unassigned	44.93	436732
Rare (<0.5%)	39.36	446960	Rare (<0.5%)	40.30	432613	Unassigned	37.76	331447	Rare (<0.5%)	42.95	417466
Nocardioides	2.44	27736	Nocardioides	2.81	30161	Nostoc	7.76	68153	Sphingomonas	1.84	17914
Sphingomonas	2.39	27179	Sphingomonas	1.59	17090	Sphingomonas	3.26	28603	Nocardioides	1.30	12621
Streptomyces	1.47	16747	Streptomyces	1.47	15818	Leptolyngbya	2.61	22875	Streptomyces	1.12	10846
Clostridioides	1.32	14996	Spirosoma	0.82	8806	Nocardioides	2.45	21464	Bradyrhizobium	0.98	9554
Nostoc	1.27	14411	Nitrospira	0.73	7812	Streptomyces	0.87	7641	Gemmata	0.93	9040
Conexibacter	0.90	10224	Hymenobacter	0.68	7260	Endocarpon	0.85	7456	Leptolyngbya	0.91	8880
Solirubrobacter	0.85	9625	Bradyrhizobium	0.67	7170	Spirosoma	0.64	5614	Nostoc	0.83	8041
Agrobacterium	0.84	9485	Conexibacter	0.62	6671	Gemmata	0.60	5242	Mesorhizobium	0.79	7685
Bradyrhizobium	0.79	8932	Flavisolibacter	0.61	6565	Calothrix	0.53	4641	Polaromonas	0.61	5919
Pseudonocardia	0.70	7921	Solirubrobacter	0.59	6349	Bradyrhizobium	0.52	4605	Pseudonocardia	0.60	5831
Leptolyngbya	0.66	7508	Pedobacter	0.53	5681	Viruses	0.03	266	Phycicoccus	0.58	5630
Spirosoma	0.63	7110	Viruses	0.05	510				Rhodomicrobium	0.57	5546
Gemmata	0.60	6843							Variovorax	0.51	4965
Hymenobacter	0.58	6596							Hyphomicrobium	0.50	4892
Mesorhizobium	0.54	6131							Viruses	0.05	452
Viruses	0.21	2344									

RA: Relative abundance

Rare: X_genus with < 0.5% of all reads

Unassigned X_not assigned to a genus

Soil											
F3T1			F3T3_FD			F3T3_Lud			F3T3_PM		
Genus	RA	Reads	Genus	RA	Reads	Genus	RA	Reads	Genus	RA	Reads
Unassigned	40.32	439724	Unassigned	45.86	1507239	Rare (<0.5%)	40.69	519250	Unassigned	43.46	599145
Rare (<0.5%)	38.85	423715	Rare (<0.5%)	41.77	1372804	Unassigned	38.93	496776	Rare (<0.5%)	38.95	536945
Nocardioides	6.96	75882	Nocardioides	2.84	93257	Cecembia	4.52	57747	Sphingomonas	8.22	113362
Sphingomonas	2.52	27474	Sphingomonas	2.74	90045	Sphingomonas	4.02	51299	Nocardioides	2.64	36465
Leptolyngbya	1.18	12879	Streptomyces	1.48	48771	Aquiflexum	2.42	30883	Streptomyces	1.25	17229
Cryobacterium	0.95	10335	Conexibacter	0.88	28853	Tepidicella	1.93	24671	Sphingosinicella	0.80	11006
Streptomyces	0.90	9811	Solirubrobacter	0.86	28313	Sporocytophaga	1.73	22051	Conexibacter	0.71	9760
Pedobacter	0.89	9702	Bradyrhizobium	0.67	22022	Brevundimonas	0.78	9999	Solirubrobacter	0.67	9219
Flavobacterium	0.79	8657	Spirosoma	0.67	21941	Microcella	0.75	9528	Brevundimonas	0.61	8361
Nostoc	0.76	8239	Mesorhizobium	0.65	21413	Nocardioides	0.69	8797	Hymenobacter	0.61	8356
Arthrobacter	0.73	7911	Pseudonocardia	0.53	17373	Sphingosinicella	0.69	8749	Actinoplanes	0.55	7628
Devosia	0.70	7681	Gemmata	0.52	17152	Algoriphagus	0.64	8144	Novosphingobium	0.52	7108
Bradyrhizobium	0.68	7458	Actinoplanes	0.50	16527	Mesorhizobium	0.63	8087	Bradyrhizobium	0.50	6928
Mesorhizobium	0.67	7272	Viruses	0.03	1008	Natronohydrobacter	0.55	6997	Mesorhizobium	0.50	6913
Altererythrobacter	0.65	7056				Streptomyces	0.51	6492	Viruses	0.02	298
Polaromonas	0.60	6563				Bradyrhizobium	0.51	6470			
Thiobacillus	0.60	6531				Viruses	0.02	257			
Novosphingobium	0.59	6440									
Hymenobacter	0.59	6383									
Viruses	0.08	864									

RA: Relative abundance

Rare: X_genus with < 0.5% of all reads

Unassigned X_not assigned to a genus

Supplementary Table 10 Characteristics of MAGs

						HMM		CheckM											
MAG	Kaiju_taxon	total length	num contigs	N50	GC content	completion (%)	redundancy (%)	Marker Lineage	# Genomes	# Markers	# Marker Sets	0	1	2	3	4	5+	Comple-tion	Conta-mination
UBA5704_MAG01f	Unknown	3997738	152	39686	69.07	100.00	1.41	k__Bacteria	2258	188	117	3	183	2	0	0	0	98.29	1.71
Bog-756_MAG02g	Unknown	3194344	178	29706	65.93	100.00	1.41	k__Bacteria	901	171	117	8	159	3	1	0	0	93.92	2.99
Tepidicella_MAG03g	Hydrogenophaga	3001625	147	29296	65.34	100.00	2.82	o__Burkholderiales	193	427	214	10	413	4	0	0	0	97.78	1.43
Ferrovibrionales_MAG04o	Unknown	4947466	409	16841	68.99	98.59	1.41	o__Rhodospirillales	63	336	201	6	327	3	0	0	0	97.51	1
Palsa-881_MAG05g	Caulobacter	3232581	248	20887	64.11	98.59	4.23	c__Alphaproteobacteria	26	529	308	40	484	5	0	0	0	92.16	1.62
Cytophagaceae_MAG06f	Cytophaga	3673287	48	136173	42.04	97.18	0.00	o__Cytophagales	47	454	336	2	448	3	1	0	0	99.4	1.49
Iso899_MAG07g	Unknown	4713114	185	33034	69.61	97.18	1.41	o__Actinomycetales	274	388	214	19	352	17	0	0	0	94.19	4.26
Nanopelagicales_MAG08o	Unknown	3827765	339	16209	64.22	97.18	1.41	o__Actinomycetales	455	315	190	12	299	4	0	0	0	95.84	1.84
UBA2796_MAG09f	Unknown	5637460	593	12640	54.47	97.18	2.82	k__Bacteria	924	163	110	5	155	3	0	0	0	98.69	2.73
Sphingomonadaceae_MAG10f	Unknown	2931628	291	13543	65.14	97.18	2.82	c__Alphaproteobacteria	564	349	230	22	325	2	0	0	0	91.65	0.65
Allosphingosinicella_MAG11g	Sphingosinicella	3477485	118	42505	66.66	97.18	4.23	c__Alphaproteobacteria	564	349	230	16	330	3	0	0	0	95.33	0.69
Rhodoglobus_MAG12g	Salinibacterium	2581198	166	23432	62.93	97.18	4.23	o__Actinomycetales	69	400	198	9	381	10	0	0	0	97.34	2.05
SCTD01_MAG13f	Unknown	3574416	307	23221	71.23	97.18	7.04	o__Actinomycetales	488	309	185	7	290	12	0	0	0	97.03	3.24
JAAFJT01_MAG14g	Unknown	4081280	530	9503	36.55	95.77	1.41	p__Bacteroidetes	364	302	202	24	271	7	0	0	0	93.11	2.42
UBA1936_MAG15g	Sphingomonas	3222436	215	27127	60.08	95.77	1.41	c__Alphaproteobacteria	564	349	230	5	342	2	0	0	0	98.26	0.87
Rhizobacter_MAG16g	Unknown	4051397	233	26619	68.71	95.77	2.82	o__Burkholderiales	193	427	214	14	397	14	2	0	0	97.62	4.44
AG11_sp014378185_MAG17s	Gemmatirosa	3429033	213	24246	65.44	95.77	2.82	k__Bacteria	2993	147	91	18	128	1	0	0	0	89.97	1.1
UBA11236_MAG18f	Unknown	3683642	435	12732	42.97	95.77	5.63	k__Bacteria	2993	147	91	11	130	5	1	0	0	90.91	3.08
Granulicellac_MAG19g	Granulicella	3773816	296	17675	58.01	95.77	7.04	k__Bacteria	2258	188	117	13	173	2	0	0	0	90.54	1.28
Cyclobacteriaceae_MAG20f	Unknown	4845095	302	22910	38.78	94.37	0.00	o__Cytophagales	47	454	336	6	444	4	0	0	0	98.21	1.19
Lacisediminihabitans_MAG21g	Unknown	3151193	231	19819	64.26	94.37	0.00	o__Actinomycetales	69	400	198	29	358	12	1	0	0	94.64	4.73
Pararhodobacter_MAG22g	Unknown	3720906	603	7696	70.98	94.37	2.82	f__Rhodobacteraceae	84	568	330	81	484	3	0	0	0	83.3	0.67
ASP10-02a_sp002335115_MAG23s	Unknown	2563445	129	32893	45.24	94.37	2.82	o__Oceanospirillales	43	529	233	38	487	4	0	0	0	93.71	0.75
Ferruginibacter_sp014377975_MAG24s	Unknown	4943213	471	15645	37.88	94.37	4.23	p__Bacteroidetes	364	303	203	21	272	10	0	0	0	96.22	3.86
SCTD01_MAG25f	Unknown	3532430	250	17768	68.78	94.37	4.23	o__Actinomycetales	488	309	185	31	272	6	0	0	0	87.03	2.78
UBA3362_MAG26g	Unknown	3384069	404	11587	35.24	94.37	4.23	p__Bacteroidetes	364	303	203	15	284	4	0	0	0	93.43	0.66
UBA11741_MAG27g	Unknown	4420110	372	16213	54.65	94.37	5.63	k__Bacteria	2258	188	117	12	171	5	0	0	0	92.47	3.59
CAHJWT01_MAG28g	Sphingomonas	2904960	437	7986	65.46	94.37	5.63	c__Alphaproteobacteria	564	349	230	47	283	19	0	0	0	88.64	5.44
UBA11400_MAG29g	Candidatus Cyclonatronum	2523900	60	69398	45.16	92.96	0.00	k__Bacteria	433	274	183	18	254	2	0	0	0	90.44	0.82
Chthonomonadaceae_MAG30f	Unknown	5120342	663	9491	56.41	92.96	4.23	k__Bacteria	924	161	108	9	150	2	0	0	0	92.59	1.85
UBA11236_MAG31f	Unknown	3923198	439	13667	43.19	92.96	5.63	k__Bacteria	2993	147	91	6	136	4	1	0	0	94.44	6.04
Microcoleus_MAG32g	Oscillatoria	5457007	948	6866	45.41	92.96	7.04	p__Cyanobacteria	79	583	457	56	502	25	0	0	0	89.17	4.6
CAHJXG01_MAG33g	Unknown	3340025	391	10992	65.58	92.96	8.45	o__Rhodospirillales	63	336	201	30	301	5	0	0	0	88.47	1.18
UKL13-2_MAG34f	Unknown	2402288	476	5405	51.80	91.55	1.41	c__Betaproteobacteria	235	420	211	81	331	8	0	0	0	76.04	2.34
JACMQW01_MAG35g	Unknown	2850025	287	13776	56.12	91.55	2.82	k__Bacteria	2258	182	112	13	168	1	0	0	0	88.84	0.89
Pseudanabaena_MAG36g	Pseudanabaena	3926578	347	16026	41.06	91.55	4.23	p__Cyanobacteria	89	544	424	31	501	12	0	0	0	94.44	2.24

MAG	Kaiju_taxon	total length	num contigs	N50	GC content	HMM completion (%)	HMM redundancy (%)	CheckM Marker Lineage	# Genomes	# Markers	# Marker Sets	0	1	2	3	4	5+	Comple-tion	Conta-mination
CAHJXF01_MAG37g	Unknown	4187073	330	18017	68.21	91.55	5.63	c__Betaproteobacteria	323	387	234	49	329	9	0	0	0	89.87	2.28
UBA10364_MAG38g	Unknown	1841681	133	18813	41.70	90.14	0.00	k__Bacteria	434	278	186	15	261	2	0	0	0	93.55	0.56
WLMZ01_MAG39g	Unknown	5220682	511	15028	64.26	90.14	1.41	k__Bacteria	901	171	117	13	153	2	2	1	0	91.95	5.56
UBA6161_MAG40g	Unknown	3254196	446	8589	36.52	90.14	4.23	p__Bacteroidetes	350	316	210	47	265	4	0	0	0	82.59	1.13
Rhizobacter_MAG41g	Rhizobacter	3284861	228	21863	68.04	90.14	9.86	o__Burkholderiales	193	427	214	37	374	16	0	0	0	89.72	2.46
DTNP01_MAG42g	Unknown_Ktedonobacteria	4685611	848	6700	53.20	88.73	1.41	k__Bacteria	924	151	101	11	133	7	0	0	0	93.05	4.73
AC-14_MAG43f	Unknown	2225260	392	6786	66.48	87.32	1.41	k__Bacteria	901	171	117	31	137	3	0	0	0	82.48	2.14
Palsa-1515_MAG44g	Unknown	2744076	427	8453	62.42	87.32	2.82	k__Bacteria	924	163	110	22	138	3	0	0	0	89.23	1.16
JACDGC01_MAG45f	Unknown_Ktedonobacteria	3183126	474	7727	47.61	87.32	4.23	k__Bacteria	924	151	101	25	124	2	0	0	0	81.71	1.98
JABFXX01_MAG46g	Haliangium	6278991	987	7373	66.04	87.32	9.86	c__Deltaproteobacteria	83	247	155	44	197	6	0	0	0	82.42	2.1
ULC077BIN1_MAG47g	Unknown	3491281	719	5103	48.58	87.32	9.86	p__Cyanobacteria	89	544	424	133	397	14	0	0	0	72.17	2.5
Xanthomonadaceae_MAG48f	Unknown	4060943	245	25552	62.82	85.92	0.00	f__Xanthomonadaceae	55	659	290	49	597	12	1	0	0	95.69	2.24
Cecembia_ calidifontis_MAG49s	Belliella	4067355	204	34607	41.04	85.92	1.41	o__Cytophagales	28	439	266	47	389	3	0	0	0	91.1	0.94
Sphingomonadaceae_MAG50f	Unknown	2515818	320	10297	67.47	85.92	2.82	c__Alphaproteobacteria	564	349	230	55	292	2	0	0	0	87.98	0.87
Capsulimonadaceae_MAG51f	Unknown	3703043	665	6064	63.99	85.92	4.23	k__Bacteria	924	161	108	31	126	4	0	0	0	76.57	1.73
Bradyrhizobium_MAG52g	Bradyrhizobium	5766360	626	13724	62.24	84.51	1.41	f__Bradyrhizobiaceae	47	693	296	59	605	27	2	0	0	91.63	6.09
UKL13-2_MAG53f	Unknown	2993940	633	5373	52.59	84.51	1.41	c__Betaproteobacteria	323	387	234	59	322	6	0	0	0	85.75	1.26
JACDGC01_MAG54f	Unknown	4076001	831	5585	57.29	84.51	2.82	k__Bacteria	924	151	101	33	115	3	0	0	0	79.73	2.97
Phormidesmis_A priestleyi_B_MAG55s	Leptolyngbya	3609501	480	8841	49.44	84.51	2.82	p__Cyanobacteria	89	544	424	92	445	6	1	0	0	80.95	1.53
UBA5177_MAG56c	Unknown	4563212	885	5536	67.99	84.51	4.23	k__Bacteria	924	160	109	30	122	8	0	0	0	75.69	4.33
Polyangiaceae_MAG57f	Labilithrix	5712704	946	6896	64.77	84.51	7.04	c__Deltaproteobacteria	83	247	155	66	177	4	0	0	0	74.15	1.64
Ferruginibacter_MAG58g	Unknown	4091667	812	6024	37.06	84.51	9.86	p__Bacteroidetes	364	303	203	36	255	12	0	0	0	90.01	3.87
Lapillicoccus_MAG59g	Unknown	3256706	382	10773	69.57	83.10	1.41	o__Actinomycetales	488	310	185	56	249	5	0	0	0	81.5	1.26
Nodosilinea_MAG60g	Unknown	2559317	740	3583	57.28	83.10	2.82	p__Cyanobacteria	129	472	368	163	304	5	0	0	0	62.18	1.09
Capsulimonadaceae_MAG61f	Unknown	2915558	669	4501	63.50	80.28	4.23	k__Bacteria	924	161	108	61	96	4	0	0	0	52.66	0.8
Ascidiaceihabitans_MAG62g	Sulfitobacter	2223744	556	4225	50.99	80.28	4.23	f__Rhodobacteraceae	56	582	313	149	421	12	0	0	0	72.15	1.73
Sphingomicrobium_MAG63g	Sphingomonas	1824331	443	4446	64.65	80.28	7.04	c__Alphaproteobacteria	564	345	226	102	236	7	0	0	0	68.67	1.78
Lysinimonas_A_MAG64g	Lysinimonas	2485453	163	20769	66.28	78.87	1.41	o__Actinomycetales	69	400	198	39	336	25	0	0	0	91.25	5.38
Fimbriimonas_MAG65g	Fimbriimonas	2967189	515	6437	51.23	77.46	0.00	k__Bacteria	924	161	108	30	129	2	0	0	0	78.57	1.85
Capsulimonadaceae_MAG66f	Unknown	4204385	780	5915	65.14	76.06	0.00	k__Bacteria	924	161	108	36	122	3	0	0	0	77.2	1.13
CAHJWT01_MAG67g	Sphingomonas	2323251	375	7206	66.66	76.06	1.41	c__Alphaproteobacteria	564	349	230	103	242	2	2	0	0	72.68	1.45
Fimbriimonadaceae_MAG68f	Fimbriimonas	2134956	206	13635	56.75	74.65	2.82	k__Bacteria	924	161	108	26	135	0	0	0	0	83.67	0
Bryobacteraceae_MAG69f	Candidatus Solibacter	3840205	557	8316	57.14	73.24	2.82	k__Bacteria	2258	184	114	48	131	5	0	0	0	66.54	3.03
JACDBZ01_MAG70g	Unknown	2957152	263	15831	67.08	73.24	4.23	k__Bacteria	924	160	109	27	128	5	0	0	0	92.15	2.39
Nostoc_MAG71g	Nostoc	5614383	1271	4863	41.94	70.42	9.86												
PJMF01_MAG72g	Unknown	1237930	82	22880	39.70	81.69	4.23	k__Bacteria	924	151	101	43	106	1	1	0	0	64.46	2.97
PPGL01_MAG73g	Unknown	1175402	195	7155	34.97	73.24	2.82	k__Bacteria	924	151	101	48	103	0	0	0	0	60.25	0
Saccharimonadaceae_MAG74f	Unknown_Candidatus_Sacchari	763139	102	9802	44.66	73.24	4.23	k__Bacteria	901	171	117	58	108	5	0	0	0	56.41	2.66

Supplementary Table 11 Table showing depth of coverage of each MAG after mapping reads.

	Cryoconite						Sea			Soil								
**MAGs**	**AB_18**	**ML_17**	**ML_18**	**VB_17**	**VB_18**	**VL_18**	**SS1**	**SS2**	**SS3**	**F3T3_Lud**	**F3T3_FD**	**F3T3_PM**	**F1T3**	**F1T4**	**F2T2**	**F3T1**	**F2T4**	**sum of coverage**
UBA5704_MAG01f	0.01	0	0	0	0.01	0	0	0	0	3.79	22.13	12.44	1.15	0.09	0.03	0.03	0.34	**40.04**
Bog-756_MAG02g	5.1	10.67	11.34	10.16	14.38	0	0	0	0	0.02	0.1	0.03	0.02	0.03	0.01	0.01	0.01	**51.9**
Tepidicella_MAG03g	0.34	0.02	0.02	0.03	0.04	0.13	0	0	0	68.7	0.18	0.09	0.07	0.06	0.06	0.17	0.11	**70.02**
Ferrovibrionales_MAG04o	0.01	0	0	0	0	0	0	0	0	33.55	0.05	0.02	0.02	0.01	0.01	0.02	0.02	**33.72**
Palsa-881_MAG05g	0.15	5.42	5.67	4.73	5.71	0.01	0	0	0	0.04	0.06	0.04	0.02	0.01	0.02	0.02	0.02	**21.92**
Cytophagaceae_MAG06f	0.02	0.01	0.01	0.01	0.01	0.01	0.02	0.03	0.03	175.73	0.03	0.01	0.01	0.01	0.01	0.01	0.01	**175.96**
Iso899_MAG07g	0.04	0.01	0.01	0.01	0.01	0.02	0	0	0	0.07	46.84	5.75	0.36	0.46	0.08	0.13	0.11	**53.89**
Nanopelagicales_MAG08o	7.21	2.15	3.06	2.79	2.75	0.04	0	0	0	0	0.03	0.01	0.01	0.01	0.01	0.01	0.01	**18.1**
UBA2796_MAG09f	13.48	8.41	6.84	9.97	10.75	0	0	0	0	0	0	0	0	0	0	0	0	**49.46**
Sphingomonadaceae_MAG10f	2.26	6.73	4.08	5	7.61	0	0	0	0	0.03	0.04	0.04	0.01	0.01	0.02	0.02	0.01	**25.86**
Allosphingosinicella_MAG11g	0.16	0.01	0.01	0.01	0.01	0.03	0.01	0	0	30.24	26.9	25.92	0.75	0.32	0.15	0.19	0.41	**85.13**
Rhodoglobus_MAG12g	13.8	1.73	1.93	2.28	1.66	0.02	0	0.01	0	0.02	0.04	0.01	0.01	0.01	0.01	0.09	0.02	**21.64**
SCTD01_MAG13f	79.08	3.68	4.61	9.45	5.89	0.84	0	0	0	0.09	0.98	0.5	0.18	0.32	0.29	0.35	0.1	**106.36**
JAAFJT01_MAG14g	0.35	4.12	14.7	3	12.98	0	0	0	0	0	0	0	0	0	0	0	0	**35.16**
UBA1936_MAG15g	9.68	3.52	3.94	5.07	2.98	0.02	0	0	0	0.03	0.03	0.04	0.01	0.01	0.02	0.05	0.01	**25.43**
Rhizobacter_MAG16g	20.07	3.49	3.99	6.08	11.86	0.41	0	0	0	0.73	2.15	1.69	0.77	0.32	0.36	0.53	0.75	**53.19**
AG11_sp014378185_MAG17s	25.54	1.93	3.63	4.54	5.66	0.06	0	0	0	0.03	0.21	0.16	0.08	0.07	0.02	0.05	0.04	**42.03**
UBA11236_MAG18f	43.23	0	0	0.02	0.83	0.13	0	0	0	0	0	0	0	0	0	0	0	**44.24**
Granulicellac_MAG19g	11.37	10.15	7.16	13.28	6.07	0	0	0	0	0	0	0	0	0	0	0	0	**48.04**
Cyclobacteriaceae_MAG20f	0	0	0	0	0	0	0	0	0	29.92	0	0	0	0	0	0	0	**29.93**
Lacisediminihabitans_MAG21g	32.71	9.94	13.97	17.14	10.33	0.23	0	0	0	0.02	0.04	0.01	0.02	0.01	0.02	0.12	0.02	**84.59**
Pararhodobacter_MAG22g	0.02	0	0	0	0	0.01	0	0	0	36.94	0.09	0.04	0.03	0.02	0.05	0.03	0.02	**37.27**
ASP10-02a_sp002335115_MAG23s	0	0	0	0	0	0	14.29	29.58	21.08	0	0	0	0	0	0	0	0	**64.95**
Ferruginibacter_sp014377975_MAG24s	4.39	1.77	2.13	5.79	5.13	0.02	0	0	0	0	0.01	0.01	0	0.01	0.01	0.01	0.01	**19.29**
SCTD01_MAG25f	14.43	6.36	7.88	7.25	6.1	0.1	0	0	0	0.07	0.55	0.25	0.13	0.19	0.17	0.25	0.08	**43.82**
UBA3362_MAG26g	0	0	0	0	0	0	0	0	0	0.1	0.53	0.08	0.32	0.08	0.04	16.99	0.01	**18.15**
UBA11741_MAG27g	0	0	0	0	0	0	0	0	0	10.88	4.6	4.76	1.14	1.16	0	0	0.04	**22.58**
CAHJWT01_MAG28g	1.86	2.53	1.44	7.34	2.91	0.01	0	0	0	0.12	0.15	0.15	0.05	0.02	0.07	0.08	0.09	**16.81**
UBA11400_MAG29g	0	0	0	0	0	0	0	0	0	29.17	0	0	0	0	0	0	0	**29.17**
Chthonomonadaceae_MAG30f	9.44	1.91	1.29	2.3	1.79	0	0	0	0	0	0	0	0	0	0	0	0	**16.75**
UBA11236_MAG31f	22.3	0	0	0	0.1	0.24	0	0	0	0	0	0	0	0	0	0.01	0	**22.66**
Microcoleus_MAG32g	31.6	0	0.01	1.21	0.14	7.49	0	0.01	0	0	0.29	0.14	0.01	0.01	0.03	0.01	0	**40.95**
CAHJXG01_MAG33g	5.04	3.16	3.66	4.35	2.85	0.01	0	0	0	0.01	0.05	0.02	0.03	0.02	0.01	0.01	0.01	**19.22**
UKL13-2_MAG34f	33.14	0	0.01	0.28	1.87	0.21	0	0	0	0	0	0	0	0	0	0.01	0	**35.53**
JACMQW01_MAG35g	0.07	3.58	3.02	4.76	1.54	0	0	0	0	0.02	0.03	0.01	0.01	0.01	0.01	0.01	0.01	**13.07**
Pseudanabaena_MAG36g	11.12	3.51	9.76	4.47	3.29	0.47	0	0	0	0.01	0.03	0.02	0	0	0.06	0.09	0.01	**32.85**
CAHJXF01_MAG37g	14.85	0.1	0.06	0.65	1.04	0.27	0	0	0	0.1	0.25	0.16	0.13	0.04	0.05	0.09	0.13	**17.92**
UBA10364_MAG38g	0	0	0	0	0	0	3.65	12.12	9.04	0	0	0	0	0	0	0	0	**24.82**
WLMZ01_MAG39g	8.92	1.62	1.72	2.11	1.8	0.03	0	0	0	0.03	0.2	0.04	0.05	0.05	0.02	0.02	0.04	**16.67**
UBA6161_MAG40g	0.01	0	0	0	0	0	0	0	0	18.91	0.01	0	0	0.01	0.01	0.01	0	**18.96**
Rhizobacter_MAG41g	11.51	10	9	5.91	6.5	1.01	0	0	0	0.46	1.23	0.99	0.39	0.22	0.25	0.39	0.37	**48.25**
DTNP01_MAG42g	21.7	31.64	20.51	21.98	24.1	0.1	0	0	0	0.02	0.05	0.02	0.01	0.02	0.01	0.01	0.01	**120.19**
AC-14_MAG43f	0	0	0	0	0	0	0	0	0	7.33	7.08	8.44	0.59	0.92	0.2	0.03	0.06	**24.65**
Palsa-1515_MAG44g	24.49	43.34	32.39	43.87	35.25	0	0	0	0	0	0	0	0	0	0	0.01	0	**179.35**
JACDGC01_MAG45f	0.94	4.19	3.03	1.55	1.1	0	0	0	0	0	0	0	0	0	0	0	0	**10.82**
JABFXX01_MAG46g	1.19	2.28	4	1.67	2.13	0	0	0	0	0	0.05	0.03	0.02	0.01	0.01	0.03	0.02	**11.45**
ULC077BIN1_MAG47g	0.05	0.01	0.01	0.04	0.01	24.95	0	0	0	0.01	0.34	0.13	0.09	0.07	0.48	0.11	0.1	**26.39**
Xanthomonadaceae_MAG48f	0.23	2.25	0.81	7.51	6.88	0.02	0	0.01	0	0.02	0.05	0.04	0.02	0.02	0.02	0.04	0.02	**17.94**
Cecembia_calidifontis_MAG49s	0	0	0	0	0	0	0	0	0	35.3	0	0	0	0	0	0	0	**35.3**
Sphingomonadaceae_MAG50f	4.87	5.32	2.61	11.88	6.19	0.01	0	0	0	0.05	0.08	0.07	0.03	0.02	0.03	0.03	0.03	**31.23**
Capsulimonadaceae_MAG51f	5.71	5.34	3.36	7.92	5.41	0	0	0	0	0	0.01	0	0	0	0	0	0	**27.76**
Bradyrhizobium_MAG52g	15.28	1.09	1.15	0.95	1.15	0.13	0	0	0	0.31	1.19	0.35	0.52	0.43	0.29	0.51	0.56	**23.9**
UKL13-2_MAG53f	18.55	0.02	0.32	0.44	0.74	0.67	0	0	0	0	0	0	0	0	0	0	0	**20.75**
JACDGC01_MAG54f	1.64	2.3	1.5	3.37	3.03	0	0	0	0	0	0	0	0	0	0	0	0	**11.85**
Phormidesmis_A_priestleyi_B_MAG55s	62.23	151	146.87	184.15	103.68	6.87	0	0	0	0	0.27	0.08	0.07	0.13	0.07	0.06	0.01	**655.5**
UBA5177_MAG56c	3.7	7.52	6.67	6.66	6.67	0	0	0	0	0	0.02	0	0	0	0	0	0	**31.26**
Polyangiaceae_MAG57f	0.07	0.22	0.33	0.52	10.04	0	0	0	0	0	0.02	0.01	0	0	0	0.01	0	**11.23**
Ferruginibacter_MAG58g	29.56	0.02	0.02	0.14	0.09	0.42	0	0	0	0	0.04	0.01	0.01	0.03	0.03	0.07	0.02	**30.47**
Lapillicoccus_MAG59g	5.51	2.77	3.77	3.8	4.46	0.23	0	0	0	0.04	0.53	0.16	0.19	0.16	0.18	0.22	0.28	**22.3**
Nodosilinea_MAG60g	0	0	0	0	0	0	0	0	0	0.09	0.35	0.22	0.16	0.09	6.58	8.86	0.09	**16.44**
Capsulimonadaceae_MAG61f	5.34	14.61	10.98	12.55	10.87	0	0	0	0	0	0	0	0	0	0	0	0	**54.36**
Ascidiaceihabitans_MAG62g	0	0	0	0	0	0	3.1	9.63	6.31	0	0	0	0	0	0	0	0	**19.05**
Sphingomicrobium_MAG63g	31.21	0	0.01	0.26	0.11	1.96	0	0	0	0.68	2.86	3.98	0.74	0.62	1.06	0.92	0.47	**44.87**
Lysinimonas_A_MAG64g	28.68	16.12	17.68	15.17	8.18	0.03	0	0	0	0.03	0.07	0.02	0.02	0.02	0.01	0.09	0.04	**86.18**
Fimbriimonas_MAG65g	10.52	0	0	0.25	0.76	0	0	0	0	0	0	0	0	0	0	0	0	**11.54**
Capsulimonadaceae_MAG66f	1.14	8.22	5.59	6.57	4.31	0	0	0	0	0	0	0	0	0	0	0	0	**25.84**
CAHJWT01_MAG67g	2.02	3.29	5.02	5.5	3.44	0.01	0	0	0	0.14	0.16	0.14	0.05	0.02	0.07	0.09	0.08	**20.01**
Fimbriimonadaceae_MAG68f	0.01	0	0	0	0	0	0	0	0	19.93	0.01	0	0	0	0	0	0	**19.96**
Bryobacteraceae_MAG69f	13.11	7.83	5.56	10.79	8.03	0	0	0	0	0	0.01	0	0	0	0	0	0	**45.35**
JACDBZ01_MAG70g	0	0	0	0	0	0	0	0	0	0.8	16.27	3.35	0.44	13.92	0.02	0.03	0.14	**34.98**
Nostoc_MAG71g	4.95	0.01	0.03	0.03	0.02	22.22	0	0	0	0	1.75	0.77	2.96	0.28	9.96	1.13	0.97	**45.09**
PJMF01_MAG72g	0.99	4.21	3.13	4.72	11.29	0	0	0	0	0	0	0	0	0	0	0	0	**24.35**
PPGL01_MAG73g	0.13	4.34	2.37	3.57	1.54	0	0	0.01	0	0	0	0	0	0	0	0	0	**11.98**
Saccharimonadaceae_MAG74f	0	0	0	0	0	0	0	0	0	5.08	14.81	4.25	0.03	0.25	0.01	0	0	**24.42**

Supplementary Table 12 GTDB-Tk classification of MAGs

MAG	Phylum	Class	Order	Family	Genus	Closest Placement ANI	Closest Placement Alignment Fraction	Classification Method	MSA AA Percent	RED Value
Granulicellac_MAG19g	Acidobacteriota	Acidobacteriae	Acidobacteriales	Acidobacteriaceae	*Granulicella_C*	-	-	C: TOP+ANI:	85.57	0.97
Bryobacteraceae_MAG69f	Acidobacteriota	Acidobacteriae	Bryobacterales	Bryobacteraceae		-	-	N: RED:	68.39	0.87
UBA11741_MAG27g	Acidobacteriota	Blastocatellia	Pyrinomonadales	Pyrinomonadaceae	UBA11741	81.44	0.65	C: TOP+ANI:	86.34	0.99
UBA5704_MAG01f	Acidobacteriota	Thermoanaerobaculia	UBA5704	UBA5704		-	-	C: TOP:	92.4	0.78
AC-14_MAG43f	Actinobacteriota	Acidimicrobiia	Acidimicrobiales	AC-14		77.43	0.28	C: TOP+ANI:	75.44	0.84
WLMZ01_MAG39g	Actinobacteriota	Acidimicrobiia	Acidimicrobiales	Ilumatobacteraceae	WLMZ01	77.98	0.3	C: TOP+ANI:	85.29	0.92
Bog-756_MAG02g	Actinobacteriota	Acidimicrobiia	Acidimicrobiales	RAAP-2	Bog-756	81.15	0.51	C: TOP+ANI:	89.1	0.96
Lapillicoccus_MAG59g	Actinobacteriota	Actinomycetia	Actinomycetales	Dermatophilaceae	*Lapillicoccus*	80.52	0.57	C: TOP+ANI:	77.45	0.91
Lacisediminihabitans_MAG21g	Actinobacteriota	Actinomycetia	Actinomycetales	Microbacteriaceae	*Lacisediminihabitans*	83.79	0.72	C: TOP+ANI:	83.92	0.99
Lysinimonas_A_MAG64g	Actinobacteriota	Actinomycetia	Actinomycetales	Microbacteriaceae	*Lysinimonas_A*	80.74	0.57	C: TOP+ANI:	82.15	0.98
Rhodoglobus_MAG12g	Actinobacteriota	Actinomycetia	Actinomycetales	Microbacteriaceae	*Rhodoglobus*	77.79	0.26	C: TOP+ANI:	92.26	0.97
Iso899_MAG07g	Actinobacteriota	Actinomycetia	Mycobacteriales	Jatrophihabitantaceae	Iso899	-	-	N: RED:	88.35	0.92
SCTD01_MAG13f	Actinobacteriota	Actinomycetia	Mycobacteriales	SCTD01		-	-	N: RED:	91.66	0.87
SCTD01_MAG25f	Actinobacteriota	Actinomycetia	Mycobacteriales	SCTD01		-	-	N: RED:	87.51	0.86
Nanopelagicales_MAG08o	Actinobacteriota	Actinomycetia	Nanopelagicales			-	-	N: RED:	95.43	0.68
Capsulimonadaceae_MAG51f	Armatimonadota	Armatimonadia	Armatimonadales	Capsulimonadaceae		-	-	N: RED:	67.6	0.83
Capsulimonadaceae_MAG61f	Armatimonadota	Armatimonadia	Armatimonadales	Capsulimonadaceae		-	-	N: RED:	46.75	0.84
Capsulimonadaceae_MAG66f	Armatimonadota	Armatimonadia	Armatimonadales	Capsulimonadaceae		-	-	N: RED:	66.05	0.83
Chthonomonadaceae_MAG30f	Armatimonadota	Chthonomonadetes	Chthonomonadales	Chthonomonadaceae		-	-	N: RED:	86.24	0.80
Fimbriimonas_MAG65g	Armatimonadota	Fimbriimonadia	Fimbriimonadales	Fimbriimonadaceae	*Fimbriimonas*	-	-	C: TOP:	73.2	0.93
Fimbriimonadaceae_MAG68f	Armatimonadota	Fimbriimonadia	Fimbriimonadales	Fimbriimonadaceae		-	-	N: RED:	77.76	0.86
Ferruginibacter_sp014377975_MAG24s	Bacteroidota	Bacteroidia	Chitinophagales	Chitinophagaceae	*Ferruginibacter*	99.04	0.79	C: TOP+ANI: *	91.46	-
Ferruginibacter_MAG58g	Bacteroidota	Bacteroidia	Chitinophagales	Chitinophagaceae	*Ferruginibacter*	79.03	0.45	C: TOP+ANI:	75.86	0.97
JAAFJT01_MAG14g	Bacteroidota	Bacteroidia	Chitinophagales	Saprospiraceae	JAAFJT01	-	-	N: RED:	84.4	0.86
UBA3362_MAG26g	Bacteroidota	Bacteroidia	Chitinophagales	Saprospiraceae	UBA3362	-	-	C: TOP:	87.73	0.91
Cecembia_ calidifontis_MAG49s	Bacteroidota	Bacteroidia	Cytophagales	Cyclobacteriaceae	*Cecembia*	98.33	0.92	C: TOP+ANI: *	82.29	-
Cyclobacteriaceae_MAG20f	Bacteroidota	Bacteroidia	Cytophagales	Cyclobacteriaceae		-	-	N: RED:	95.43	0.80
Cytophagaceae_MAG06f	Bacteroidota	Bacteroidia	Cytophagales	Cytophagaceae		-	-	N: RED:	97.22	0.85
UBA10364_MAG38g	Bacteroidota	Bacteroidia	Flavobacteriales	Schleiferiaceae	UBA10364	87.16	0.83	C: TOP+ANI:	91.64	0.99
UBA6161_MAG40g	Bacteroidota	Bacteroidia	NS11-12g	UBA955	UBA6161	-	-	N: RED:	83.26	0.86
UBA11400_MAG29g	Bacteroidota	Rhodothermia	Balneolales	HLUCCA01	UBA11400	-	-	N: RED:	90.19	0.89
JACMQW01_MAG35g	Bdellovibrionota	Bdellovibrionia_A	UBA1018	UBA1018	JACMQW01	-	-	N: RED:	87.19	0.94
UBA2796_MAG09f	Chloroflexota	Anaerolineae	SBR1031	UBA2796		-	-	N: RED:	84.53	0.82
JACDBZ01_MAG70g	Chloroflexota	Ellin6529	QHBO01	QHBO01	JACDBZ01	81.07	0.67	C: TOP+ANI:	79.87	0.95
JACDGC01_MAG45f	Chloroflexota	Ktedonobacteria	Ktedonobacterales	JACDGC01		-	-	N: RED:	77.07	0.83
JACDGC01_MAG54f	Chloroflexota	Ktedonobacteria	Ktedonobacterales	JACDGC01		-	-	N: RED:	69.23	0.83
DTNP01_MAG42g	Chloroflexota	Ktedonobacteria				-	-	N: RED:	81.34	0.88
UBA5177_MAG56c	Chloroflexota	UBA5177				-	-	N: RED:	71.13	0.52

MAG	Phylum	Class	Order	Family	Genus	Closest Placement ANI	Closest Placement Alignment Fraction	Classification Method	MSA AA Percent	RED Value
Microcoleus_MAG32g	Cyanobacteria	Cyanobacteriia	Cyanobacteriales	Microcoleaceae	*Microcoleus*	89.62	0.81	C: TOP+ANI:	81.97	0.99
Nostoc_MAG71g	Cyanobacteria	Cyanobacteriia	Cyanobacteriales	Nostocaceae	*Nostoc*	-	-	C: TOP+ANI:	60.57	0.96
Phormidesmis_A priestleyi_B_MAG55s	Cyanobacteria	Cyanobacteriia	Leptolyngbyales	Leptolyngbyaceae	*Phormidesmis_A*	99.27	0.97	C: TOP+ANI: *	78.68	-
ULC077BIN1_MAG47g	Cyanobacteria	Cyanobacteriia	Leptolyngbyales	Leptolyngbyaceae	ULC077BIN1	83.82	0.8	C: TOP+ANI:	67.68	0.98
Nodosilinea_MAG60g	Cyanobacteria	Cyanobacteriia	Phormidesmiales	Phormidesmiaceae	*Nodosilinea*	-	-	C: TOP+ANI:	62.6	0.98
Pseudanabaena_MAG36g	Cyanobacteria	Cyanobacteriia	Pseudanabaenales	Pseudanabaenaceae	*Pseudanabaena*	-	-	C: TOP+ANI:	89.12	0.97
Palsa-1515_MAG44g	Eremiobacterota	Eremiobacteria	Baltobacterales	Baltobacteraceae	Palsa-1515	77.44	0.36	C: TOP+ANI:	79.59	0.94
UBA11236_MAG18f	Fibrobacterota	Fibrobacteria	UBA11236	UBA11236		-	-	N: RED:	85.25	0.76
UBA11236_MAG31f	Fibrobacterota	Fibrobacteria	UBA11236	UBA11236		-	-	N: RED:	87.77	0.77
AG11_sp014378185_MAG17s	Gemmatimonadota	Gemmatimonadetes	Gemmatimonadales	Gemmatimonadaceae	AG11	96.39	0.72	C: TOP+ANI: *	81.6	-
JABFXX01_MAG46g	Myxococcota	Polyangia	Haliangiales	Haliangiaceae	JABFXX01	79.12	0.5	C: TOP+ANI:	74.79	0.94
Polyangiaceae_MAG57f	Myxococcota	Polyangia	Polyangiales	Polyangiaceae		-	-	C: TOP:	67.2	0.80
PPGL01_MAG73g	Patescibacteria	Microgenomatia	Levybacterales	UBA12049	PPGL01	-	-	N: RED:	54.28	0.88
PJMF01_MAG72g	Patescibacteria	Microgenomatia	UBA1400	PJMF01	PJMF01	-	-	N: RED:	62.62	0.88
Saccharimonadaceae_MAG74f	Patescibacteria	Saccharimonadia	Saccharimonadales	Saccharimonadaceae		-	-	C: TOP:	57.06	0.75
CAHJXG01_MAG33g	Proteobacteria	Alphaproteobacteria	Acetobacterales	Acetobacteraceae	CAHJXG01	77.98	0.25	C: TOP+ANI:	82.19	0.91
Palsa-881_MAG05g	Proteobacteria	Alphaproteobacteria	Caulobacterales	Caulobacteraceae	Palsa-881	77.07	0.31	C: TOP+ANI:	92.91	0.96
Ferrovibrionales_MAG04o	Proteobacteria	Alphaproteobacteria	Ferrovibrionales			-	-	N: RED:	93.01	0.70
Bradyrhizobium_MAG52g	Proteobacteria	Alphaproteobacteria	Rhizobiales	Xanthobacteraceae	*Bradyrhizobium*	84.14	0.61	C: TOP+ANI:	77.09	0.98
Ascidiaceihabitans_MAG62g	Proteobacteria	Alphaproteobacteria	Rhodobacterales	Rhodobacteraceae	*Ascidiaceihabitans*	89.75	0.78	C: TOP+ANI:	68.45	1.00
Pararhodobacter_MAG22g	Proteobacteria	Alphaproteobacteria	Rhodobacterales	Rhodobacteraceae	*Pararhodobacter*	-	-	ANI	86.8	0.92
Allosphingosinicella_MAG11g	Proteobacteria	Alphaproteobacteria	Sphingomonadales	Sphingomonadaceae	*Allosphingosinicella*	80.7	0.49	C: TOP+ANI:	91.34	0.93
CAHJWT01_MAG28g	Proteobacteria	Alphaproteobacteria	Sphingomonadales	Sphingomonadaceae	CAHJWT01	-	-	N: RED:	82.29	0.91
CAHJWT01_MAG67g	Proteobacteria	Alphaproteobacteria	Sphingomonadales	Sphingomonadaceae	CAHJWT01	81.34	0.47	C: TOP+ANI:	56.52	0.97
Sphingomicrobium_MAG63g	Proteobacteria	Alphaproteobacteria	Sphingomonadales	Sphingomonadaceae	*Sphingomicrobium*	-	-	ANI	54.93	0.95
UBA1936_MAG15g	Proteobacteria	Alphaproteobacteria	Sphingomonadales	Sphingomonadaceae	UBA1936	77.33	0.24	C: TOP+ANI:	95.99	0.95
Sphingomonadaceae_MAG10f	Proteobacteria	Alphaproteobacteria	Sphingomonadales	Sphingomonadaceae		-	-	C: TOP:	93.17	0.77
Sphingomonadaceae_MAG50f	Proteobacteria	Alphaproteobacteria	Sphingomonadales	Sphingomonadaceae		-	-	C: TOP:	79.13	0.82
CAHJXF01_MAG37g	Proteobacteria	Gammaproteobacteria	Burkholderiales	Burkholderiaceae	CAHJXF01	83.76	0.59	C: TOP+ANI:	82.81	0.95
Rhizobacter_MAG16g	Proteobacteria	Gammaproteobacteria	Burkholderiales	Burkholderiaceae	*Rhizobacter*	82.35	0.61	C: TOP+ANI:	87.39	0.98
Rhizobacter_MAG41g	Proteobacteria	Gammaproteobacteria	Burkholderiales	Burkholderiaceae	*Rhizobacter*	80.97	0.61	C: TOP+ANI:	82.91	0.98
Tepidicella_MAG03g	Proteobacteria	Gammaproteobacteria	Burkholderiales	Burkholderiaceae	*Tepidicella*	-	-	N: RED:	96.07	0.97
UKL13-2_MAG34f	Proteobacteria	Gammaproteobacteria	Burkholderiales	UKL13-2		-	-	N: RED:	83.18	0.89
UKL13-2_MAG53f	Proteobacteria	Gammaproteobacteria	Burkholderiales	UKL13-2		-	-	N: RED:	77.25	0.89
ASP10-02a_sp002335115_MAG23s	Proteobacteria	Gammaproteobacteria	Pseudomonadales	Nitrincolaceae	ASP10-02a	95.44	0.86	C: TOP+ANI: *	90.75	-
Xanthomonadaceae_MAG48f	Proteobacteria	Gammaproteobacteria	Xanthomonadales	Xanthomonadaceae		-	-	C: TOP:	86.44	0.86

N: RED — taxonomic novelty determined using RED
C: TOP — taxonomic classification fully defined by topology
C: TOP+ANI: — taxonomic classification defined by topology and ANI
* — topological placement and ANI have congruent species assignments

Supplementary Table 13: Species and closest related relative.

MAG	Genus	Species	FastANI Reference	FastANI Ref Radius	FastANI ANI	FastANI Alignment Fraction	Closest Placement Reference	Closest Placement ANI	Closest Placement Alignment Fraction	Classification Method	Other Related References
Granulicellac_MAG19g	*Granulicella_C*		-	-	-	-	GCA_903970285.1	-	-	C: TOP+ANI:	GCF_000178975.2
Bryobacteraceae_MAG69f			-	-	-	-	-	-	-	N: RED:	-
UBA11741_MAG27g	UBA11741		-	-	-	-	GCA_014380365.1	81.44	0.65	C: TOP+ANI:	GCA_002427845.1
UBA5704_MAG01f			-	-	-	-	-	-	-	C: TOP:	-
AC-14_MAG43f			-	-	-	-	GCA_902805665.1	77.43	0.28	C: TOP+ANI:	-
WLMZ01_MAG39g	WLMZ01		-	-	-	-	GCA_009726095.1	77.98	0.3	C: TOP+ANI:	-
Bog-756_MAG02g	Bog-756		-	-	-	-	GCA_003153575.1	81.15	0.51	C: TOP+ANI:	GCA_003164095.1
Lapillicoccus_MAG59g	*Lapillicoccus*		-	-	-	-	GCF_006715055.1	80.52	0.57	C: TOP+ANI:	-
Lacisediminihabitans_MAG21g	*Lacisediminihabitans*		-	-	-	-	GCF_014217685.1	83.79	0.72	C: TOP+ANI:	GCF_008040105.1
Lysinimonas_A_MAG64g	*Lysinimonas_A*		-	-	-	-	GCA_011620705.1	80.74	0.57	C: TOP+ANI:	GCA_002325245.1
Rhodoglobus_MAG12g	*Rhodoglobus*		-	-	-	-	GCA_014645015.1	77.79	0.26	C: TOP+ANI:	GCA_903920435.1
Iso899_MAG07g	Iso899		-	-	-	-	-	-	-	N: RED:	-
SCTD01_MAG13f			-	-	-	-	-	-	-	N: RED:	-
SCTD01_MAG25f			-	-	-	-	-	-	-	N: RED:	-
Nanopelagicales_MAG08o			-	-	-	-	-	-	-	N: RED:	-
Capsulimonadaceae_MAG51f			-	-	-	-	-	-	-	N: RED:	-
Capsulimonadaceae_MAG61f			-	-	-	-	-	-	-	N: RED:	-
Capsulimonadaceae_MAG66f			-	-	-	-	-	-	-	N: RED:	-
Chthonomonadaceae_MAG30f			-	-	-	-	-	-	-	N: RED:	-
Fimbriimonas_MAG65g	*Fimbriimonas*		-	-	-	-	-	-	-	C: TOP:	-
Fimbriimonadaceae_MAG68f			-	-	-	-	-	-	-	N: RED:	-
Ferruginibacter_sp014377975_MAG24s	*Ferruginibacter*	*Ferruginibacter* sp014377975	GCA_014377975.1	95	99.04	0.79	GCA_014377975.1	99.04	0.79	C: TOP+ANI: *	-
Ferruginibacter_MAG58g	*Ferruginibacter*		-	-	-	-	GCA_014377975.1	79.03	0.45	C: TOP+ANI:	GCA_013141115.1
JAAFJT01_MAG14g	JAAFJT01		-	-	-	-	-	-	-	N: RED:	-
UBA3362_MAG26g	UBA3362		-	-	-	-	-	-	-	C: TOP:	-
Cecembia_ calidifontis_MAG49s	*Cecembia*	*Cecembia calidifontis*	GCF_004216715.1	95	98.33	0.92	GCF_004216715.1	98.33	0.92	C: TOP+ANI: *	GCF_003014575.1
Cyclobacteriaceae_MAG20f			-	-	-	-	-	-	-	N: RED:	-
Cytophagaceae_MAG06f			-	-	-	-	-	-	-	N: RED:	-
UBA10364_MAG38g	UBA10364		-	-	-	-	GCA_003487785.1	87.16	0.83	C: TOP+ANI:	-
UBA6161_MAG40g	UBA6161		-	-	-	-	-	-	-	N: RED:	-
UBA11400_MAG29g	UBA11400		-	-	-	-	-	-	-	N: RED:	-
JACMQW01_MAG35g	JACMQW01		-	-	-	-	-	-	-	N: RED:	-
UBA2796_MAG09f			-	-	-	-	-	-	-	N: RED:	-
JACDBZ01_MAG70g	JACDBZ01		-	-	-	-	GCA_013694645.1	81.07	0.67	C: TOP+ANI:	GCA_013820865.1
JACDGC01_MAG45f			-	-	-	-	-	-	-	N: RED:	-
JACDGC01_MAG54f			-	-	-	-	-	-	-	N: RED:	-
DTNP01_MAG42g			-	-	-	-	GCA_903970285.2	-	-	N: RED:	GCF_000178975.2
UBA5177_MAG56c			-	-	-	-	-	-	-	N: RED:	-

MAG	Genus	Species	FastANI Reference	FastANI Ref Radius	FastANI ANI	FastANI Alignment Fraction	Closest Placement Reference	Closest Placement ANI	Closest Placement Alignment Fraction	Classification Method	Other Related References
Microcoleus_MAG32g	*Microcoleus*		-	-	-	-	GCF_003003725.1	89.62	0.81	C: TOP+ANI:	GCF_013179805.1
Nostoc_MAG71g	*Nostoc*		-	-	-	-	-	-	-	C: TOP+ANI:	GCF_003443655.1
Phormidesmis_A priestleyi_B_MAG55s	*Phormidesmis_A*	*Phormidesmis_A priestleyi_B*	GCF_001650195.1	95	99.27	0.97	GCF_001650195.1	99.27	0.97	C: TOP+ANI: *	GCF_001895925.1
ULC077BIN1_MAG47g	ULC077BIN1		-	-	-	-	GCA_003249025.1	83.82	0.8	C: TOP+ANI:	-
Nodosilinea_MAG60g	*Nodosilinea*		-	-	-	-	-	-	-	C: TOP+ANI:	GCA_003249105.1
Pseudanabaena_MAG36g	*Pseudanabaena*		-	-	-	-	-	-	-	C: TOP+ANI:	GCA_003242085.1
Palsa-1515_MAG44g	Palsa-1515		-	-	-	-	GCA_903970385.1	77.44	0.36	C: TOP+ANI:	-
UBA11236_MAG18f			-	-	-	-	-	-	-	N: RED:	-
UBA11236_MAG31f			-	-	-	-	-	-	-	N: RED:	-
AG11_sp014378185_MAG17s	AG11	AG11 sp014378185	GCA_014378185.1	95	96.39	0.72	GCA_014378185.1	96.39	0.72	C: TOP+ANI: *	GCA_013361335.1
JABFXX01_MAG46g	JABFXX01		-	-	-	-	GCA_013368795.1	79.12	0.5	C: TOP+ANI:	GCA_013812655.1
Polyangiaceae_MAG57f			-	-	-	-	-	-	-	C: TOP:	-
PPGL01_MAG73g	PPGL01		-	-	-	-	-	-	-	N: RED:	-
PJMF01_MAG72g	PJMF01		-	-	-	-	-	-	-	N: RED:	-
Saccharimonadaceae_MAG74f			-	-	-	-	-	-	-	C: TOP:	-
CAHJXG01_MAG33g	CAHJXG01		-	-	-	-	GCF_903644035.1	77.98	0.25	C: TOP+ANI:	-
Palsa-881_MAG05g	Palsa-881		-	-	-	-	GCA_003161535.1	77.07	0.31	C: TOP+ANI:	GCA_013815405.1
Ferrovibrionales_MAG04o			-	-	-	-	-	-	-	N: RED:	-
Bradyrhizobium_MAG52g	*Bradyrhizobium*		-	-	-	-	GCA_001464035.1	84.14	0.61	C: TOP+ANI:	GCF_900129425.1
Ascidiaceihabitans_MAG62g	*Ascidiaceihabitans*		-	-	-	-	GCA_002478745.1	89.75	0.78	C: TOP+ANI:	GCF_900302465.1
Pararhodobacter_MAG22g	*Pararhodobacter*		-	-	-	-	-	-	-	ANI	GCA_007131945.1
Allosphingosinicella_MAG11g	*Allosphingosinicella*		-	-	-	-	GCA_005882415.1	80.7	0.49	C: TOP+ANI:	GCF_004564275.1
CAHJWT01_MAG28g	CAHJWT01		-	-	-	-	-	-	-	N: RED:	-
CAHJWT01_MAG67g	CAHJWT01		-	-	-	-	GCF_903642255.1	81.34	0.47	C: TOP+ANI:	GCF_903643075.1
Sphingomicrobium_MAG63g	*Sphingomicrobium*		-	-	-	-	-	-	-	ANI	GCF_003499275.1
UBA1936_MAG15g	UBA1936		-	-	-	-	GCA_002336985.1	77.33	0.24	C: TOP+ANI:	GCA_013821585.1
Sphingomonadaceae_MAG10f			-	-	-	-	-	-	-	C: TOP:	-
Sphingomonadaceae_MAG50f			-	-	-	-	-	-	-	C: TOP:	-
CAHJXF01_MAG37g	CAHJXF01		-	-	-	-	GCF_903644065.1	83.76	0.59	C: TOP+ANI:	-
Rhizobacter_MAG16g	*Rhizobacter*		-	-	-	-	GCA_003152055.1	82.35	0.61	C: TOP+ANI:	GCF_013366375.1
Rhizobacter_MAG41g	*Rhizobacter*		-	-	-	-	GCA_003152055.1	80.97	0.61	C: TOP+ANI:	GCF_001425865.1
Tepidicella_MAG03g	*Tepidicella*		-	-	-	-	-	-	-	N: RED:	-
UKL13-2_MAG34f			-	-	-	-	-	-	-	N: RED:	-
UKL13-2_MAG53f			-	-	-	-	-	-	-	N: RED:	-
ASP10-02a_sp002335115_MAG23s	ASP10-02a	ASP10-02a sp002335115	GCA_002335115.1	95	95.44	0.86	GCA_002335115.1	95.44	0.86	C: TOP+ANI: *	GCA_002312935.1
Xanthomonadaceae_MAG48f			-	-	-	-	-	-	-	C: TOP:	-

N: RED	taxonomic novelty determined using RED
C: TOP	taxonomic classification fully defined by topology
C: TOP+ANI:	taxonomic classification defined by topology and ANI
*	topological placement and ANI have congruent species assignments

Supplementary Table 14 Geochemistry results

	Cryoconite									Forefield soil									
	VL-2	VL-1	AB-2	AB-1	VB-2	VB-1	ML-2	ML-1	Median	F1T0	F1T1	F1T2	F2T0	F2T1	F2T2	F2T4	F3T0	F3T1	Median
Total C (%)	2.97	3.64	2.08	1.82	3.39	3.6	2.61	2.72	2.845	0.79	0.51	0.62	0.54	0.76	0.56	0.94	0.68	0.7	0.68
Organic C (%)	2	1.92	1.82	1.64	3.41	3.94	2.38	2.63	2.19	0.12	0.1	0.12	0.09	0.09	0.13	0.33	0.09	0.14	0.12
Inorganic C (%)	0.97	1.72	0.26	0.18	-0.02	-0.34	0.23	0.09	0.205	0.67	0.41	0.5	0.45	0.67	0.43	0.61	0.59	0.56	0.56
N (%)	0.15	0.14	0.16	0.16	0.28	0.3	0.23	0.25	0.195	0.02	0.02	0.02	0.02	0.02	0.02	0.03	0.02	0.03	0.02
Mo (mg/kg)	8.6	6.51	8.36		9.43	9.02	6.69	7.86	8.36	ND	0	0	0	0	0	0	0	0	0
Zr (mg/kg)	409.22	421.56	469.67	428.04	529.16	545.62	513.07	478.23	473.95	ND	341.01	323.78	370.58	344.9	387.1	359.04	340.76	449.73	344.9
Sr (mg/kg)	79.56	71.31	113.49	182.08	79.92	79.1	92.09	87.62	83.77	ND	81.01	137.47	88.18	133.09	89.42	102.35	95.33	214.77	95.33
Rb (mg/kg)	116.69	104.61	133.72	127.54	105.24	107.75	138.25	135.67	122.115	ND	82.33	90.04	87.3	94.09	73.95	96.07	93.48	87.71	87.71
Th (mg/kg)	9	7.23	13.34	7.43	12.5	6.43	12.36	11.32	10.16	ND	6.78	8.45	7.66	8.9	6.3	7.9	9.72	9.76	7.9
Pb (mg/kg)	70.76	58.83	44.84	46.53	64.83	66.93	64.05	72.18	64.44	ND	12.93	20.97	14.99	21.22	20.87	17.79	17.64	18.22	17.79
As (mg/kg)	0	0	0	0	0	0	14.19	9.21	0	ND	0	0	0	0	0	0	15.44	0	0
Zn (mg/kg)	162.26	149.57	136.89	164.69	118.2	113.3	148.01	112.81	142.45	ND	25.96	45.2	58.87	54.29	48.02	81.45	52.31	38.36	48.02
Cu (mg/kg)	0	0	0	0	0	0	0	0	0	ND	0	0	0	0	0	0	38.04	0	0
Fe (mg/kg)	36252.51	30332.54	50599.13	51316.76	39764.49	37181.05	47464.75	47264.09	43514.29	ND	20513.27	29002.92	26570.57	27564.56	24284.97	29156.5	28828.42	27676.66	27564.56
Mn (mg/kg)	350.19	237.54	446.49	429.59	229.51	225.1	366.13	285.07	317.63	ND	307.37	497.14	417.92	446.45	487.19	418.94	529.87	554.14	446.45
Cr (mg/kg)	89.86	91.25	106.78	75.45	85.6	69.24	88.2	107.89	89.03	ND	35.43	54.52	60.36	63.81	63.28	83.21	56.23	64.33	60.36
V (mg/kg)	107.25	94.42	146.04	171.69	123.72	112.08	125.39	158.92	124.555	ND	64.26	69.09	80.31	107.45	74.36	113.5	0	0	69.09
Ti (mg/kg)	4525.35	4080.57	6038.47	5328.28	4806.53	5135.39	5854.74	5354.74	5231.835	ND	3644.35	3919.37	3757.93	3885.45	3684.74	4064.97	4057.87	3549.6	3757.93
Ca (mg/kg)	16333.82	30028.46	3695.49	4486.5	2446.89	2333.41	2513.84	2279.02	3104.665	ND	5674.03	5378.65	10340.13	10863.56	4818.47	10673.78	10789.59	7799.96	7799.96
K (mg/kg)	26082.6	22990.93	28496.87	24558.86	20613.74	21152.59	26351.85	25239.49	24899.175	ND	21299.88	22244.42	22920.13	22882.95	18026.28	23963.61	25010.46	21343.74	22244.42
S (mg/kg)	0	0	0	0	0	0	0	0	0	ND	0	0	0	0	0	0	0	0	0
Ba (mg/kg)	362.66	366.06	354.17	348.06	324.75	182.73	286.87	458.8	351.115	ND	402.24	468.31	563.74	516.83	441.66	451.31	519.67	403.41	451.31
Cs (mg/kg)	0	0	0	0	0	0	0	0	0	ND	37.68	56.85	64.95	36.97	52.43	46.09	59.03	41.35	46.09
Te (mg/kg)	0	0	0	0	0	0	0	0	0	ND	51.61	63.64	53.67	41.56	62.59	57.61	73.06	66.15	57.61
Sb (mg/kg)	0	0	0	0	0	0	0	0	0	ND	0	0	35.56	0	0	0	0	0	0
Sn (mg/kg)	0	0	0	0	0	0	0	0	0	ND	0	19.77	20.36	0	15.69	0	18.72	18.9	15.69
Nb (mg/kg)	22.02	17.87	24.97	20.98	20.66	22.31	25.89	21.22	21.62	ND	11.13	13.03	11.62	14.86	11.27	13.65	14.23	16.49	13.03
Bi (mg/kg)	0	0	0	0	13.59	0	11.28	0	0	ND	0	0	0	0	0	0	11.29	10.05	0
Al (mg/kg)	3806.97	2600.11	5521.66	3836.64	3415.75	4085.53	3583.11	1312.21	3695.04	ND	3736.72	4464.52	3316.66	4103.7	3970.87	4165.91	5102.2	4040.68	4040.68
P (mg/kg)	0	0	0	0	0	0	210.19	0	0	ND	0	0	0	0	0	0	0	0	0
Si (mg/kg)	49232.02	46557.92	46030.07	39215.66	50117.81	50135.76	45713.93	36396.9	46293.995	ND	65254.17	55194.71	46004.35	53201.49	61991.16	49361.21	53785.68	56334.87	53785.68
Cl (mg/kg)	517.4	488.35	524.08	558.59	535.39	480.77	606.11	537.97	529.735	ND	429.91	469.46	493.7	474.53	513.02	517.52	437.19	471.76	471.76

2 SUPPLEMENTARY FIGURES

Note: Max-normalised ratio considers one contig across all samples, where the value is normalized to the single maximum value for that contig. The sample containing the contig that contributed the maximum value will always equal 1, and the value for that contig in the other samples will be the fraction of that maximum.

Abundance values in anvi'o are calculated as the mean coverage of each MAG divided by that sample's overall mean coverage across all the MAGs. Abundance values therefore represent the ratio of a MAGs mean coverage to the mean coverage of all the MAGs in the sample. Therefore, MAGs with larger abundance values are more represented in that sample (i.e. recruited more reads) than those contigs with smaller abundance values. This is a useful view for highlighting abundant and rare taxa.

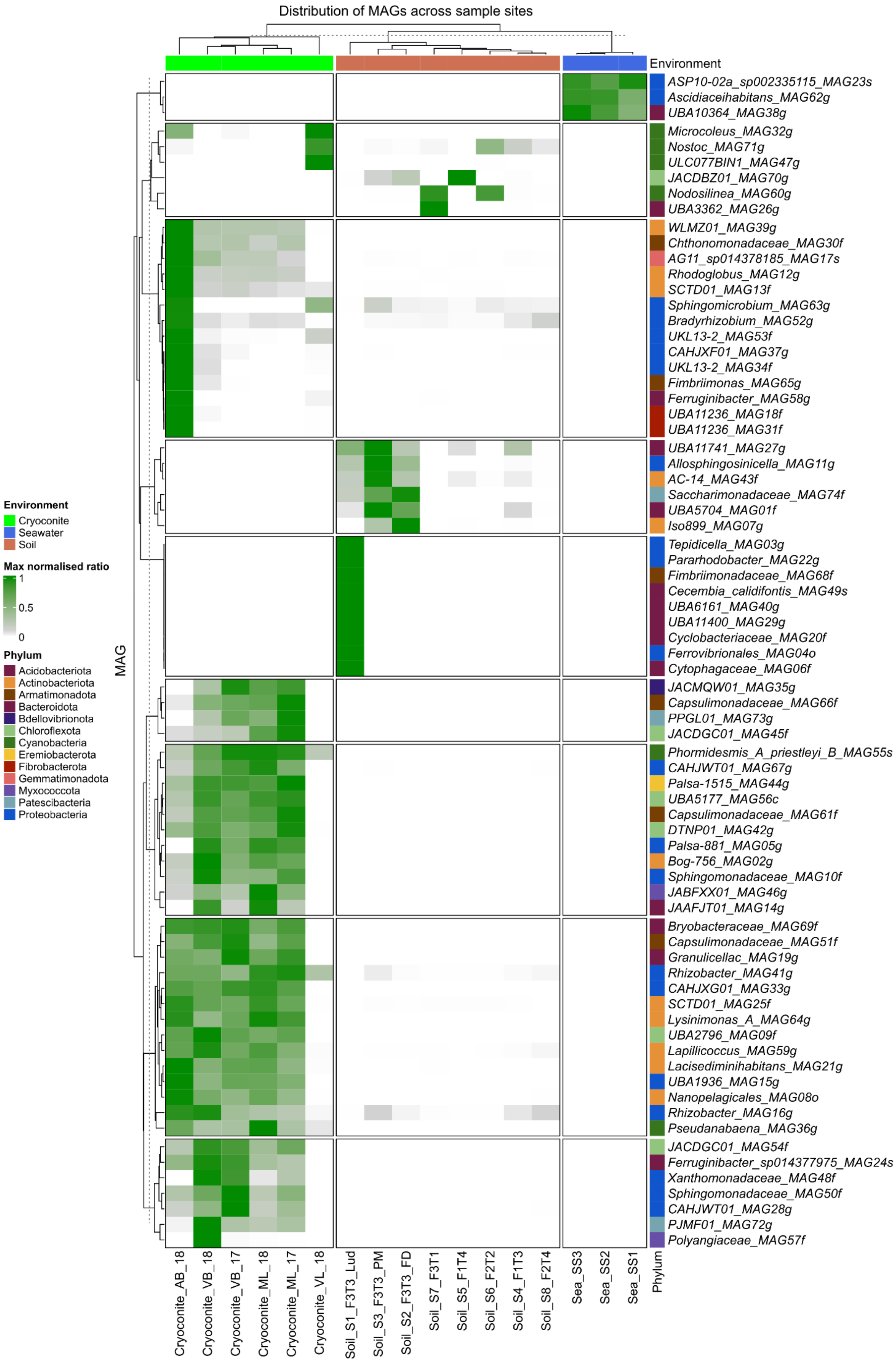

Supplementary Figure 1: The spatial distribution of MAGs visualised using a heatmaps of max-normalised ratio (number of reads recruited to a contig divided by the maximum number of reads recruited to that contig in any sample).

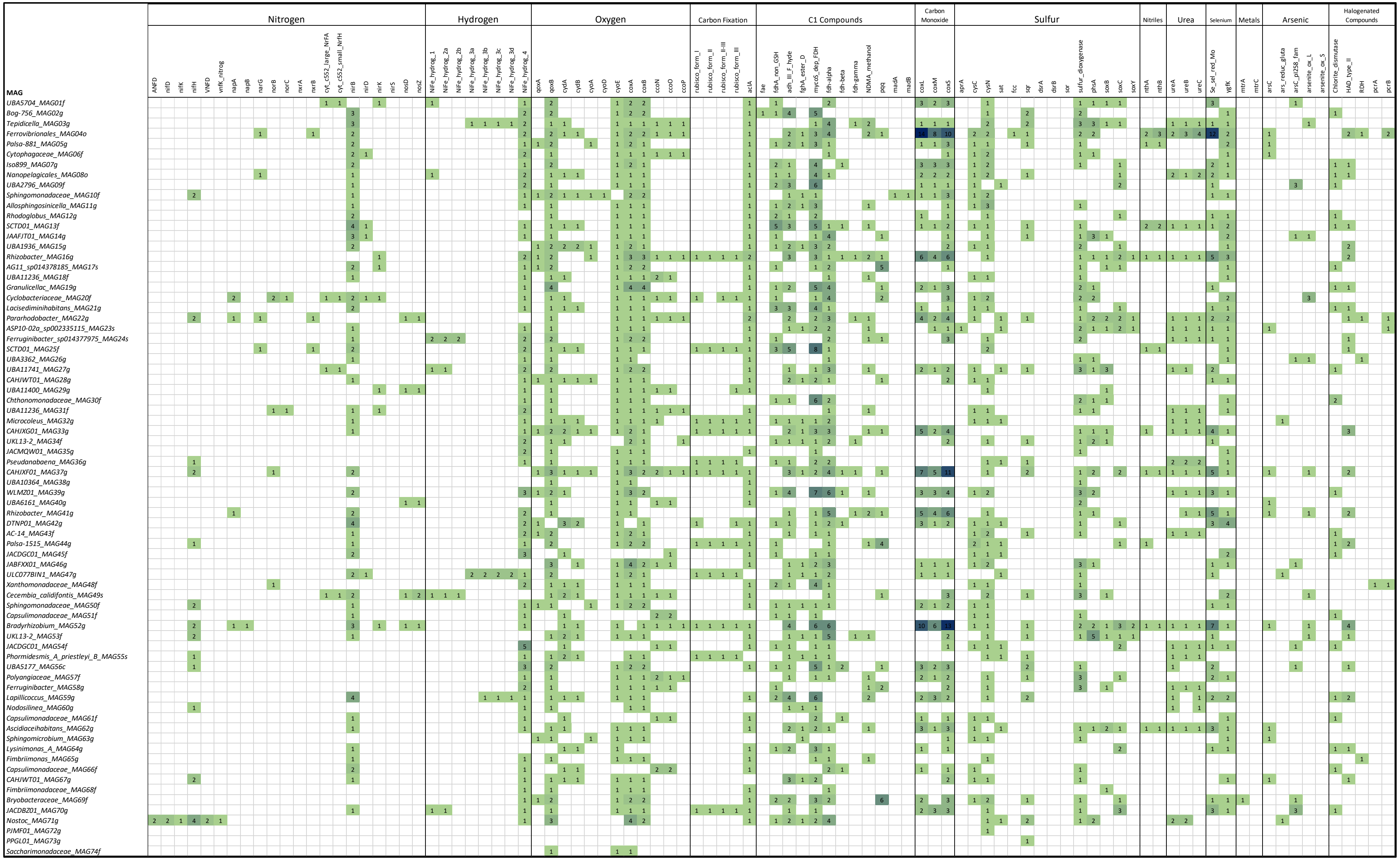

Supplementary Figure 2: This Figure uses .HMMs from **(Anantharaman et al., 2016)**. This Figure is generated from the "Search with HMMs of Environmental Bioelement families - v1v1.8.0" on KBase using more permissive HMMer thresholds than MetabolisHMM (Figure 5). These results can be explored on the public KBase narrative: "Svalbard-MAGS" at https://narrative.kbase.us/narrative/155329.

2.1 PHYLOGENETIC TREES

Phylogenetic trees are created using MUSCLE for multiple sequence alignment, and FastTree to generate trees. The final trees were visualised on Interactive Tree of Life (ITOL) (https://itol.embl.de).

Phylogenetic trees are provided for a subset of the HMM markers identified using metabolisHMM tool available at https://github.com/elizabethmcd/metabolisHMM (McDaniel et al., 2019).

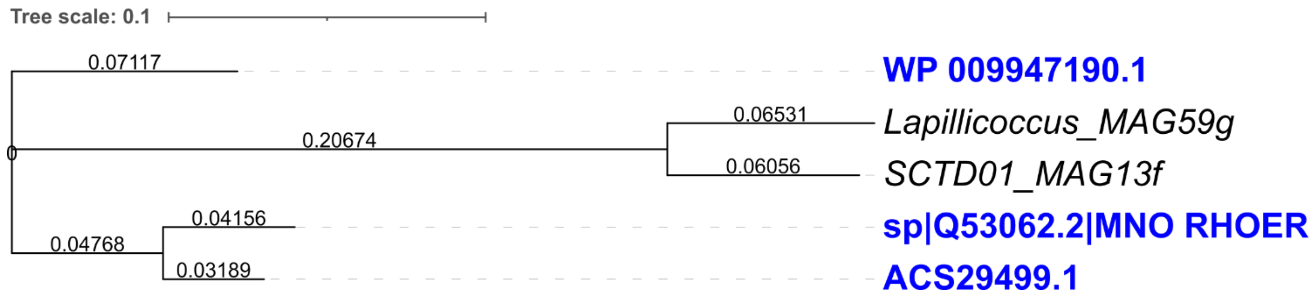

Supplementary Figure 3 Phylogenetic tree of HMM hits to NDMA-dependent methanol dehydrogenase (ndma) gene (TIGR04266). MetabolisHMM identified two Actinobacterial MAGs with a ndma gene (MAG_059_g_Lapillicoccus and MAG_013_f_SCTD01). Reference sequences in in **blue bold**.

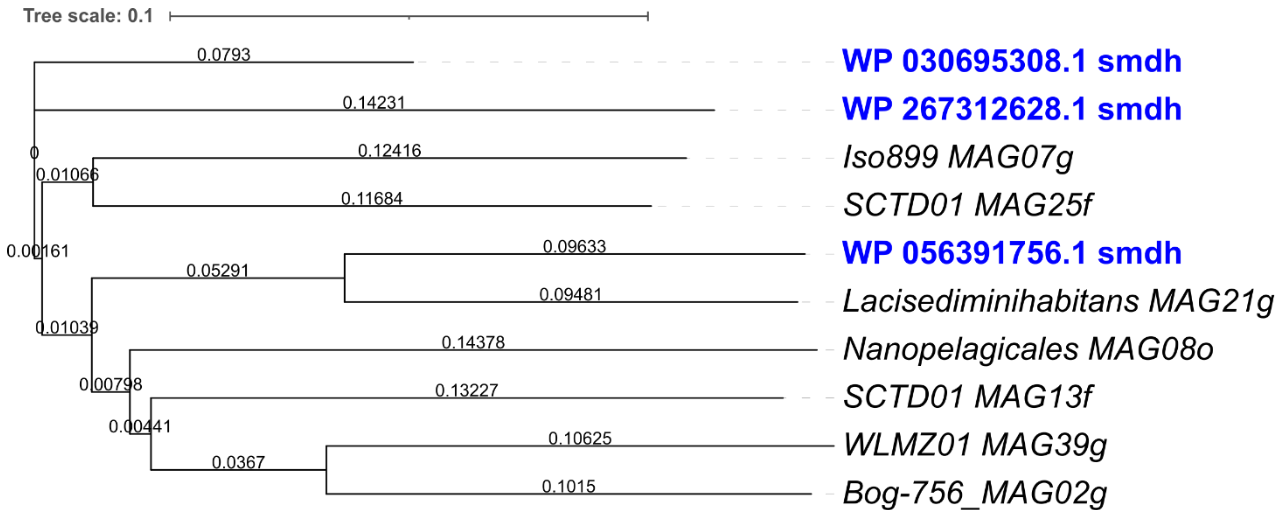

Supplementary Figure 4 Phylogenetic tree of HMM hits to S-(hydroxymethyl)mycothiol dehydrogenase (smdh) gene (TIGR03451). Reference sequences in in **blue bold**.

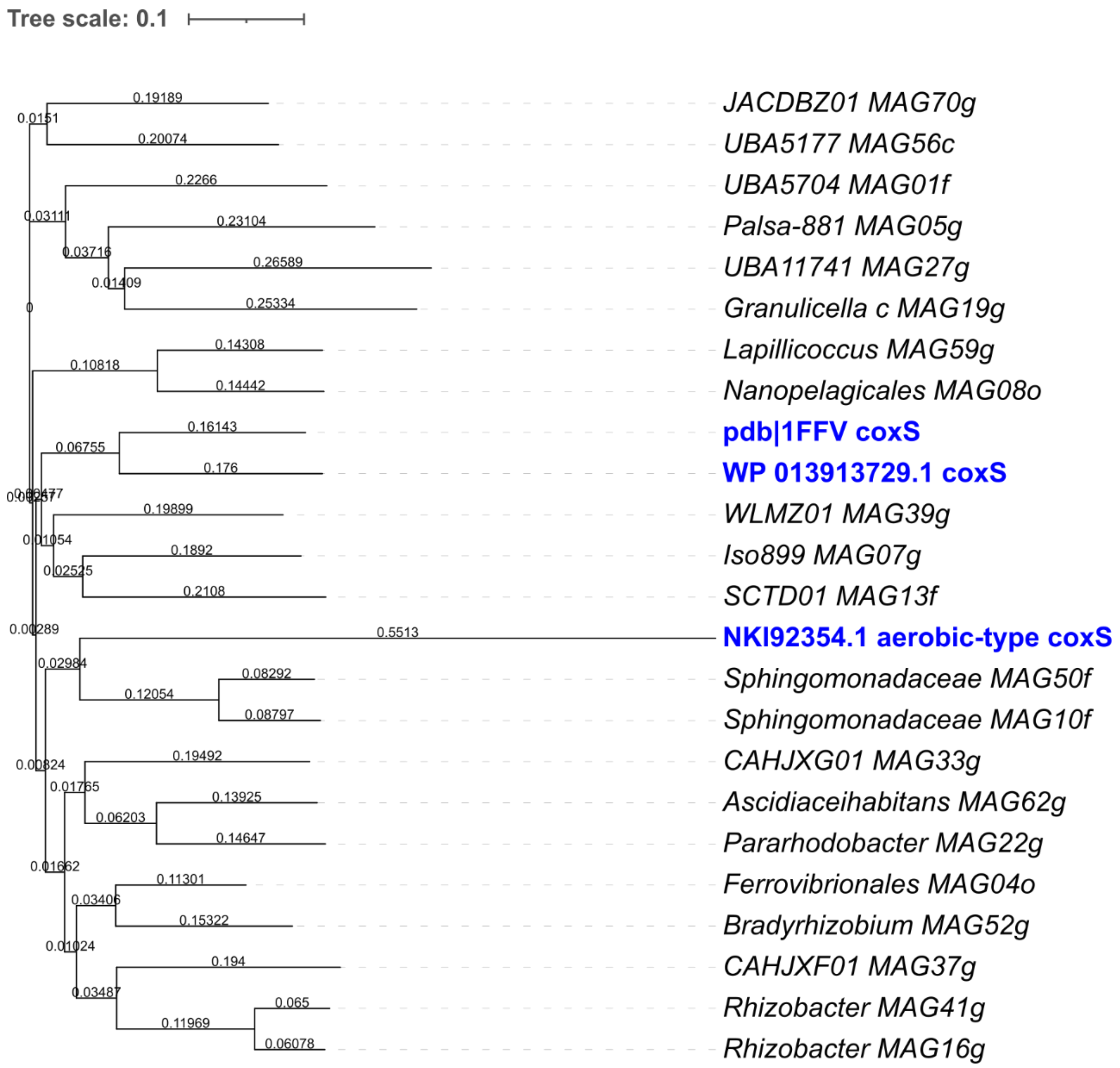

Supplementary Figure 5 Phylogenetic tree of HMM hits to carbon monoxide dehydrogenase small subunit gene (coxS). Reference sequences in in **blue bold**.

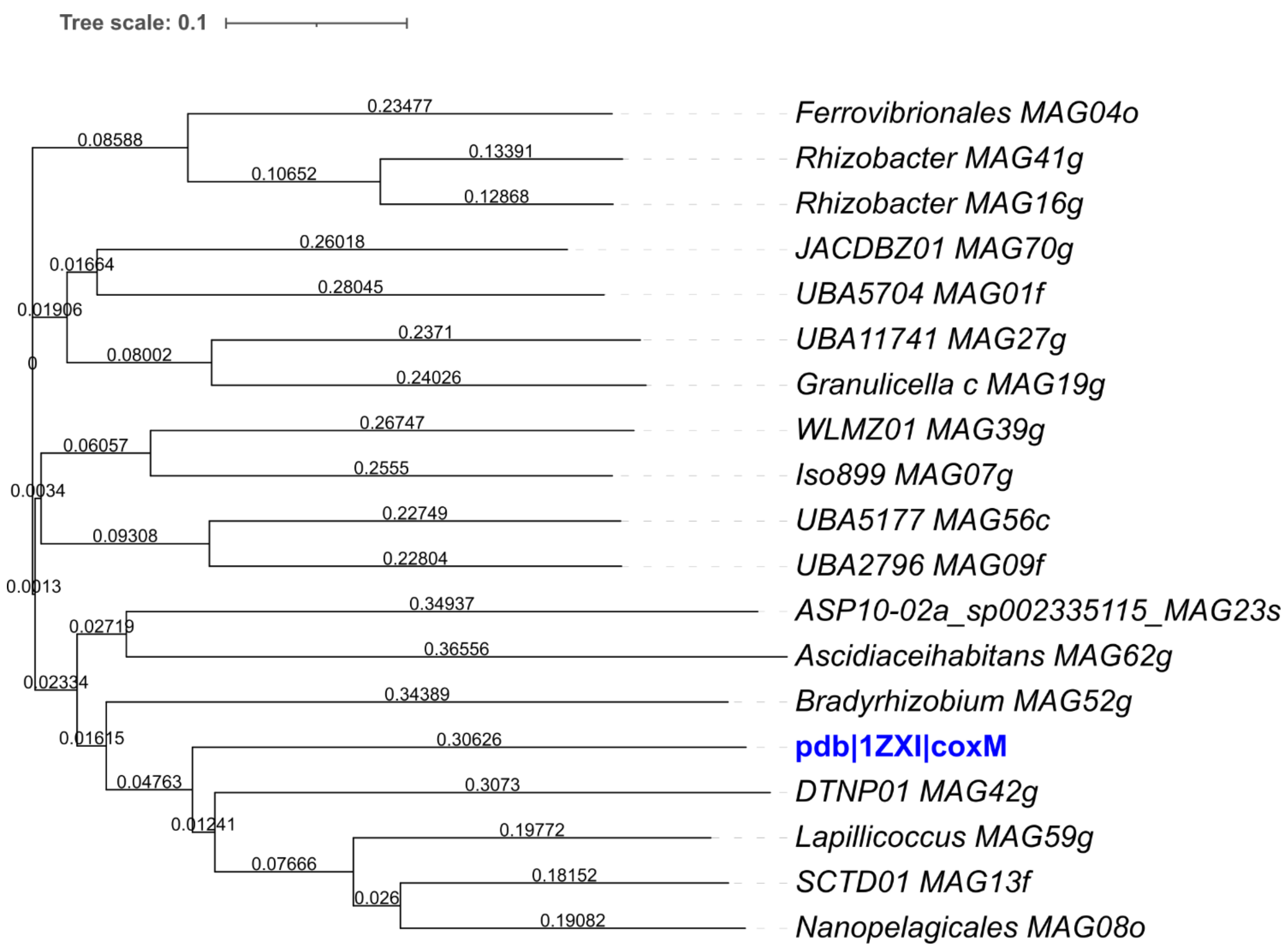

Supplementary Figure 6 Phylogenetic tree of HMM hits to carbon monoxide dehydrogenase medium subunit gene (coxM). Reference sequences in in **blue bold**.

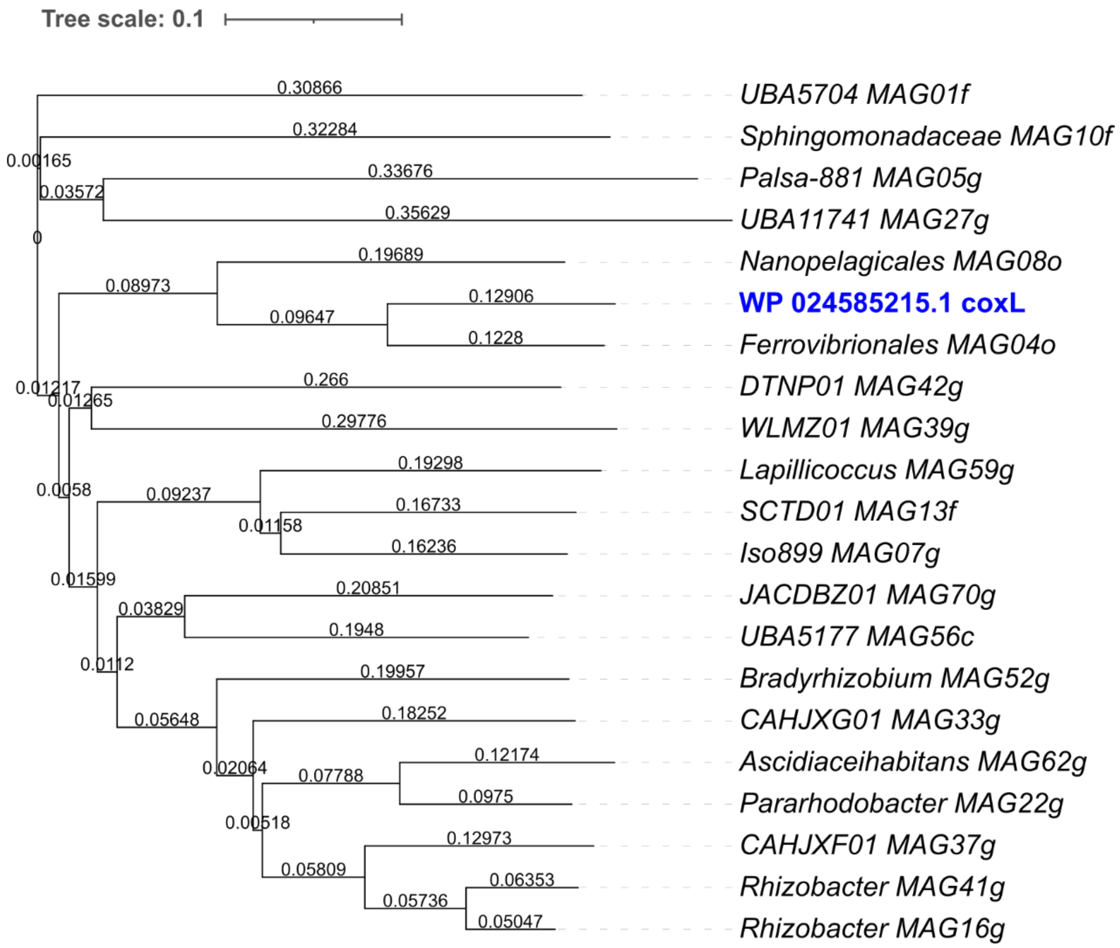

Supplementary Figure 7 Phylogenetic tree of HMM hits to carbon monoxide dehydrogenase small subunit gene (coxL). Reference sequences in **blue bold**.

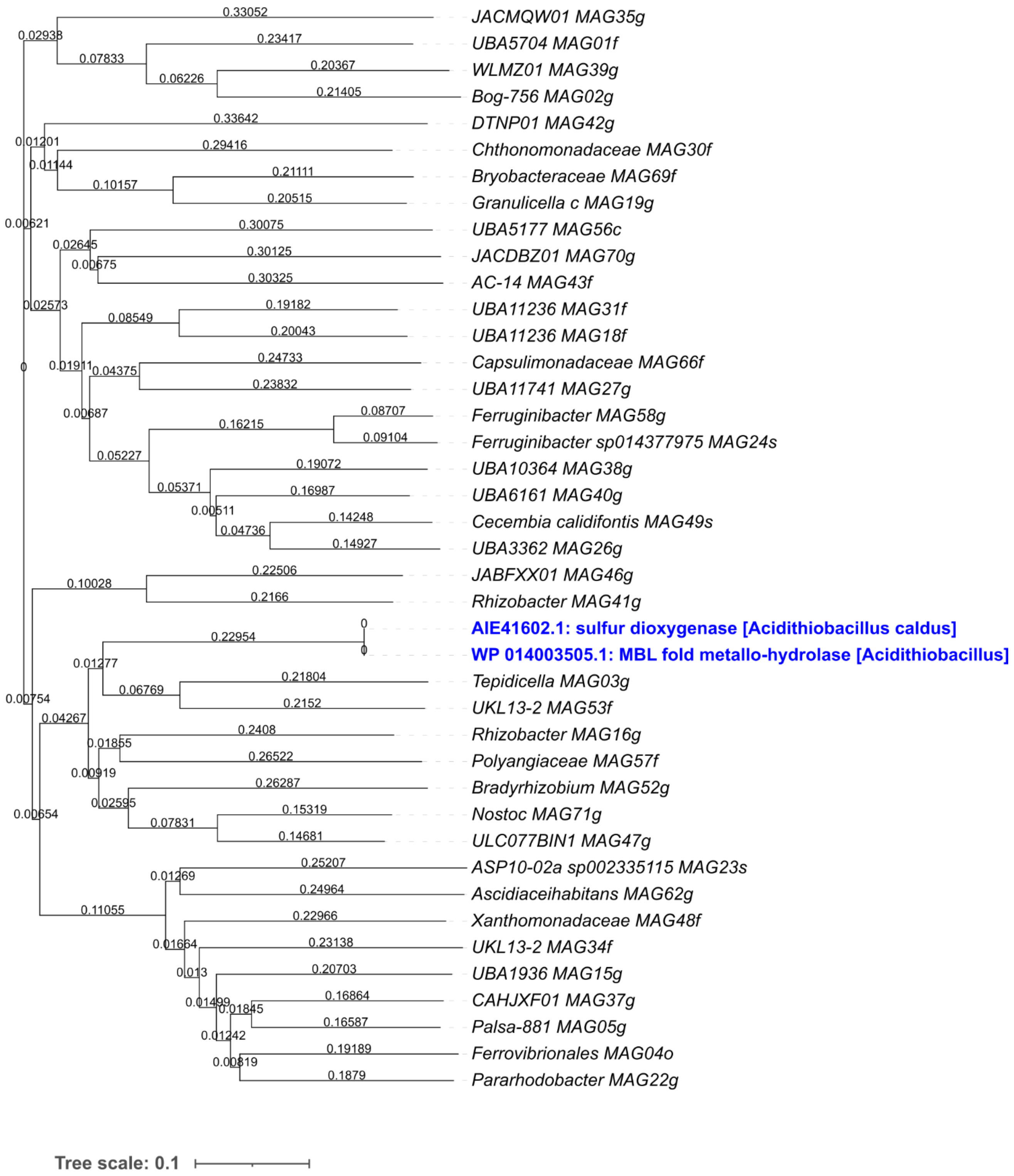

Supplementary Figure 8 Phylogenetic tree of HMM hits to sulfur dioxygenase (sdo) gene. (Based on protein sequence). Reference sequence in **blue bold**.

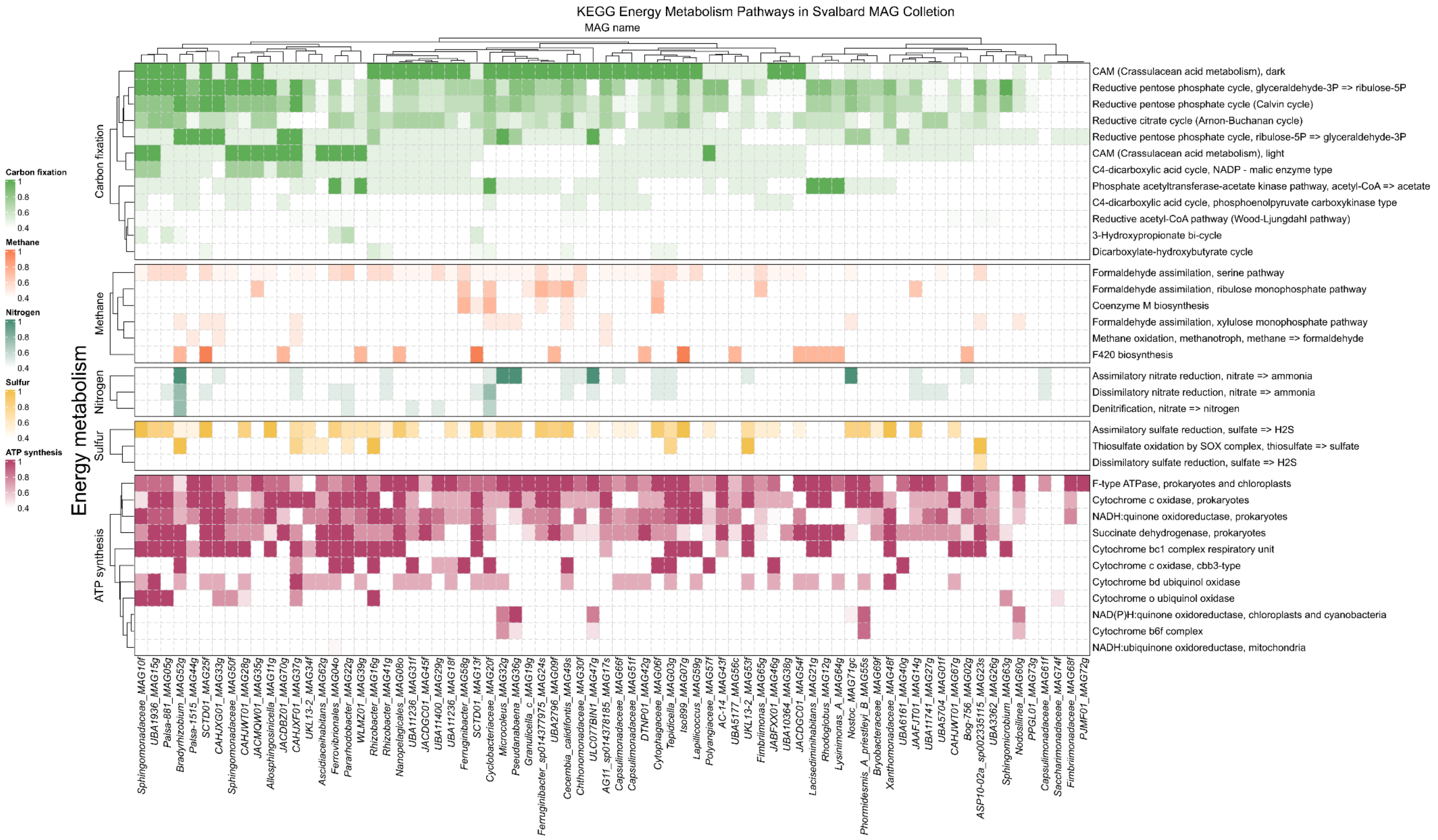

Supplementary Figure 9: Energy Metabolism in MAGs based on KEGG pathways (only pathways > 0.4% complete are shown)

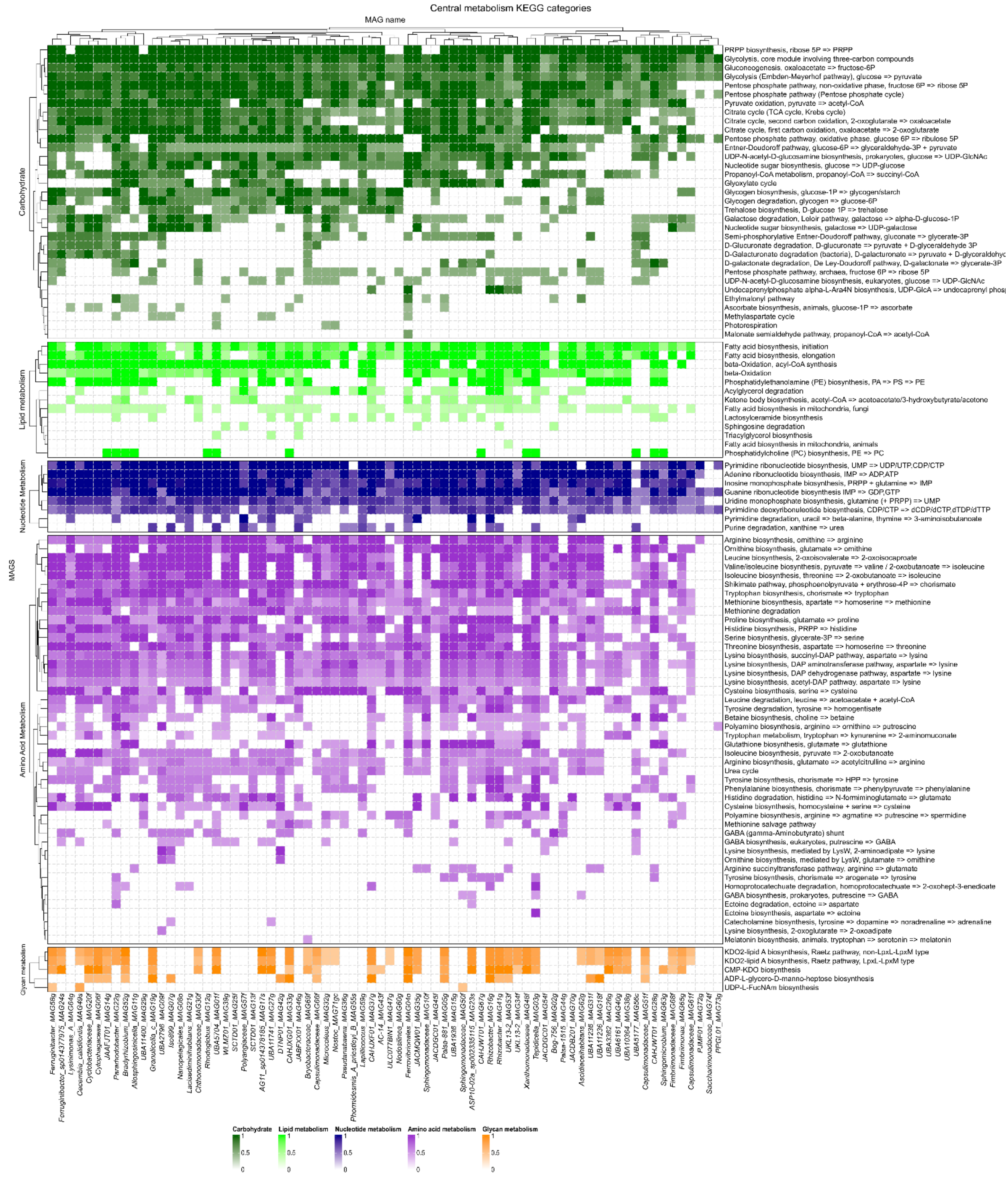

Supplementary Figure 10: Central Metabolism in MAGs based on KEGG pathways (only pathways > 0.4% complete are shown)

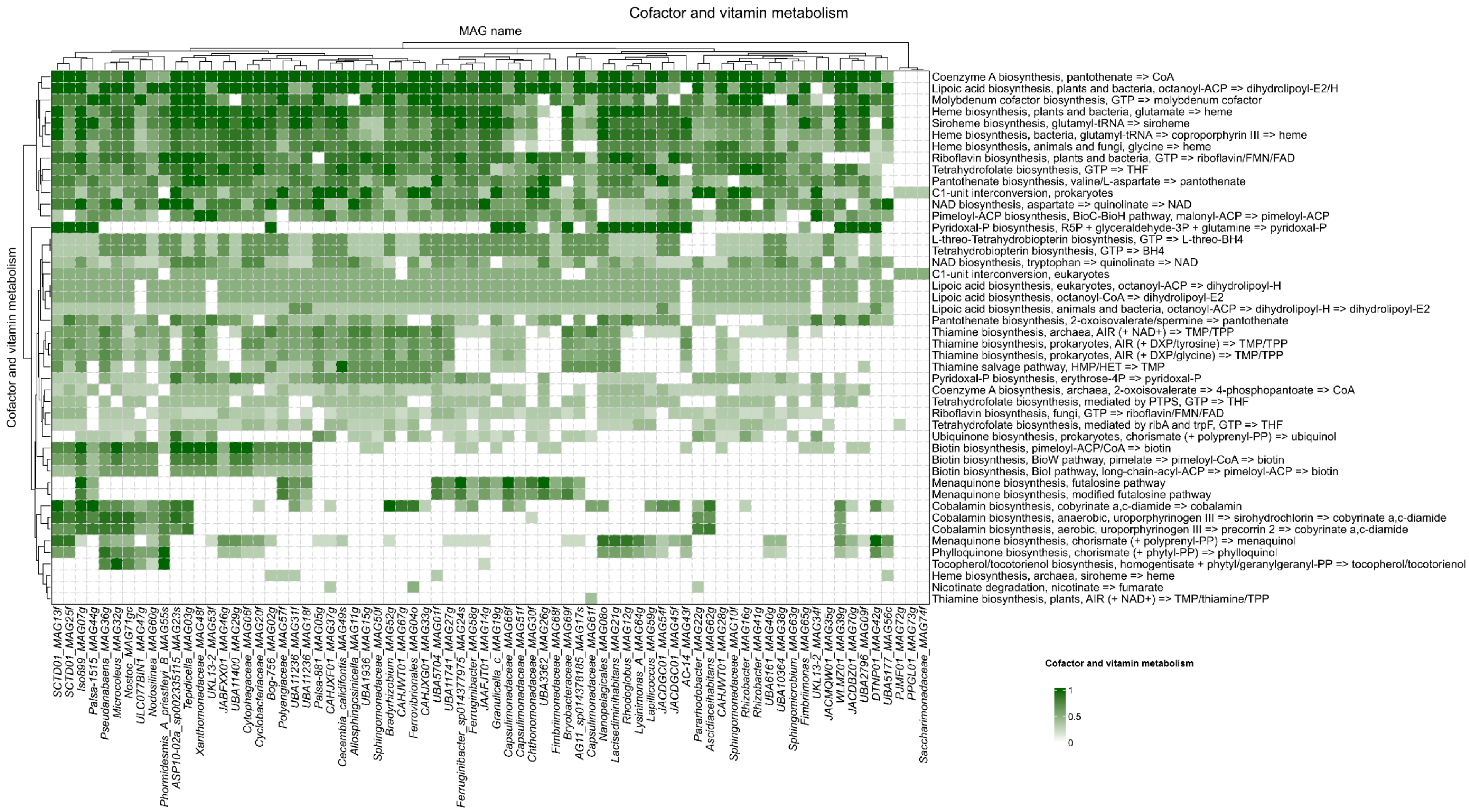

Supplementary Figure 11 Cofactor and vitamin metabolism. Heatmap of cofactor and vitamin metabolism in all the MAGS.

3 SUPPLEMENTARY INFORMATION

3.1 DETAILED DNA EXTRACTION METHODS

3.1.1 Qiagen DNEasy PowerWater

The Qiagen DNeasy® PowerWater® Sterivex™ Kit was used to extract DNA samples that had been filtered through Sterivex filters (Millipore cat. no. SVGPL10RC).

Prior to use, Solution ST1A was added to Solution ST1B, and mixed well. Inlet and outlet caps were removed to extract the RNA*later*™ Stabilization Solution (ThermoFischer Scientific) which had been added to the Sterivex filters for storage. Liquid was removed using a syringe with a tubing connector that had been washed in bleach, HCl and ddH_2O. The outlet cap was replaced and 0.9 mL of Solution ST1B was added using a pipette tip. Thereafter, the inlet was recapped and the Sterivex filter were secured horizontally to a vortex adapter, with the inlet facing outwards. The filters were vortexed at minimum speed for 5 min, after which they were rotated 180 degrees from the original position, while maintaining their orientation with inlet facing outwards, and vortexed for an additional 5 minutes. The Sterivex filter units were positioned with the inlet facing up, the inlet caps were removed, and 0.9 mL of Solution MBL (prewarmed to 65°C) was added to each unit using a pipette tip. The inlets were recapped, and the Sterivex filter units were incubated at 90ºC for 5 minutes. The filters were cooled for 2 minutes and then secured horizontally, with the inlet facing outwards to a vortex adapter and vortexed at maximum speed for 5 minutes. The lysate was removed from the Sterivex filters by pulling back the plunger of a 3 mL syringe to fill the barrel with 1 mL of air, and then attaching it to the inlet of Sterivex filter unit. The air was pushed into the unit until there was resistance, and then the plunger was released and slowly pulled back to remove as much of the lysate as possible. The syringe was detached from the Sterivex filter unit, and the lysate added to 5 mL glass PowerBead Tubes. The PowerBead Tubes were secured horizontally to a vortex adapter and vortexed at maximum speed for 5 minutes. The tubes were centrifuged at 4000 x g for 1 minute and the supernatants transferred to clean 2.2 mL collection tubes. Thereafter, 300 µL of Solution IRS was added to the tubes, which were vortexed briefly to mix, and then incubated in an ice-bucket (approximately 4 °C) for 5 minutes. The tubes were centrifuged at 13,000 x g for 1 minute and the supernatants were transferred to clean 5 mL collection tubes.

For each sample, a tube extender was placed firmly into an MB Spin Column and the tube extender/MB Spin Column unit was attached to a VacConnector and VacValve (VV) on the QIAvac 24 Plus Manifold. Solution MR, which had been prewarmed to 65 °C, was retrieved and 3 mL was immediately added to the Collection Tubes containing supernatant and vortexed to mix. The entire 4.5 mL of supernatant was then loaded into the tube extender/MB Spin Column. The vacuum source was turned on and the VV of the port opened, allowing the lysate to pass through. Once the lysate had passed through completely, the VV was closed. While keeping the MB Spin Column attached to the VV the tube extenders were removed and discarded. Following this, 0.8 mL of ethanol was added to the MB Spin Column, the VV was opened, and the ethanol allowed to pass through the column completely. The VV was closed and then 0.8 mL of Solution PW (mixed thoroughly by shaking) was added to the MB Spin Column. The VV was opened, and Solution PW allowed to pass through the column completely. The vacuum was maintained for another minute to dry the membrane. The VV was closed, and 0.8 mL of ethanol was added to the MB Spin Column. The VV was opened, and a vacuum was applied until the ethanol had passed through the MB Spin Column completely. The vacuum was maintained for another minute to dry the membrane, then closed. The vacuum source was turned off and an unused port as

opened to vent the manifold. The MB Spin Column was removed and placed in a 2.2 mL collection tube. The tube was centrifuged at 13,000 x g for 2 min to completely dry the membrane. The MB Spin Column was transferred to a new 2.2 mL collection tube and 100 µL of Solution EB was added to the centre of the white filter membrane. Finally, the tubes were centrifuged at 13,000 x g for 1 minute at room temperature to elute the DNA and the MB Spin Columns were discarded. DNA concentration and quality were tested using Qubit and agarose gel electrophoresis before long-term storage at -20 °C.

3.1.2 Qiagen DNeasy PowerSoil

The Qiagen DNeasy® PowerSoil® Kit was used to extract DNA from cryoconite. Cryoconite DNA from these extractions were used for the shotgun metagenomes.

Briefly, approximately 0.25 g of cryoconite was added to PowerBead tubes and vortexed briefly to mix. Solution C1 was pre-warmed to 60 °C and 60 µL was added to each tube. The tubes were then secured horizontally to a Vortex Adapter and vortexed at maximum speed for 15 minutes. The samples were centrifuged at 10 000 x g for 30 seconds and the supernatant transferred to 2 mL collection tubes to which 250 µL of Solution C2 had been pre-aliquoted. The samples were vortexed for 5 seconds and then incubated in an ice-bucket (4 °C) for 5 minutes. The samples were centrifuged at 10 000 x g for 1 minute and 600 µL of the supernatants were added to a 2 mL collection tube to which 200 µL of Solution C3 had been pre-aliquoted. The tubes were vortexed to mix, incubated in an ice-bucket (4 °C) for 5 minutes and then centrifuged at 10 000 x g for 1 minute. Carefully avoiding the pellet, 750 µL of supernatant was transferred to 2 mL collection tubes to which 1200 µL of vigorously mixed Solution C4 had been pre-aliquoted. The samples were vortexed briefly to mix and then 675 µL was added onto an MB Spin Column and centrifuged at 10 000 x g for 1 minute. The throughflow was discarded and the same step repeated twice more until all the sample had been processed. Thereafter, 500 µL of Solution C5 was added to the Spin Column and the samples were centrifuged for 30 seconds at 10 000 x g. The flow through was discarded and the Spin Column was centrifuged for a further minute at 10 000 x g. Following this, the Spin Column was carefully transferred to a clean 2 mL collection tube and 100 µL of Solution C6 (10 mM Tris-HCl, pH 8.5) was added to the centre of the filter membrane and left for 5 minutes at room temperature. The DNA was eluted by centrifugation for 30 seconds at 10 000 x g. The Spin columns were discarded, and the DNA concentration and quality were tested using Qubit and agarose gel electrophoresis before long-term storage at -20 °C.

3.1.3 FastDNA™ Spin Kit for Soil

The FastDNA™ Spin Kit for Soil (MP Biomedicals) was used to extract DNA from glacier forefield soils. Kit was selected because it yielded higher concentrations of DNA than the Qiagen DNEasy PowerSoil, particularly for the high clay-content soils close to the glacier snout.

Extractions from forefield soil were performed following the manufacturer's instructions with small modifications. Approximately 500 mg of soil was weighed and added to Lysing Matrix E tubes, to which 978 µL of Sodium Phosphate Buffer and 122 µL of MT Buffer was added. The samples were then put on ice and homogenized in the FastPrep24 Instrument for three cycles of 30 seconds at a speed setting of 6.0, with 30 seconds of cooling on ice between cycles. The tubes were centrifuged for 15 minutes at 14 000 x g to pellet debris. Following this, the supernatant was transferred to a clean 2mL microcentrifuge tube, to which 250 µL PPS (Protein Precipitation Solution) was added. The tubes were inverted 10 times to mix, followed by 5 minutes centrifugation at 14 000 x g to pellet the precipitate. The supernatant was then carefully transferred to 15ml flacon tubes. The Binding Matrix suspension was mixed thoroughly using a vortex, and 1 mL was added to the supernatant in each 15 mL tube. Tubes were inverted by hand for 2 minutes, and then placed in a rack for 3 minutes for the silica matrix to settle. Thereafter, 500 µL of supernatant was removed and discarded, avoiding the silica pellet. The Binding Matrix was gently resuspended in the remaining supernatant and approximately 600 µL was added to Spin™ Filter. The Spin™ Filter was centrifuged at 14 000 x g for 1

minute, and the catchment tube was emptied. The remaining supernatant was added to the Spin™ Filter, and the centrifugation and emptying step was repeated. The pellet on the filter was gently resuspended using 500 µL SEWS-M (to which ethanol had been added at first use). The Spin™ Filter tube was then centrifuged at 14 000 x g for 1 minute, the catchment tube was emptied and replaced, followed by a second centrifugation of 2 minutes at 14 000 x g to dry the matrix. The catch tube was discarded and replaced with a new catch tube. The Spin™ Filter was then allowed to air dry at room temperature for 5 minutes. Finally, the Binding Matrix was gently resuspended in 100 µL DES (DNase/ Pyrogen-Free Water) and the tubes were centrifuged for 1 minute at 14 000 x g to elute the DNA, after which the Spin™ Filters were discarded. The DNA concentration and quality were tested using Qubit and agarose gel electrophoresis before long-term storage at -20 °C.

3.1.4 Ludox Density Centrifugation and MasterPure Complete DNA & RNA Purification Kit

The MasterPure™ Complete DNA & RNA Purification Kit was selected to try to extract high molecular weight DNA for cloning because this extraction method does not rely on bead beating, which shears genomic DNA.

3.1.4.1 Ludox Density Gradient centrifugation

To scale up the number of cells that could be separated for lysis, density gradient centrifugation was tried instead of filtering. The method was based on the protocol described by Bakken who separated cells using a colloidal silica gradient (Percoll) (Bakken and Lindahl, 1995). The size of Percoll particles is approximately 35 nm, whilst the size of Ludox HS 40 particles is approximately 12 nm. Although high losses of cells from clay loam cells due to sedimentation through the gradient were reported (Bakken and Lindahl, 1995), the large amount of soil that could be processed might compensate for a relative increase in cell loss. Moreover, although Bakken reported that the direct loading of cell homogenate on top of a Percoll density gradient was not promising, as the cell yield was low, and the gradient contained many contaminating substances; it was speculated that the greater size difference between Ludox HS particles and bacterial cells would allow for easier removal of the silica particles from the bacterial cell pellet.

According to procedures recommended by Bakken & Lindahl, 1995, 60 – 72 g of soil was added to PBS with 0.1% Tween20 or distilled water and homogenized in a waring-type blender for 3 x 1 minute at maximum speed. The slurry was then poured into two sterile 50 mL Falcon Tubes. Thereafter, 100 mL was added, and the process repeated two more times for a total of 6 x 50 mL Falcon Tubes containing 300 mL of slurry. The Tubes were centrifuged at 900 x g for 2 minutes to pellet large soil particles and transferred to a new tube. The Falcon tubes were then centrifuged at 3857 x g for 30 minutes and the supernatant was discarded. The pellet was resuspended in 3.5 mL PBS.

The microbial cell fraction was separated from soil particles using density gradient centrifugation with LUDOX HS 40. Exactly 30 mL of Ludox HS 40 was added to sterile 50 mL Falcon Tubes. The supernatant from three Tubes was loaded onto a single column and the Falcon Tube was centrifuged for 30 minutes at 3857 x g. At this point, the cell fraction formed a visible layer on within the Ludox column, which was collected by pipetting and transferred into a new 50 mL Falcon Tube.

Up to 40 mL of PBS was added to the Tubes, which were then centrifuged for 15 minutes at maximum speed (3857 x g). The supernatant was poured off and the step repeated five times to try and dilute and remove the LUDOX silica particles. After the fifth centrifugation, the supernatant was poured off and the pellet was resuspended in 2 mL PBS and added to a 2 mL microcentrifuge tube. The tube was centrifuged at 10 000 x g for 2 minutes and the supernatant poured off.

3.1.4.1.1 Lysis, and Protein Precipitation

The supernatant was discarded, and the cell pellet resuspended in 300 µL of TE Buffer, to which 2 µL of Ready-Lyse Lysozyme Solution and 1 µL of RNase A was added. The suspension was mixed, centrifuge briefly then incubated at 37°C for 30 minutes. After incubation, 300 µL of 2x Tissue and Cell Lysis Solution and 1 µL of Proteinase K was added to the tube and mixed by vortexing. After a brief pulse-centrifuge to ensure that all the solution is in the bottom of the tube, the suspension was incubated at 65°C for 15 minutes. The tubes were cooled on ice for 3-5 minutes, then 350 µL of MPC Protein Precipitation Reagent was added to the tube and mixed by vortexing vigorously for 10 seconds. The cell debris was pelleted by centrifugation for 10 minutes at 20,000 x in a microcentrifuge at 4°C. The supernatant was transferred to a clean 1.7-mL microcentrifuge tube and 570 µL of isopropanol was added. The contents of the tube were mixed by inverting the tube several times. Thereafter the DNA was pelleted by centrifugation for 10 minutes at 20,000 x g at 4°C. The isopropanol was removed with a pipette tip, being careful not to dislodge the DNA pellet. Once dry, 500 µL of 70% ethanol was added to the pellet, followed by centrifugation for 5 minutes at 20,000 x g at 4°C.21. A pipet tip was used to remove the ethanol without dislodging the DNA pellet. The DNA pellet was then air-dried for 8 minutes at room temperature. Finally, the DNA pellet was resuspended in 40 µL of TE Buffer. The quality (size) and concentration of the isolated DNA was checked by gel electrophoresis on a 1% agarose gel and Qubit, respectively.

3.1.5 MO BIO PowerMax Soil DNA Isolation Kit

The DNA extracted using the MO BIO PowerMax Soil Kit was used in the Soil Shotgun metagenome library F3T3_PM.

Solution C1 was warmed to 60 °C before starting. Fifteen mL of PowerBead Solution was added to a PowerBead Tube followed by10 g of soil sample (from which large stones had been removed). After vortexing for 1 minute, 1.2 mL of Solution C1 was added to the PowerMax® Bead Solution Tube and vortexed vigorously for 30 seconds. The PowerMax® Bead Solution Tubes were then secured horizontally to a vortex and vortexed for 10 minutes at the highest speed, then centrifuged at 2500 x g for 3 minutes at room temperature. The supernatants were transferred to a clean Collection Tube, to which 5 mL of Solution C2 was added, inverted twice to mix, and incubated at 4°C for 10 minutes. The tubes were centrifuged at 2500 x g for 4 minutes at room temperature, then the supernatant was transferred to a clean Collection Tube, avoiding the pellet. Four mL of Solution C3 was added, and the mixture was inverted twice to mix, followed by incubation at 4°C for 10 minutes. The tubes were centrifuged at 2500 x g for 4 minutes at room temperature and the supernatant was transferred to a clean Collection Tube. Solution C4 was mixed by shaking and 30 mL was added to the supernatant and inverted twice. The Spin Filter was filled with the Solution C4/ supernatant mix and centrifuged at 2500 x g for 2 minutes at room temperature. The flow through was discarded and the previous step was repeated twice more with the remaining supernatant. Once the final through-flow has been discarded, 10 mL of Solution C5 was added to Spin Filter and centrifuged at 2500 x g for 3 minutes at room temperature and the flow through discarded. The Spin Filter was centrifuged at 2500 x g for 5 minutes at room temperature, then carefully placed in a new Collection Tube. To elute, 5 mL of sterile Solution C6 was added to the centre of Spin Filter membrane and centrifuged at 2500 x g for 3 minutes at room temperature. The Spin Filter was discarded, and the DNA was further concentrated. To concentrate the DNA, 0.2 mL of 5M NaCl was added, and the tube was inverted 3-5 times to mix. Next, 10.4 mL of 100% cold ethanol was added and inverted 3-5 times to mix. The solution was centrifuged at 2500 x g for 30 minutes at room temperature. All liquid was decanted. The DNA pellet was then washed with 70% cold ethanol, the liquid decanted, and the residual ethanol allowed to evaporate in ambient air. Finally, the precipitated DNA was resuspended in sterile 10 mM Tris.

3.2 SUPPLEMENTARY INFORMATION 1: MANUAL REFINEMENT IN ANVI'O

There are several studies, especially when they involve enormous datasets and thousands of MAGs, that do not go beyond the automatic binning of contigs by binning tools (Parks et al., 2015; Shaiber and Eren, 2019). However, automatic binning tools can often get contig membership wrong, and here, anvi'o provides an excellent tool for the manual refinement of bins (Eren et al., 2015). Every bin in the two datasets was manually refined. Occasionally this was done in an iterative process, where refined bins were rerun through DAS Tool, together with unrefined bins to see whether the newly defined bins scored better or worse than the automatic bins. A comparison between all the different binning methods is shown. Manual refinement was greatly assisted by the method described where the bin membership of each contig was imported as a data layer and could be viewed in anvi-refine. This view enabled several easy refinements that could be performed unambiguously and confidently.

3.2.1 High-quality and poor-quality bins

In a complete and uncontaminated bin, one expects coverage to be even across a single sample, and the relative proportion of reads across samples to be relatively constant. There should be at least one sample that contains every single contig. Example A is a good example of a bin that contains mis-binned contigs. In Example A no samples contain all the contigs. Contigs from several organisms, from different environments and sites, have erroneously been binned together. The mis-binning may have occurred because these are related species, with similar GC content and TNF ratios that resulted in the binning algorithm clustering them together. If this is a large bin with high redundancy, this bin may still be salvaged by and be maintained in the dataset as at least one, and possibly more refined bins.

An example of a high-quality bin is shown in Example B. The coverage is even across a single sample site and relative coverage cross the sample sites is also even. The different binning methods all concur on bin membership. In a poor-quality (incomplete, mixed, or chimeric) bin (Example C), contigs might have different distribution within and across samples. As a result, binning methods might disagree about bin membership of different contigs. These aspects of bin quality do not often come out in summary statistics. However, the visualisation of contigs, and their bin membership quickly highlights ambiguous contigs and bins.

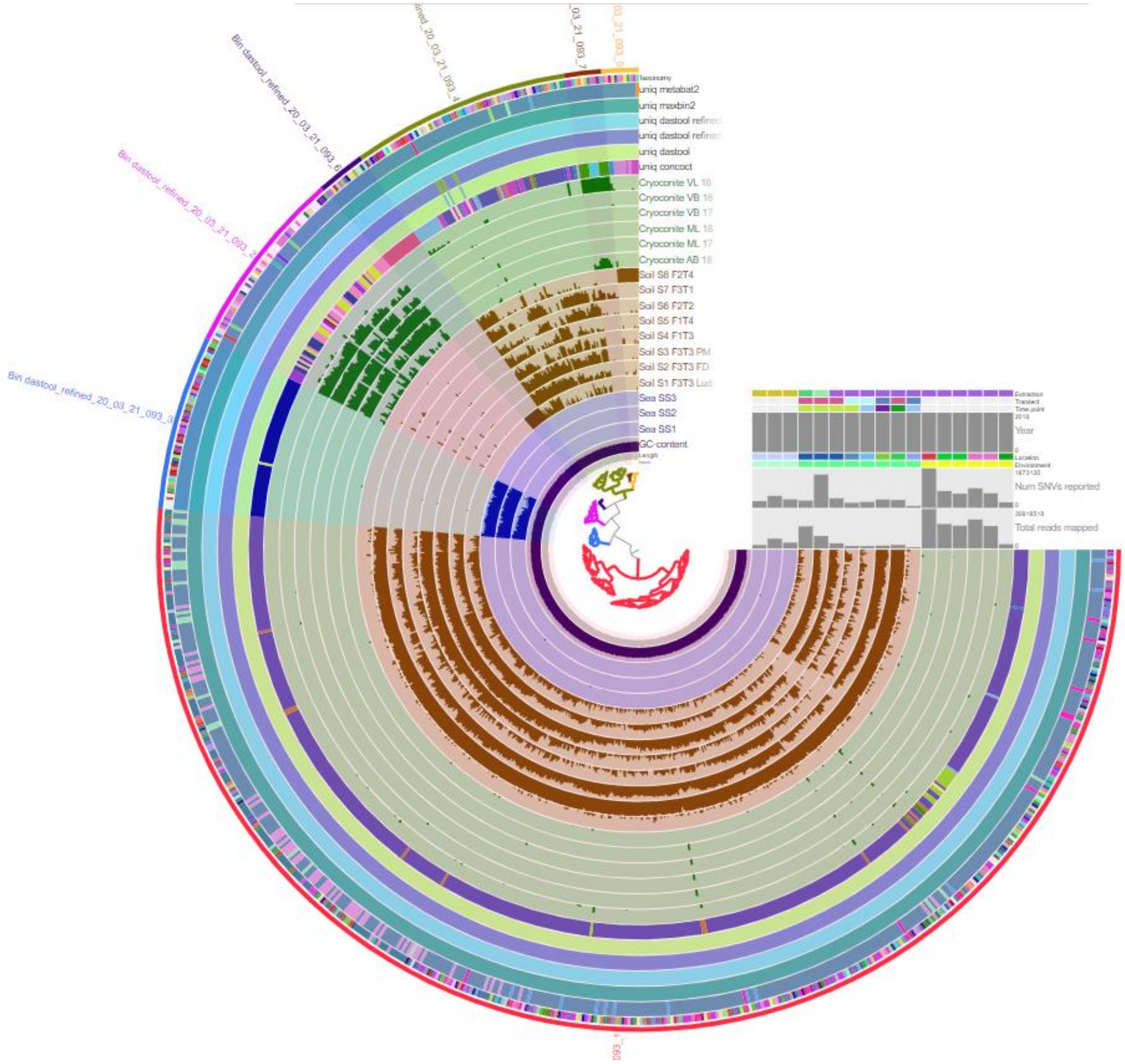

Example A: Bin that is not complete across a single sample and has varying levels of coverage in different samples.

This is a refined DAS Tool bin based off a MaxBin2 bin. The bin is easy to refine based on coverage across different samples. Contigs belonging to seawater species, at least two different cryoconite species and several soil species are easy to discern. This bin is likely not salvageable, as each 'refined' bin from this collection will likely contain too few contigs and be too incomplete to meet miMAG standards. However, refinement is iterative, and the split bins can be viewed and assessed in subsequent rounds.

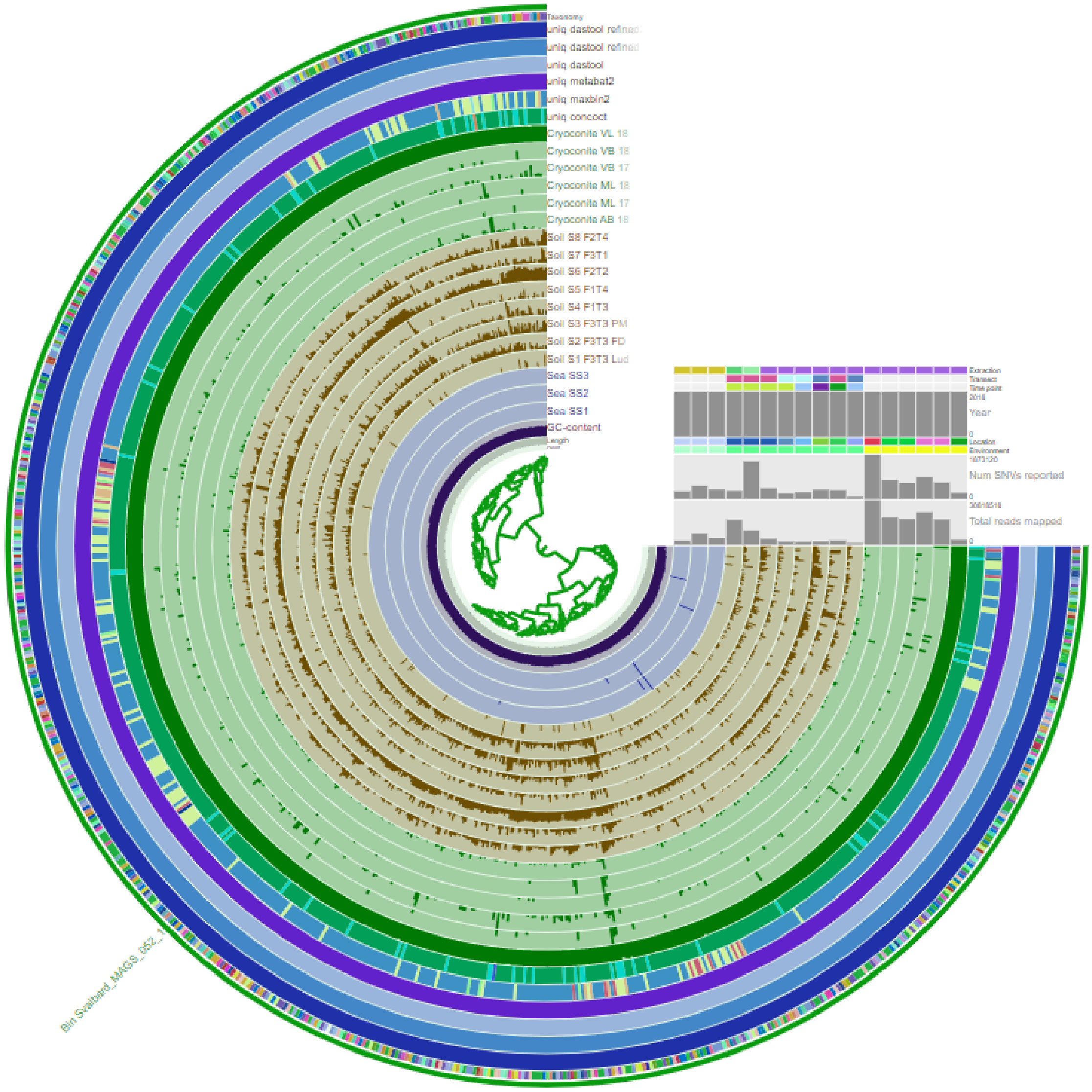

Example B: Bin has high consensus between binning methods and consistent coverage cross a single site.

This bin has high consensus between all the binning tools and even coverage across all of the contigs. DAS Tool selected this bin from a metaBAT2 bin.

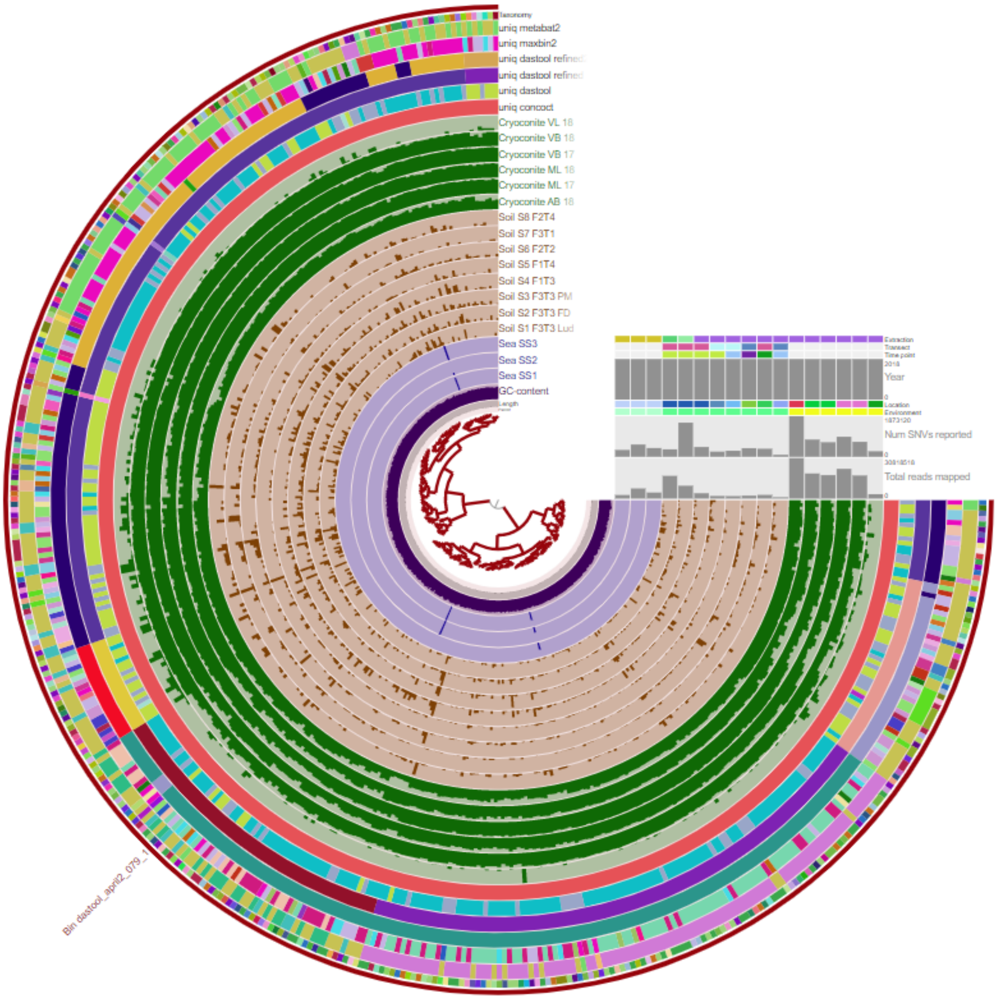

Example C: Bin with low consensus between binning methods, and variable coverage across contigs and across samples.

This bin is from the CONCOCT collection. There is very little consensus between this bin and the other binning tools. CONCOCT has accurately binned contigs with high coverage in cryoconite and low coverage in soil and seawater. However, there is variability in the depth of coverage in the different contigs, and the GC content is also variable. The Kaiju taxonomy is mixed.
